# Novel Insights into the Nobilamide Family from a Deep-Sea *Bacillus*: Chemical Diversity, Biosynthesis and Antimicrobial Activity Towards Multidrug-Resistant Bacteria

**DOI:** 10.3390/md23010041

**Published:** 2025-01-14

**Authors:** Vincenza Casella, Gerardo Della Sala, Silvia Scarpato, Carmine Buonocore, Costanza Ragozzino, Pietro Tedesco, Daniela Coppola, Giovanni Andrea Vitale, Donatella de Pascale, Fortunato Palma Esposito

**Affiliations:** 1 Department of Ecosustainable Marine Biotechnology, Stazione Zoologica Anton Dohrn, Via A.F. Acton, 55, 80133 Naples, Italy; vincenza.casella@szn.it (V.C.); silvia.scarpato@szn.it (S.S.); carmine.buonocore@szn.it (C.B.); costanza.ragozzino@szn.it (C.R.); pietro.tedesco@szn.it (P.T.); daniela.coppola@szn.it (D.C.); giovanniandrea.vitale@unina.it (G.A.V.); donatella.depascale@szn.it (D.d.P.); fortunato.palmaesposito@szn.it (F.P.E.); 2 Department of Chemical, Biological, Pharmaceutical and Environmental Sciences, University of Messina, Viale F. Stagno d’Alcontres, 31, 98166 Messina, Italy

**Keywords:** *Bacillus*, antimicrobials, nobilamides, surfactins, MDR *Staphyloccus aureus*, *Listeria monocytogenes*, biosurfactant, NRPS, genome mining, molecular networking

## Abstract

With rising concerns about antimicrobial resistance, the identification of new lead compounds to target multidrug-resistant bacteria is essential. This study employed a fast miniaturized screening to simultaneously cultivate and evaluate about 300 marine strains for biosurfactant and antibacterial activities, leading to the selection of the deep-sea *Bacillus halotolerans* BCP32. The integration of tandem mass spectrometry molecular networking and bioassay-guided fractionation unveiled this strain as a prolific factory of surfactins and nobilamides. Particularly, 84 nobilamide congeners were identified in the bacterial exometabolome, 71 of them being novel metabolites. Among these, four major compounds were isolated, including the known TL-119 and nobilamide I, as well as the two new nobilamides T1 and S1. TL-119 and nobilamide S1 exhibited potent antibiotic activity against various multidrug-resistant *Staphylococcus* strains and other Gram-positive pathogens, including the foodborne pathogen *Listeria monocytogenes*. Finally, in silico analysis of *Bacillus halotolerans* BCP32 genome revealed nobilamide biosynthesis to be directed by a previously unknown heptamodular nonribosomal peptide synthetase.

## 1. Introduction

During the last decades, the selective pressure caused by the overuse of antibiotics has led to the development of a new class of bacteria, defined as multidrug-resistant (MDR) bacteria. Most of them are resistant to at least three classes of antibiotics and represent a serious threat to human health [1], with the real risk of recreating the pre-antibiotic era. Recently, the World Health Organization (WHO) highlighted the issue represented by antimicrobial resistance, warning of a scary future scenario (https://www.who.int/publications/i/item/9789240093461, accessed on 12 September 2024) [2]. After the COVID-19 pandemic, this phenomenon was defined the “Silent Pandemic” [3]. MDR occurrence has prompted the search for new pharmaceutical leads. Bioprospecting of unexplored environments, such as extreme marine habitat, has emerged as a promising strategy for the discovery of new natural products (NPs), especially those produced by microorganisms.

Marine microbial species have developed several adaptation strategies to survive extreme environmental conditions, often through the production of relevant molecules with biotechnological interest [4,5], such as antibiotics with innovative mechanisms of action [6,7,8,9,10] able to overcome the resistance issue. As a fact, microbial NPs represent the largest source of new antibiotic molecules, covering about two-thirds of the antibacterial therapies approved between 1980 and 2010 for human medicine [11,12].

Among relevant compounds, cell membrane-targeting molecules displaying a “detergent-like” activity represent one of the most promising NPs to prevent or reduce resistance mechanisms [13,14,15]. For example, daptomycin, a cyclic lipodepsipeptide produced by *Streptomyces roseosporus*, is among the last-resort antibiotics for the treatment of Gram-positive pathogens [16,17]. Depsipeptides are chemical compounds with both ester and amide bonds in their structures [18,19]. From a broader perspective, biosurfactants, including lipodepsipeptides, are bacterial membrane-disrupting biomolecules [20].

Today, there is a growing interest in microbial-derived biosurfactants, secreted molecules with the ability to reduce interfacial tension between liquids, showing eco-compatibility, cost-effectiveness, low toxicity, and better selectivity compared to synthetic surfactants [21,22]. They also exhibit antimicrobial, antiviral, and antitumor properties [23,24,25].

Among (marine) microorganisms, the members of *Bacillus* genus are well-known to produce a variety of novel peptides and lipopeptides, which have high potential as antimicrobials and biosurfactants [26,27,28]. These molecules are synthesized by biosynthetic gene clusters (BGCs), i.e., operonic genes, and can be easily predicted by the support of genomic data. In fact, they are usually produced by large multimodular enzymes, known as non-ribosomal peptide synthetases (NRPSs), which work as assembly lines to build peptides by sequential amino acid condensation [29,30]. Non-ribosomal peptides are non-essential compounds, classified as secondary metabolites, that confer advantages by inhibiting competitors and modulating symbiont interactions in challenging environments [31]. Typically, an NRPS module includes adenylation (A), peptidyl carrier protein (PCP), and condensation (C) domains, while the termination module has a thioesterase (TE) domain for releasing and/or cyclizing the peptide. Some modules also feature an epimerization (E) domain to convert amino acids from L- to D-form during synthesis, thereby introducing a mixture of L- and D-amino acids to give peptides a specific stereochemistry for selective interaction with the cellular target [32]. The identification of novel BGCs encoding new active molecules is of crucial importance in drug discovery, as it provides the tools to create sustainable sources of NPs.

The present work describes the screening of around 300 marine bacteria, leading to the identification of a deep-sea *Bacillus* sp. BCP32 with both antimicrobial and biosurfactant activity. Tandem mass spectrometry-based molecular networking analysis of the bacterial exometabolome demonstrated the production of the depsipeptide antibiotic TL-119/A-3302-B [33,34,35,36] and other known congeners reported as nobilamides [37], along with almost 70 new analogues, showing potent antimicrobial activity against drug-resistant Gram-positive pathogens. On the other hand, the high biosurfactant effect has been attributed to the secretion of a rich mixture of surfactins [27,38], well-known lipopeptides from *Bacillus* spp. which have been shown to exert significant antibacterial activity against *S. aureus.* Finally, in silico genome analysis enabled the identification of the previously unknown BGC which encodes the biosynthesis of TL-119/A-3302-B and the nobilamide family. This work emphasizes the importance of exploring marine microbial biodiversity for the discovery and production of novel clinically relevant molecules.

## 2. Results and Discussion

### 2.1. Microbial Collection Primary Screening and Selection of Bacillus sp. BCP32

**Figure 1:**
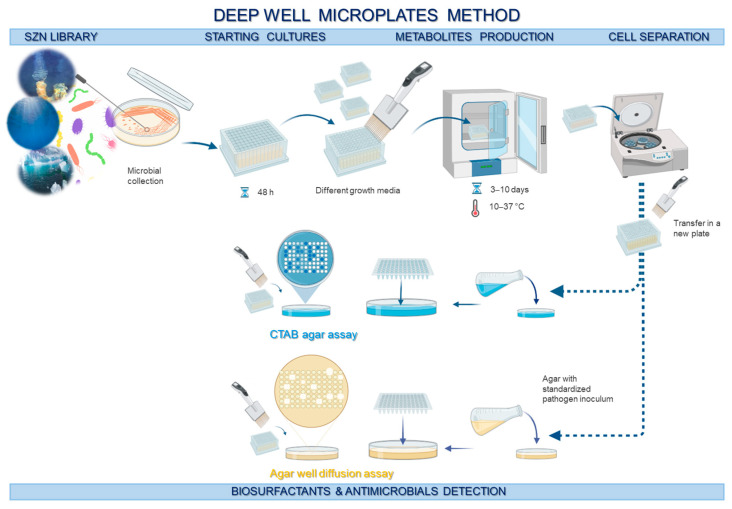
Graphical overview of the primary screening method applied to 300 marine bacteria from the Stazione Zoologica Anton Dohrn library. Bacterial cultures were grown in deep-well plates under various conditions, including different growth media, incubation periods, and temperatures. To evaluate their bioactivities, the CTAB agar assay was used to screen for biosurfactant production, while the agar diffusion well method was employed to assess antimicrobial activity.

Aiming to discover new antimicrobials and biosurfactants with membrane-disrupting activity, about 300 marine bacteria belonging to the Stazione Zoologica Anton Dohrn (SZN) library were screened. A miniaturized system was employed, setting up a micro-cultivation system for metabolite production in four steps, including (1) starting cultures, (2) metabolites production, (3) cell separation and (4) bioassay detection (as described in the Materials and Methods and shown in Figure 1). This method relies on the use of deep-well microplates, thereby allowing us to test a relatively high number of strains cultivated in different growth media to enhance the biosynthesis of a wide range of metabolites. The cetyltrimethylammonium bromide (CTAB) agar assay (followed by the oil-spreading assay for positive strains) and the agar well diffusion method [39] against *Staphylococcus aureus* 6538p and *Escherichia coli* ATCC 10536 were employed to detect the presence of biosurfactants and antimicrobials, respectively, in bacterial supernatants. Both pathogens were selected because they are the primary causes of nosocomial infections and are increasingly developing resistance to conventional antibiotics [40,41]. Among the 300 strains, particular attention was given to the strain *Bacillus* sp. BCP32 due to its significant potential as a biosurfactant producer in both the CTAB and oil spreading assays, and its notable inhibition halo against *S. aureus* 6538p. *Bacillus* sp. BCP32 is an extremophilic strain isolated from deep-sea sediments collected from Dohrn Canyon in the Gulf of Naples (Italy) [42].

### 2.2. Bioassay-Guided Fractionation of Bacillus sp. BCP32 Exhausted Broth

**Figure 2:**
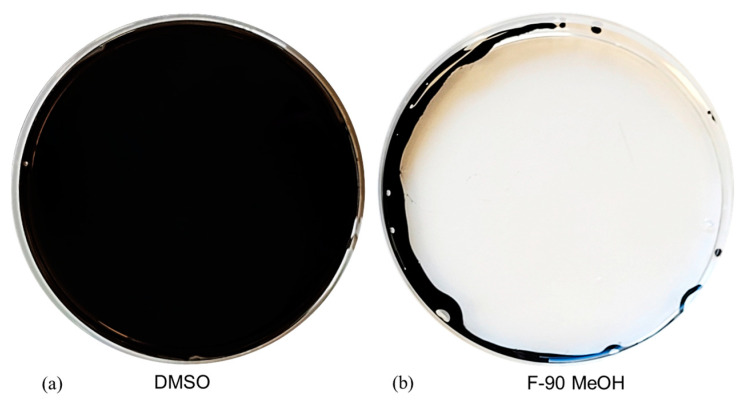
Surfactant activity of F-90% MeOH from *Bacillus* sp. BCP32 in the oil spreading test. (**a**) DMSO vehicle (4 μL) was used as negative control; (**b**) The clear zone on the oil surface indicates the presence of biosurfactants.

The selected bacterium *Bacillus* sp. BCP32 was cultivated on a small scale (50 mL in a 250 mL flask) using liquid TYP medium at 28 °C for 2 days, allowing the production of relevant metabolites to validate results from the primary screening. The exhausted broth was then extracted by ethyl acetate (EtOAc), obtaining 17 mg of crude extract, which was dissolved in DMSO (50 mg/mL) for biological assays. The oil spreading test confirmed the biosurfactant activity of *Bacillus* sp. BCP32 extract. Furthermore, the liquid inhibition assay was conducted against *S. aureus* 6538p and *E. coli* ATCC 10536, showing antibacterial activity with a MIC value of 15 μg/mL and 125 μg/mL, respectively. Based on these results, *Bacillus* sp. BCP32 was scaled-up (1L culture), and the exhausted broth was extracted by EtOAc, obtaining 430 mg of organic extract, which was fractionated by solid-phase extraction (SPE), obtaining five fractions, as described in the Section 3. Each fraction was subjected to an oil-spreading test and a liquid inhibition assay and analyzed through liquid chromatography–high-resolution tandem mass spectrometry (LC-HRMS^2^). The fraction eluted with 90% MeOH (F-90% MeOH) displayed the best bioactivity in both biosurfactant and antimicrobial assays (Table 1 and Table S1, Figure 2 and Figure S1). Concerning the antibacterial activity, the pathogens panel was expanded, including several *S. aureus* MDR strains, *S. epidermidis*, *S. xylosus*, and *Listeria monocytogenes*. The F-90% MeOH showed a MIC value ranging from 0.9 μg/mL to 3.9 μg/mL (Table 1) towards all the pathogens, except for vancomycin-resistant *S. aureus* (VRSA).

### 2.3. Molecular Networking Analysis of F-90% MeOH from Bacillus sp. BCP32

**Figure 3:**
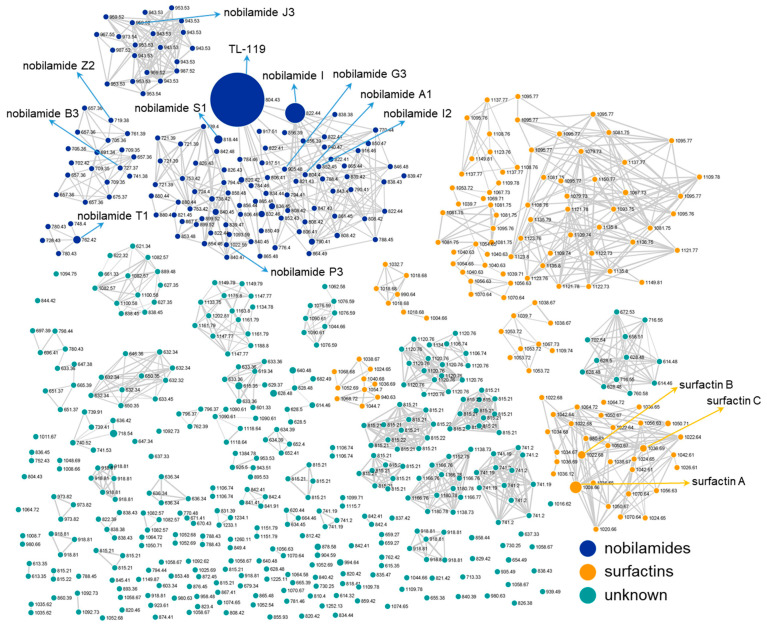
LC-HRMS^2^-based molecular network (MN) of F-90% MeOH from *Bacillus* sp. BCP32. The MN is dominated by two large clusters, i.e., nobilamides (blue nodes) and surfactins (orange nodes). Compounds are visualized as nodes, and node size reflects the compound peak area. Edge thickness is related to MS^2^ spectra similarity. Nobilamides reported in Figure 4 and compounds isolated in this study have been annotated in the MN.

**Figure 4:**
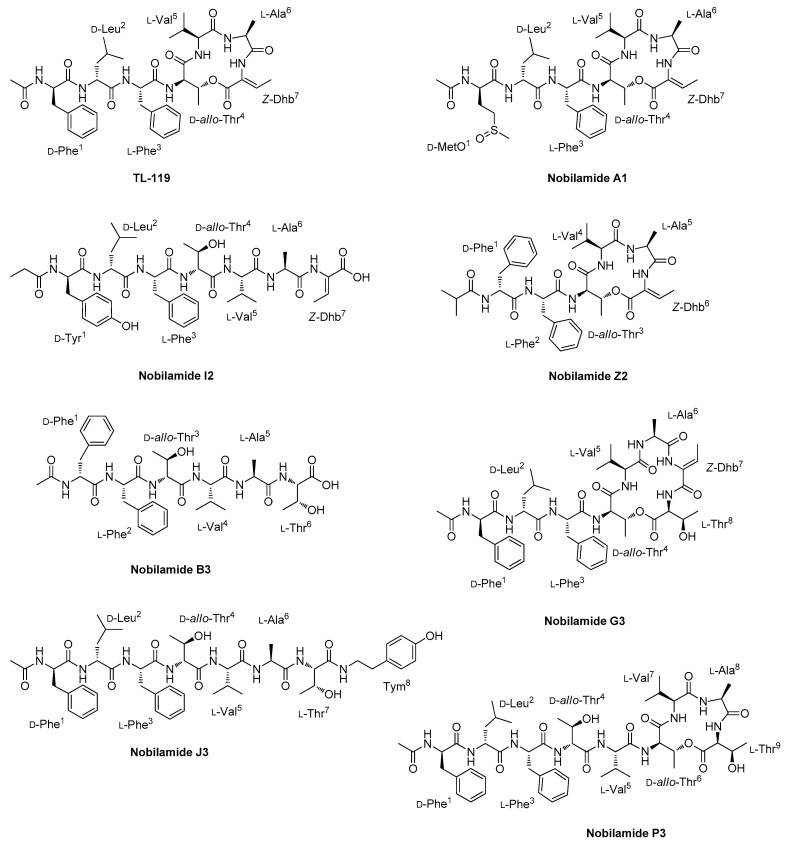
Chemical structures of representative nobilamides from *Bacillus* sp. BCP32.

Since F-90% MeOH showed both biosurfactant and antimicrobial activities, the chemical space present in this fraction was mapped by feature-based molecular networking (FBMN) analysis of untargeted MS^2^ data, as previously reported [43,44]. The FBMN workflow is available at the GNPS2 platform (https://gnps2.org, accessed on 2 October 2024), and generates an MS^2^ spectral similarity map, highlighting structurally related molecules. The molecular network of F-90% MeOH (Figure 3) unveiled the presence of two dominant molecular clusters, including nobilamides (blue nodes) (Figure 4) and surfactins (orange nodes).

While more than 100 nodes within the orange cluster could be promptly annotated as surfactins or surfactin analogues through the GNPS spectral library search, an in-depth manual dereplication of MS^2^ data was required to identify nobilamides. As the chemical diversity of surfactins has been extensively characterized so far [45,46], we focused on the structural elucidation of nobilamides, which showed to be more effective antibiotics than surfactins (see Section 2.6). The careful examination of the product ion spectra of individual compounds within the blue cluster allowed us to elucidate 84 nobilamide congeners, 71 of them being novel molecules.

The nobilamide family mainly comprises cyclic N-acyldepsiheptapeptides, where the d-*allo*-Thr^4^ side-chain OH group forms an ester bond with the C-terminal (*Z*)-2,3-dehydrobutyrine (Figure 4). Nevertheless, nobilamides may occur also as linear heptapeptides (nobilamides A-C and S-W) [37,47]. Minor congeners, including the cyclic N-acyldepsihexapeptide nobilamide D, the linear hexapeptides nobilamide C, and the linear pentapeptide nobilamide W, have been observed in *Streptomyces* and *Bacillus* spp. [37,47]. Besides the presence of cyclic N-acyldepsiheptapeptides (32) and linear heptapeptides (26), the nobilamide cluster from *Bacillus* sp. BCP32 was shown to be composed of cyclic N-acyldepsihexapeptides (11), cyclic N-acyldepsioctapeptides (2), and cyclic N-acyldepsinonapeptide (1), as well as linear hexapeptides (4) and octapeptides (8), thus unveiling that the nobilamide biosynthesis may take divergent paths to create an outstanding chemical diversity (Figure 4).

### 2.4. Structural Characterization of Nobilamide Depsiheptapeptides and Linear Heptapeptides by Mass Spectrometry and HPLC Purification

The structural characterization of the 84 nobilamide congeners was performed by LC-HRMS^2^ on a Q Exactive Focus Orbitrap mass spectrometer. The high-resolution masses of the pseudomolecular ions [M + H]^+^ included in the nobilamide network (blue nodes, Figure 3), designated the molecular formulas reported in Table 1, Table 2, Table 3, Table 4, Table 5, Table 6 and Table 7 (mass accuracy ≤ 3.0 ppm). Nobilamide precursor ions were selected to be fragmented in the higher-energy collision dissociation cell (HCD) and acquire MS^2^ data of individual compounds (Figures S5–S83).

**Figure 5:**
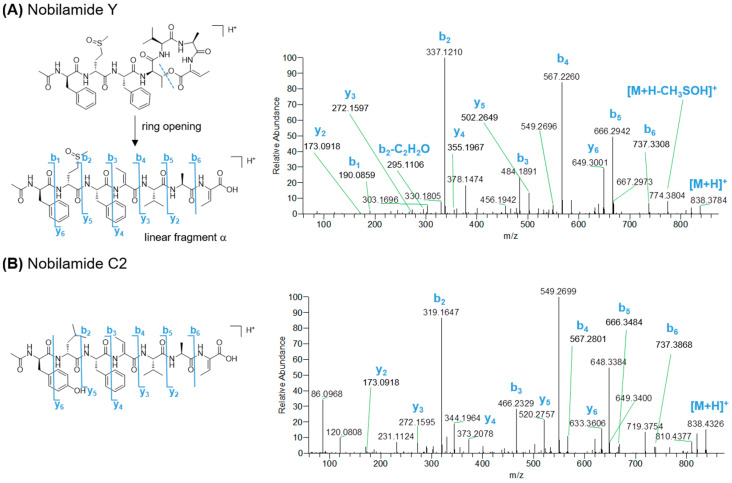
HRMS^2^ spectra of the [M + H]^+^ pseudomolecular ions of representative cyclic (**A**) and linear (**B**) nobilamides from *Bacillus* sp. BCP32.

During mass fragmentation, cyclic nobilamides first underwent ring opening at the ester bond, thus yielding the linear fragment α, where the Thr residue forming the lactone ring is converted into a dehydrobutyrine residue (Figure 5A and Figure S2). Sequential N- and C-terminal cleavages of the linear fragment generated a complete *y*- and *b*-type ion series which enabled the assignment of the amino acid sequence of nobilamides. In agreement with Iloabuchi and Spiteller [47], nobilamide lactones can be fairly distinguished from their linear counterparts as featuring a diagnostic neutral loss of the dehydrobutyrine residue (C_4_H_5_NO, 83.0371 amu) from the corresponding *b* fragment ion (e.g., *b*_4_ ion in *N*-acyldepsiheptapeptides) (Figure 5A,B). In addition, while in most cases linear nobilamides underwent a rearrangement reaction, leading to the loss of the C-terminal residue and formation of a *b_n_*_−1_ + H_2_O ion [48], nobilamide lactones gave only the *b_n_*_−1_ fragment ion, losing an intact C-terminal amino acid after the ester bond cleavage. The *b*_2_ fragment ions of *N*-acylated nobilamides displayed neutral loss of the fatty acyl chain as ketene, which was useful to infer the acyl group linked to the N-terminal amino acid (Figure 5A).

The consensus sequences of cyclic *N*-acyldepsiheptapeptides and linear heptapeptides from *Bacillus* sp. BCP32 is d-Phe^1^-d-Leu^2^-l-Phe^3^-d-*allo*-Thr^4^-l-Val^5^-l-Ala^6^-(*Z*)-2,3-dehydrobutyrine^7^ (Table 2 and Table 3) and nicely matches the specificity prediction of A domains in the nobilamide (*nbl*) synthetase (see Section 2.7).

Phe^1^ is replaced by either Leu, Met, methionine sulfoxide (MetO), or Tyr in a few congeners. When MetO is incorporated, the product ion spectra of nobilamides display fragment ions arising from the neutral loss of methanesulfenic acid (CH_3_SOH, 63.9983 Da), which is indicative of the sulfoxide group inside chain of MetO. Leu^2^ is conserved across all depsiheptapeptides and linear heptapeptides except nobilamide X and Y, which feature MetO at position 2. Met, MetO, and Leu are also found in place of Phe^3^ in some congeners; notably, nobilamide F2 bears an unprecedented putative kynurenine (Kyn) residue at position 3, as shown by the neutral loss of 190.0742 Da (C_10_H_10_N_2_O_2_) from the *y_5_* and *b_3_* ions (Figure S48). In general, by analogy with the nobilamide structures described so far, we tentatively discriminated between the isobaric Ile and Leu residues, thus assigning Leu at positions 1, 2, and 3 where present. Interestingly, this assignment was corroborated by the A domain substrate selectivity predicted in silico (see Section 2.7). Nobilamide depsiheptapeptides and linear heptapeptides from *Bacillus* sp. BCP32 share Thr^4^, with only two congeners, i.e., nobilamide L1 and L2, including Ser^4^. The most frequent amino acid at position 5 is Val, even if either Ile/Leu, Phe, Ala, *homo*-Ala, or Thr are incorporated in minor isoforms. We tentatively distinguished between the isomeric *homo*-Ala and *N*-Me-Ala in nobilamide B2 as *homo*-Ala was already reported in the structural analogue nobilamide M [47]. Ala^6^ is replaced by either Gly, Ser, or Thr, while the last amino acid is most cases represented by (*Z*)-2,3-dehydrobutyrine, which can be substituted by Thr. Notably, the presence of diaminobutyric acid (Dab) in nobilamide B1, phenethylamine (Pea) in nobilamide Q2, and tyramine (Tym) in nobilamide J2 was observed at position 7, thus unveiling unique structural variations in the nobilamide family from *Bacillus* sp. BCP32.

Most nobilamide depsiheptapeptides and linear heptapeptides undergo N-terminal acetylation. Nevertheless, isoforms having propanoyl, isobutanoyl, and C_5_H_9_O acyl groups have been identified together with deacylated derivatives.

**Figure 6:**
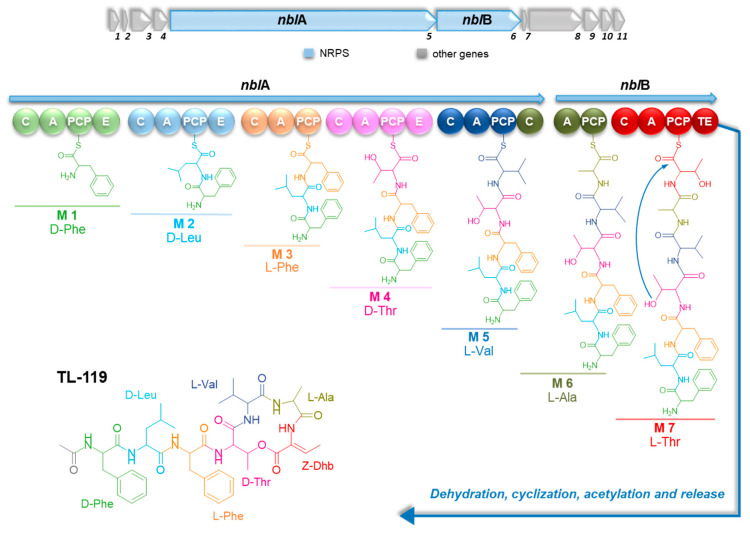
Putative biosynthesis of TL-119. Genes flanking the *nbl* operon are annotated in Table S5. Abbreviations: C, condensation domain; A, adenylation domain; PCP, peptidyl-carrier protein; E, epimerase; TE, thioesterase.

The stereochemistry of the seven amino acids was assigned through bioinformatic prediction and by analogy with the stereochemistry of nobilamides determined by Marfey’s method and NMR spectroscopy [35,47]. As the nobilamide synthetase includes three epimerization domains within modules 1, 2, and 4 (Figure 6), respectively, the presence of d-Phe^1^, d-Leu ^2^ and d-*allo*-Thr ^4^ was inferred, while the remaining amino acids were assumed to possess the l configuration. The genomics-driven absolute configuration assignment turned out to be in agreement with the reported stereochemistry for nobilamides.

The F-90% MeOH was separated by reversed phase HPLC chromatography, thus yielding the pure cyclic depsiheptapeptides TL-119 and nobilamide I, and the novel congeners T1 and S1, that were assessed for antimicrobial activity (see Section 2.6).

### 2.5. Structural Characterization of Cyclic and Linear Hexa-, Octa-, and Nonapeptides by Mass Spectrometry

Manual inspection of the nobilamide cluster within the molecular network led to the identification of cyclic N-acyldepsihexapeptides and linear hexapeptides (Table 4 and Table 5). These congeners bear the same structural features as cyclic depsiheptapeptides and linear heptapeptides, except for nobilamide A3 having an unusual C_6_H_9_O_2_ acyl group, tentatively identified as a 4-methyl-2-oxovaleryl moiety, which may either be recruited by the first NRPS module of the Nbl synthetase [49] or derive from oxidative deamination of a leucine starter unit. Cyclic nobilamides R2-U2, V2, and W2 and linear nobilamides C3 and E3 are truncated isoforms at the N- or C-terminus of the cognate heptapeptide analogues, while nobilamides X2-B3 and D3 are closely related to the already known nobilamide D, where the second amino acid, i.e., d-Leu, is missing. These findings suggest that modules 1, 2, and 7 of the Nbl synthetase might be skipped for the generation of the truncated congeners, likely as a result of an aberrant event leading to the production of minor metabolites [50]. While module skipping processes occurred in several PKSs and hybrid PKS/NRPS systems, this event has been described only once for multimodular NRPS so far, and that is the case of myxochromide S biosynthesis [51].

Differently from the other nobilamide-producing strains, *Bacillus* sp. BCP32 showed the biosynthetic ability to assemble cyclic octa- and nonadepsipeptides and linear octapeptides (Table 6, Table 7 and Table 8). Nobilamides F3 and G3 are cyclic octadepsipeptides which differ from TL-119 as having an additional amino acid at the C-terminus, i.e., l-dehydro-Asn and l-Thr, respectively, involved in the formation of the lactone ring with the side-chain OH group of d-*allo*-Thr^4^ (Figures S73 and S74). Linear octapeptides H3-O3 have the hexapeptide motif Phe^1^-d-Leu^2^-l-Phe^3^-d-*allo*-Thr^4^/Dhb^4^-l-Val^5^-l-Ala^6^, while structural diversification results in amino acid substitutions in positions 7 and 8 (Figures S75–S82). Indeed, either l-Thr, l-Thr-O-methyl ether, or (*Z*)-2,3-dehydrobutyrine is incorporated at position 7, whereas Pea, Tym, Leu/Ile, Phe, or Ala may occupy position 8. Considering that Nbl synthetase includes only seven modules, it can be argued that cyclic octadepsipeptides may derive from the iterative use of module 7, which is expected to allow for the accommodation of l-dehydro-Asn and l-Thr—structurally similar amino acids—and catalyze cyclization for the assemblage of nobilamides F3 and G3. On the other hand, linear octapeptides H3-O3 apparently derive from linear/cyclic heptapeptides, which could undergo spontaneous nucleophilic acyl substitution at the C-terminus with aromatic amines (Pea, Tym) or amino acids. It has been demonstrated that aromatic amine accumulation, due to amino acid decarboxylation, may provide building blocks for spontaneous condensation reactions, leading to the biosynthesis of imidazolium alkaloids, i.e., discolins, from *Tenacibaculum discolor* sv11 [52]. However, it cannot be excluded that a TE domain, as reported for bacillothiazols [53], or even a C-type domain present elsewhere in the chromosome, as speculated for crochelins [54], may be involved in the addition of the additional C-terminus moieties beyond the prediction of the heptamodular assembly line [55].

The peptide backbone of nobilamide P3 was determined as Phe^1^-d-Leu^2^-l-Phe^3^-d-*allo*-Thr^4^-l-Val^5^-d-*allo*-Thr^6^-l-Val^7^-l-Ala^8^-l-Thr^9^, where the d-*allo*-Thr^6^ side-chain OH group was predicted to form an ester bond with the C-terminal l-Thr^9^ (Table 8, Figure S83). Biosynthetically, nobilamide P3 may result from an aberrant process where modules 4 and 5 are used twice to generate this nonapeptide lactone, which is unusual among its congeners.

### 2.6. Surfactant and Antibacterial Activity of Nobilamides and Surfactins

Aiming to correlate surfactant and antibacterial activities to specific metabolites (Figure S3), F-90% MeOH was subjected to HPLC to obtain pure nobilamides T1, I, TL-119, and S1, as well as surfactin C and a mixture of two isomers of surfactin B, featuring identical MS^2^ spectra, which could not be resolved (major isomer 56%, minor isomer 44%) (Figure S4).

Surfactins demonstrated high biosurfactant activity in the oil spreading test (Figure S3) and antimicrobial activity against the human pathogens *S. aureus* 6538 and 6538p (MIC values ranging from 15.6 to 64.5 μg/mL) (Table S2). No activity was observed against antibiotic-resistant *S. aureus* strains, *S. epidermidis*, *S. xylosus*, and *L. monocytogenes*.

Due to the lack of antibacterial activity against the Gram-negative bacterium *E. coli* ATCC 10536, nobilamides T1, I, TL-119, and S1 were evaluated against a panel of Gram-positive pathogens. TL-119 was the most potent antibacterial agent, with MIC values ranging from 0.24 μg/mL to 7.8 μg/mL towards all tested pathogens, while no nobilamides showed activity against VRSA (Table 9). This could suggest that nobilamides may have a mechanism of action similar to that of vancomycin, which targets bacterial membranes by inhibiting cell wall synthesis [56,57].

Interestingly, even if less active as compared to TL-119, the novel nobilamide S1 maintained potent growth inhibition towards *S. aureus* 6538, *S. aureus* 6538p, and *L. monocytogenes* 677, and mild effects against *S. xilosus* MB5209 (Table 9). To the best of our knowledge, this is the first time that nobilamides demonstrated antibiotic activity towards *Listeria* and antibiotic-resistant *Staphylococcus* strains.

These findings unveiled the structural features that are essential for maintaining growth inhibitory effects against the panel of human pathogens reported in Table 9. The absence of the N-acetyl group on Phe^1^ led to the loss of bioactivity in nobilamide T1, although having the same peptide backbone as the N-acetyl encapped TL-119. In addition, Dhb^7^ is crucial to keep the antibiotic activity of TL-119, as the molecule became inactive when replaced by Thr as in nobilamide I. The Val^5^ → Leu/Ile^5^ substitution observed in nobilamide S1 markedly weakened the growth inhibitory properties of TL-119. Finally, previous results revealed the lactone ring—and the C-terminal Dhb—as a key structural motif for the antimicrobial effects of nobilamides against *S. aureus* and *L. sphaericus* [47].

Nobilamides did not show any activity in the oil spreading test, thereby indicating that only surfactins are responsible for the surfactant effects of F-90% MeOH (Figure S3).

### 2.7. Genome-Based Bacterial Identification and Elucidation of Nobilamide Biosynthetic Gene Cluster

*Bacillus* sp. BCP32 was subjected to whole genome sequencing for the correct taxonomical assignment of the species and to search for BGCs encoding the discovered compounds. DNA sequencing was performed using both Illumina short-read sequencing and Oxford Nanopore technology’s long-read sequencing, thus yielding the complete closed genome sequence. BCP32 genome has a total length of 4.197.037 bp with a G + C content of 43.76%. The analysis of its 16S rRNA gene through EzBioCloud [58,59] revealed a high similarity with *Bacillus halotolerans* ATCC 25096 (99.93%), *Bacillus mojavensis* RO-H-1 (99.86%), and *Bacillus cabrialesii* TE3 (99.80%). The BCP32-assembled genome was compared with the genomes of the above-mentioned strains using the OrthoANI Genomic Similarity tool. *Bacillus* sp. BCP32 shared the highest ANI value with *B. halotolerans* ATCC 25096 (98%), thus allowing for species assignment. The presence of *B. halotolerans* has already been reported from deep-sea and salty environments, demonstrating its capability to adapt to extreme conditions [42,60,61].

Although the first member of the nobilamide family, i.e., TL-119, was discovered in 1975 [33], there is no evidence about nobilamide biosynthesis to date. Aiming to detect the biosynthetic pathway for nobilamides, the *Bacillus* sp. BCP32 genome was mined by antiSMASH [62] for NRPS and RiPP BGCs, which are known to assemble peptide secondary metabolites. AntiSMASH enabled the detection of six NRPS and RiPP pathways, including the surfactin, bacillaene, bacillabactin, fengycin, subtilosin A, and a putative novel multimodular NRPS BGCs. Nobilamide biosynthesis was inferred to be directed by this orphan multimodular NRPS, designated as Nbl, based on the collinearity between the adenylation (A) domain specificity within each NRPS module and the chemical structure of TL-119 (see Table S3). The putative *nbl* operon is 27,723 bp long and is made up of seven NRPS modules. An in silico prediction of the A domain substrate selectivity nicely correlates with the consensus sequence d-Phe^1^-d-Leu^2^-l-Phe^3^-d-*allo*-Thr^4^-l-Val^5^-l-Ala^6^-(*Z*)-Dhb^7^, inferred by the MS-based structural characterization of cyclic depsiheptapeptides and linear heptapeptides (Section 2.4), except for Dhb^7^ (Figure 6, Table S3). Indeed, antiSMASH analysis predicted Nbl_A7 to recruit Thr, and this is likely true considering that Dhb^7^ may derive from Thr dehydration. The incorporation of dehydroamino acids, such as Dhb and Dha (α,β-dehydroalanine), in non-ribosomal peptides has been reported to involve a threonine/serine dehydration step, which can be catalyzed either by the so-called “modified AA” condensation domains (C_modAA_), such as AlbB_C_modAA_ in albopeptide biosynthesis, or by a distinct class of ^d^C_l_ domains, such as NocB_C5 in nocardicin biosynthesis [63]. The *nbl* gene cluster apparently lacks a C domain in the last module, which can presumably participate in Thr dehydration, as Nbl_C7 did not cluster with any of these unique C domains in a phylogenetic analysis performed with NaPDoS [64]. Therefore, it remains unclear whether Nbl_C7 or an auxiliary tailoring enzyme is responsible for the dehydration of Thr^7^ and how this mechanism is regulated to generate Dhb^7^ or Thr^7^–containing nobilamide isoforms. Three typical epimerization domains are present in modules 1, 2, and 4, thereby suggesting the D configuration for aa^1^, aa^2^, and aa^4^ of cyclic depsiheptapeptides and linear heptapeptides of the nobilamide family, which is consistent with the reported stereochemistry for nobilamides. The last module terminates with a canonical thioesterase (TE) domain, which is expected to catalyze the peptide release through hydrolysis or cyclization to give linear or cyclic nobilamides, respectively. Most nobilamide variants bear an acyl group at the N-terminus, which is in most cases represented by an acetyl unit. *N*-acetylated cyclic peptides are extremely rare in nature. Beyond nobilamides, chaiyaphumines from *Xenorhabdus* sp. PB61.4, heptarhizin from the endosymbiotic bacterium *Burkholderia rhizoxinica*, and griselimycins from *Streptomyces muensis* DSM 40835 are among the few *N*-acetyl encapped peptides reported to date [65,66,67]. Following a typical lipoinitiation strategy [68], starter C domains (Cs) catalyze the N-acetylation of the nonribosomal peptides griselimycins and heptarhizin. However, Nbl synthetase lacks a canonical Cs domain, although featuring a C domain in the first module, which obviously does not share significant similarity with Cs domains deposited in the NaPDoS database. Therefore, the acetylation mechanism remains elusive, raising the question of whether Nbl_C1 may acylate nobilamides at the N-terminus. In addition, acyltransferases or acyl-CoA ligases, presumably located in a different locus of the bacterial genome, could participate in nobilamide acetylation.

Interestingly, the detection of nobilamide variants containing more/less than seven amino acids clearly indicates that the Nbl synthetase may deviate from the canonical “collinearity” rule. The generation of these “odd”, minor isoforms can be traced back to aberrant biochemical processes, such as module skipping or the iterative use of specific modules, rather than programmed biosynthetic events. In addition, following amine/amino acids accumulation, spontaneous nucleophile-mediated ring opening reactions could trigger the formation of unexpected linear octapeptide congeners from cyclic heptadepsipeptides.

These processes, together with the relaxed substrate selectivity of the Nbl adenylation domains, have contributed to expand the chemical diversity of the nobilamide family.

## 3. Materials and Methods

### 3.1. Primary Screening

The SZN bacterial library was subjected to a primary screening in order to select promising strains for the production of the molecules of interest. Micro-cultivation was carried out in 96 multiwell plates. After 2 days of incubation in the most suitable medium at 20 °C under shaking conditions, 20 μL of microcultures were transferred into a new 96-deepwell (catalog number: 278743, Thermo Fisher Scientific™, Waltham, MA, USA) with different growth media. Secondary metabolite production was evaluated at a range of 3–10 days of cultivation, at temperatures between 10 and 37 °C. Afterwards, the cultures were centrifuged to separate the cells from the supernatant, which was then transferred into a new deep well plate. Particularly, 10 μL of the supernatant were used for biosurfactant assays (CTAB agar assay and oil displacement test) and 100 μL was used for the agar well diffusion assay to assess antibacterial activity.

### 3.2. Strain Identification

The strain *Bacillus* sp. BCP32 was isolated from a 450 m deep-sea sediment sample collected from the Dohrn Canyon, 19 km off the coast of the Gulf of Naples (Italy). The strain was identified as belonging to the genus *Bacillus* via 16S rRNA gene amplification and subsequent phylogenetic analysis. PCR was carried out in a total volume of 40 μL, containing 25 µL of PCR Master Mix 2× ExtraWhiteTaq (a ready-to-use solution containing TaqPol, buffer, MgCl_2_ and dNTPs), and 0.2 µM of both primers, 27F (Forward, seq: 5′-AGAGTTTGATCCTGGCTCAG-3′) and 1492R (Reverse, seq 5′-GGTTACCTTGTTACGACTT-3′) [69]. PCR was carried out under the following conditions: initial denaturation at 95 °C for 5 min, followed by 33 cycles of 95 °C for 30 s, 58 °C for 30 s, and 72 °C for 90 s, with a final extension at 72 °C for 7 min.

Afterwards PCR products were evaluated on 1% agarose gel, then purified with the GeneAll kit and sequenced by Eurofins Genomics. Finally, the phylogenetical affiliation was assigned using the EzBioCloud (http://ezbiocloud.net, accessed on 18 June 2024).

### 3.3. Bacterial Cultivation, Chemical Extraction, and Compounds Isolation

After the selection of *Bacillus* sp. BCP32 as one of the most active strains in the SZN library, a single colony of the bacterial isolate was used to inoculate 3 mL of liquid TYP medium (16 g/L bacto-tryptone, 16 g/L yeast extract, 10 g/L NaCl) in sterile bacteriological tubes. After 48 h of incubation at 20 °C at 200 rpm, the preinoculum was used to inoculate 4 × 250 mL flasks, each containing 50 mL of TYP medium, at a concentration of 0.01 OD_600_/mL. Bacterial cultures were incubated at 28 °C under shaking (150 rpm) for 48 h. Then, cultures were centrifuged at 7500 rpm at 4 °C for 30 min to collect supernatants, which were extracted with two volumes of EtOAc. Successively, organic phases were combined and evaporated with a rotary evaporator (Buchi R-100, Büchi Labortechnik AG, Postfach, Switzerland) to afford the crude extract. Finally, the obtained extract was dissolved in DMSO at 50 mg/mL and stored at 4 °C to be tested for bioactivities.

For the large-scale fermentation of *Bacillus* sp. BCP32 and subsequent compounds isolation, 4× 1 L flasks, each containing 250 mL of TYP medium, were inoculated at an initial concentration of 0.01 OD_600_/mL and incubated at 28 °C under shaking (150 rpm). After two days, the aforementioned extraction protocol was performed to obtain the crude extract from the exhausted culture broth. The EtOAc extract (430 mg) was fractionated into five fractions by solid-phase fractionation (SPE), using a reverse-phase Chromabond C-18 column Cartridge (Macherey-Nagel GmbH & Co. KG, Duren, Germany). Briefly, the extract was resuspended in the minimum volume of MeOH and uploaded onto the pre-activated SPE column. The elution was performed with two column volumes of different mixtures of MeOH and H_2_O, as follows: (1) 100% H_2_O, (2) 50% MeOH/H_2_O (*v*/*v*), (3) 90% MeOH/H_2_O (*v*/*v*), (4) 100% MeOH, and (5) MeOH + 0.1%TFA. Aliquots of the obtained fractions were dissolved in DMSO at 50 and 25 mg/mL to perform antibiotic and biosurfactant assays. The 90% MeOH fraction (F-90% MeOH) (238.2 mg) was subjected to reverse-phase HPLC separation on a semi-preparative Jupiter Proteo column (4 μm, 250 × 10 mm), thus affording nobilamide T1 (*R_t_* = 6.8 min, 1.5 mg), nobilamide I (*R_t_* = 8.6 min, 5.2 mg), TL-119 (*R_t_* = 11.1 min, 6.4 mg), nobilamide S1 (*R_t_* = 12.7 min, 1.9 mg), surfactin A (*R_t_* = 21.9 min, 4.4 mg), surfactin B (mixture of two isomers, *R_t_* = 22.7 min, 7.6 mg), and surfactin C (*R_t_* = 23.6 min, 26 mg). The column was eluted at room temperature at a flow rate of 5 mL/min with H_2_O and CH_3_CN (both eluents supplemented with 0.1% TFA), following the gradient elution programme: 0–60% CH_3_CN 0–1 min, 60−75% CH_3_CN 1–14 min, 75−85% CH_3_CN 14–15 min, 85–100% CH_3_CN 15–35 min. Detection was achieved at a 210 nm wavelength.

### 3.4. LC-HRMS^2^ Analysis of the 90% MeOH Fraction

The F-90% MeOH fraction obtained from the *Bacillus* sp. BCP32 crude extract was dissolved in MeOH at 1 mg/mL for LC-HRMS^2^ analysis, as previously described [70]. Chemical profiling was conducted using a Thermo Scientific Q Exactive Focus Orbitrap mass spectrometer coupled to a Thermo Ultimate 3000 HPLC system equipped with a Hypersil C18 column (100 × 4.6 mm, 3 μm). The column was eluted at room temperature at a flow rate of 400 μL/min with H_2_O (containing 0.1% HCOOH) and CH_3_CN, following the gradient elution programme: 25% CH_3_CN for 3 min, 25−80% CH_3_CN over 60 min, and 95% CH_3_CN for 7 min. Mass spectra were recorded in positive ion mode with a mass accuracy of ≤3 ppm. The MS parameters were as follows: spray voltage of 4.8 kV, capillary temperature of 285 °C, sheath gas flow rate of 32 units N_2_ (~150 mL/min), and auxiliary gas flow rate of 15 units N_2_. MS^2^ data were collected in data-dependent acquisition mode to fragment the three most intense ions from a full-scan mass spectrum, with an *m*/*z* range of 50 to 2000 Daltons. HRMS^2^ scans were performed by HCD fragmentation, using an isolation width of 2.0 *m*/*z*, a normalized collision energy of 15 units, and an automated injection time.

### 3.5. Feature-Based Molecular Networking

Raw files from LC-HRMS^2^ analysis of the F-90% MeOH fraction were processed by MZmine 2.53, using parameters reported in Table S4 to obtain a .mgf file. This file was employed as input on the GNPS2 Analysis Hub (https://gnps2.org, accessed on 2 October 2024) [71] to build a molecular network with the feature-based molecular networking (FBMN) workflow [72]. For FBMN analysis, the precursor ion mass tolerance was set to 0.02 Da, and the MS^2^ fragment ion tolerance was set to 0.05 Da. Subsequently, a molecular network was constructed, setting the maximum number of nodes in one cluster to 100, with edges filtered to have a cosine score above 0.7 with a minimum of four matching peaks. Analogues were also searched, not exceeding the difference of 300 Da between the query and the hit; the analogues had to follow the same clustering rules as mentioned above. Finally, the network was visualized using the Cytoscape software v. 3.8.2 and can be publicly accessed at https://gnps2.org/status?task=1b1c1180d47d47debeeea7f688d989fb, accessed on 2 October 2024.

### 3.6. Biosurfactant and Antimicrobial Assays

Biosurfactant and antimicrobial assays were performed to assess the bioactivity of crude extracts, SPE fractions, and purified compounds.

#### 3.6.1. Biosurfactants Assays

Biosurfactant-producing bacteria were screened using the cetyltrimethylammonium bromide (CTAB) agar assay [73] and the oil displacement test [74].

- CTAB Agar Assay

The CTAB agar plates method is a semi-quantitative test for the detection of anionic surfactants which was devised by Wagner and Siegmund [73]. The test is positive if the extracts contain anionic surfactants due to the formation of an insoluble ion pair between the anionic biosurfactant, CTAB, and methylene blue. Therefore, positivity is revealed by the dark blue halos around the extracts. For this assay, 1 μL of the extracts at 50 mg/mL, dissolved in DMSO were spotted on the blue agar plates. As a negative control, 4 μL of pure DMSO were used, while 4 μL of 0.1% and 0.01% sodium dodecyl sulphate (SDS) were used as positive controls. After 2 days at 4 °C, the extracts containing biosurfactants were selected by the presence of a dark blue halo around.

- Oil Displacement Test

The oil spreading assay, also called the oil displacement test [74], was performed in a Petri dish of polystyrene containing 40 μL of crude oil added to the surface of 40 mL of distilled water to form a thin oil layer. Then, 10 μL of culture supernatant or 1 μL of extracts at 50 mg/mL were gently placed on the centre of the oil layer. The diameter of the clear zone on the oil surface is related to surfactant activity, due to the presence of the biosurfactant that displaces the oil.

#### 3.6.2. Antimicrobial Assays

- Agar Well-Diffusion Method

To assess the antibacterial activity of the 300 bacteria screened, the agar well-diffusion method (ADM) was performed against *Escherichia coli* ATCC^®^ 10536™ and *Staphyloccus aureus* ATCC^®^ 6538™. This method involves inoculating an agar plate with a standardized microbial inoculum and placing 100 μL of culture supernatants into the agar through wells previously obtained, as shown in Figure 1. The sample diffuses into the medium for 24 h at 37 °C, and if the sample has activity, a zone of inhibition forms around the applied area, which is measured to assess the level of effectiveness [75].

- Liquid Inhibition Assay

To evaluate the antimicrobial potential of all samples, the MICs for *E. coli* ATCC^®^ 10536™, *S. aureus* ATCC^®^ 6538™, *S. aureus* ATCC^®^ 6538p™, methicillin-resistant *S. aureus* (MRSA), macrolide-resistant *S. aureus* (MRSA), quinolone-resistant *S. aureus* (QRSA), vancomycin-resistant *S. aureus* (VRSA) [76], *S. epidermidis* RP206 [7], *S. xylosus* MB 5209 [77] were determined by the liquid inhibition assay [78]. Briefly, the bacterial strains were inoculated in 3 mL of liquid Mueller–Hinton broth (MHB) (HiMedia, Einhausen, Germany) in sterile bacteriological tubes and incubated in agitation at 37 °C until they reached the turbidity of 0.5 McFarland. Different from the remaining pathogens, *L. monocytogenes* MB 677 [79] was diluted in the Trypticasein soy broth (TSB) medium with an additional 0.6% yeast extract. Finally, serial dilutions of the inoculum were performed to obtain a final bacterial concentration of about 5 × 10^5^ CFU/mL. Then, 4 µL of each sample was added into a 96-well microtiter plate containing 200 µL of MH medium. After being serially diluted using MH medium, 100 µL of bacterial suspension was inoculated into the broth (~5 × 10^4^ CFU/well) and incubated statically for 18 h at 37 °C. DMSO was used as a control to determine the effect of solvent on cell growth. Vancomycin, erythromycin, and ampicillin were used as positive controls. The MIC was identified as the lowest concentration that completely inhibited the growth of the pathogens, which was measured as the absorbance at 600 nm by using a ELX800 Absorbance Microplate Reader (Biotek, Winoosky, VT, USA).

### 3.7. Whole-Genome Sequencing and Bioinformatics

A total of 1 μg of genomic DNA was used for whole genome sequencing of *Bacillus* sp. BCP32, which was performed by Eurofins Genomics (Ebersberg, Germany) using the Illumina NovaSeq 6000 platform. All sequenced paired ends reads were clipped and trimmed with Trimmomatic tool [80] to remove adaptor and low-quality sequences, and a quality check was obtained with FastQC [81]. The bacterial genome was assembled with the MEGAhit algorithm [82], employing the genome of *B. halotolerans* ZB201702 (NZ_CP029364.1) as a reference to direct the arrangement and alignment of contigs. In addition, the whole genome was re-sequenced using Oxford Nanopore Technology (ONT) [83] to obtain longer reads, sending 30 μL of genomic DNA at 20 ng/mL to Eurofins Genomics. Then, to obtain a closed genome, a hybrid assembly was performed by the bioinformatic tool Unicycler [84] on the Galaxy Europe 0.5.0 server (https://usegalaxy.eu/, accessed on 12 June 2024), by inputting the raw data of both sequencing processes [85]. The assembled genome identification was carried out using the ANI Calculator by EzBioCloud [58] and the digital DNA–DNA hybridization (dDDH) analysis by Type (strain) Genome Server (https://tygs.dsmz.de, accessed on 14 June 2024). Subsequently, the assembly was analyzed using the genome mining tool antiSMASH 7.0, which led to the identification of biosynthetic gene clusters (BGCs). The nobilamide gene cluster and the whole bacterial genome of *Bacillus* sp. BCP32 were deposited in GenBank under accession numbers PQ787232 and CP176792, respectively.

## 4. Conclusions

In this study, the exploration of marine microbial biodiversity using a rapid and effective screening allowed for the identification of a deep-sea *Bacillus* of biotechnological interest. The strain was able to produce both antimicrobial and biosurfactant metabolites in the same growth conditions. Feature-based molecular networking analysis of the bacterial exometabolome shed light on the presence of two molecular families, namely surfactins and nobilamides, thereby explaining the observed bioactivities. The manual inspection of MS^2^ spectra of the nobilamide cluster enabled the structural characterization of 84 nobilamide congeners, 71 being novel molecules, thus expanding the outstanding chemical diversity of these non-ribosomal peptides. The well-known TL-119 and the novel congener nobilamide S1 were isolated as pure compounds and shown to be promising antibiotics against a range of Gram-positive bacteria, including several *S. aureus* strains, *S. xylosus*, and *L. monocytogenes*. A significant finding of this study was the potent activity of TL-119 and nobilamide S1 against the foodborne pathogen *Listeria*, which has been demonstrated for the first time, suggesting that nobilamides could be an alternative to β-lactams in the treatment of listeriosis. In addition, the novelty of this work lies in elucidating the previously unknown BGC of nobilamides. Future efforts will foresee the scale-up of *Bacillus* sp. BCP32 culture in order to purify most of the novel nobilamides identified so far and assess them for antibiotic activity.

## Figures and Tables

**Table 1:** Antibacterial activity of F-90% MeOH towards a panel of Gram-positive pathogens. ^a^ Vancomycin, ^b^ erythromycin, and ^c^ ampicillin were used as positive controls. MIC values are expressed in μg/mL.

Strain	F-90% MeOH (MIC)	Positive Control
*S. aureus* 6538p	0.9	1 ^a^
*S. aureus* 6538	0.9	0.5 ^a^
*S. aureus* methicillin-resistant	3.9	0.5 ^a^
*S. aureus* macrolide-resistant	3.9	0.5 ^a^
*S. aureus* quinolone-resistant	3.9	0.5 ^a^
*S. aureus* vancomycin-resistant	--	1 ^b^
*S. epidermidis* RP206	1.9	0.5 ^a^
*S. xilosus* MB5209	1.9	0.5 ^a^
*L. monocytogenes* 677	0.9	1 ^c^

**Table 2:** Cyclic depsiheptapeptides from *Bacillus* sp. BCP32. ^a^ The d-*allo*-Thr/Ser^4^ side-chain OH group forms an ester bond with the C-terminal residue. * Nobilamides in bold have already been reported elsewhere. Abbreviations: Ac, acetyl; Pr, propanoyl; d-*a*-Thr, d-*allo*-threonine; Dhb, 2,3-dehydrobutyrine; Dab, diaminobutyric acid.

Nobilamide *	[M + H]^+^	*m*/*z*	*R*_t_ (min)	R_1_	aa-1	aa-2	aa-3	aa-4 ^a^	aa-5	aa-6	aa-7
X	C_41_H_58_N_7_O_11_S	856.3907	14.8	Ac	d-Phe	d-MetO	l-Phe	d-*a*-Thr	l-Val	l-Ala	l-Thr
Y	C_41_H_56_N_7_O_10_S	838.3792	16.4	Ac	d-Phe	d-MetO	l-Phe	d-*a*-Thr	l-Val	l-Ala	*Z*-Dhb
Z	C_38_H_58_N_7_O_10_S	804.3950	16.8	Ac	d-Phe	d-Leu	l-MetO	d-*a*-Thr	l-Val	l-Ala	*Z*-Dhb
A1	C_38_H_58_N_7_O_10_S	804.3950	17.2	Ac	d-MetO	d-Leu	l-Phe	d-*a*-Thr	l-Val	l-Ala	*Z*-Dhb
B1	C_42_H_63_N_8_O_10_	839.4654	17.7	Ac	d-Phe	d-Leu	l-Phe	d-*a*-Thr	l-Val	l-Ala	l-Dab
**I**	**C_42_H_60_N_7_O_10_**	**822.4387**	**19.1**	**Ac**	**d-** **Phe**	**d-** **Leu**	**l-** **Phe**	**d** **-** ***a*** **Thr**	**l-** **Val**	**l-** **Ala**	**l-** **Thr**
C1	C_41_H_60_N_7_O_9_	794.4443	19.2	-	d-Phe	d-Leu	l-Phe	d-*a*-Thr	l-Leu/ Ile	l-Ala	l-Thr
**N**	**C_42_H_58_N_7_O_10_**	**820.4237**	**19.5**	**Ac**	**d-** **Phe**	**d-** **Leu**	**l-** **Phe**	**d** **-** ***a*** **Thr**	**l-** **Val**	**l-** **Ser**	***Z*** **-Dhb**
D1	C_38_H_58_N_7_O_9_S	788.4004	20.2	Ac	d-Met	d-Leu	l-Phe	d-*a*-Thr	l-Val	l-Ala	*Z*-Dhb
E1	C_41_H_58_N_7_O_10_	808.4234	20.2	Ac	d-Phe	d-Leu	l-Phe	d-*a*-Thr	l-Val	l-Gly	l-Thr
F1	C_41_H_56_N_7_O_10_	806.4087	20.3	Ac	d-Phe	d-Leu	l-Phe	d-*a*-Thr	l-Thr	l-Ala	*Z*-Dhb
G1	C_38_H_58_N_7_O_9_S	788.4009	20.5	Ac	d-Phe	d-Leu	l-Met	d-*a*-Thr	l-Val	l-Ala	*Z*-Dhb
**L**	**C_41_H_56_N_7_O_9_**	**790.4128**	**20.6**	**Ac**	**d-** **Phe**	**d-** **Leu**	**l-** **Phe**	**d** **-** ***a*** **Thr**	**l-** **Val**	**l-** **Gly**	***Z*** **-Dhb**
H1	C_42_H_58_N_7_O_10_	820.4236	20.7	Ac	d-Tyr	d-Leu	l-Phe	d-*a*-Thr	l-Val	l-Ala	*Z*-Dhb
**Q**	**C_39_H_60_N_7_O_9_**	**770.4446**	**21.2**	**Ac**	**d-** **Leu**	**d-** **Leu**	**l-** **Phe**	**d** **-** ***a*** **Thr**	**l-** **Val**	**l-** **Ala**	***Z*** **-Dhb**
I1	C_40_H_54_N_7_O_9_	776.3969	21.2	Ac	d-Phe	d-Leu	l-Phe	d-*a*-Thr	l-Ala	l-Ala	*Z*-Dhb
J1	C_43_H_60_N_7_O_10_	834.4380	20.2	Ac	d-Phe	d-Leu	l-Phe	d-*a*-Thr	l-Val	l-Thr	*Z*-Dhb
**O**	**C_39_H_60_N_7_O_9_**	**770.4446**	**21.4**	**Ac**	**d-** **Phe**	**d-** **Leu**	**l-** **Leu**	**d** **-** ***a*** **Thr**	**l-** **Val**	**l-** **Ala**	***Z*** **-Dhb**
K1	C_43_H_60_N_7_O_10_	834.4392	21.4	Pr	d-Tyr	d-Leu	l-Phe	d-*a*-Thr	l-Val	l-Ala	*Z*-Dhb
L1	C_41_H_56_N_7_O_9_	790.4133	21.7	Ac	d-Phe	d-Leu	l-Phe	d-Ser	l-Val	l-Ala	*Z*-Dhb
**TL-119** **(A-3302-B)**	**C_42_H_58_N_7_O_9_**	**804.4289**	**22.0**	**Ac**	**d-** **Phe**	**d-** **Leu**	**l-** **Phe**	**d** **-** ***a*** **Thr**	**l-** **Val**	**l-** **Ala**	***Z*** **-Dhb**
M1	C_40_H_62_N_7_O_9_	784.4608	22.6	Pr	d-Leu	d-Leu	l-Phe	d-*a*-Thr	l-Val	l-Ala	*Z*-Dhb
N1	C_40_H_62_N_7_O_9_	784.4608	22.6	Pr	d-Phe	d-Leu	l-Leu	d-*a*-Thr	l-Val	l-Ala	*Z*-Dhb
O1	C_40_H_62_N_7_O_9_	784.4608	22.6	Ac	d-Leu	d-Leu	l-Phe	d-*a*-Thr	l-Leu/ Ile	l-Ala	*Z*-Dhb
**A-3302-A**	**C_43_H_60_N_7_O_9_**	**818.4446**	**23.5**	**Pr**	**d-** **Phe**	**d-** **Leu**	**l-** **Phe**	**d** **-** ***a*** **Thr**	**l-** **Val**	**l-** **Ala**	***Z*** **-Dhb**
P1	C_39_H_54_N_7_O_8_	748.4023	24.5	-	d-Phe	d-Leu	l-Phe	d-*a*-Thr	l-Val	l-Gly	*Z*-Dhb
**J**	C_44_H_62_N_7_O_9_	832.4604	24.5	C_4_H_7_O	d-Phe	d-Leu	l-Phe	d-*a*-Thr	l-Val	l-Ala	*Z*-Dhb
Q1	C_37_H_58_N_7_O_8_	728.4339	24.6	-	d-Leu	d-Leu	l-Phe	d-*a*-Thr	l-Val	l-Ala	*Z*-Dhb
R1	C_46_H_58_N_7_O_9_	852.4289	24.8	Ac	d-Phe	d-Leu	l-Phe	d-*a*-Thr	l-Phe	l-Ala	*Z*-Dhb
S1	C_43_H_60_N_7_O_9_	818.4447	24.8	Ac	d-Phe	d-Leu	l-Phe	d-*a*-Thr	l-Leu/ Ile	l-Ala	*Z*-Dhb
**K**	**C_45_H_64_N_7_O_9_**	**846.4763**	**25.5**	**C_5_H_9_O**	**d-** **Phe**	**d-** **Leu**	**l-** **Phe**	**d** **-** ***a*** **Thr**	**l-** **Val**	**l-** **Ala**	***Z*** **-Dhb**
T1	C_40_H_56_N_7_O_8_	762.4182	28.3	-	d-Phe	d-Leu	l-Phe	d-*a*-Thr	l-Val	l-Ala	*Z*-Dhb

**Table 3:** Linear heptapeptides from *Bacillus* sp. BCP32. * Nobilamides in bold have been already reported elsewhere. Abbreviations: Ac, acetyl; Pr, propanoyl; d-*a*-Thr, d-*allo*-threonine; Dhb, 2,3-dehydrobutyrine; *h*-Ala, *homo*-alanine; Kyn, kynurenine; Pea, phenethylamine; Tym, tyramine; OMe, methyl ester.

Nobilamide *	[M + H]^+^	*m*/*z*	*R*_t_ (min)	R_1_	aa-1	aa-2	aa-3	aa-4	aa-5	aa-6	aa-7
U1	C_38_H_60_N_7_O_11_S	822.4068	15.1	Ac	d-MetO	d-Leu	l-Phe	d-*a*-Thr	l-Val	l-Ala	*Z*-Dhb
V1	C_38_H_60_N_7_O_11_S	822.4068	15.4	Ac	d-Phe	d-Leu	l-MetO	d-*a*-Thr	l-Val	l-Ala	*Z*-Dhb
W1	C_42_H_62_N_7_O_12_	856.4438	16.9	Ac	d-Phe	d-Leu	l-Tyr	d-*a*-Thr	l-Val	l-Ala	l-Thr
X1	C_41_H_60_N_7_O_11_	826.4336	17.3	Ac	d-Phe	d-Leu	l-Phe	d-*a*-Thr	l-Val	l-Gly	l-Thr
**S**	**C_42_H_62_N_7_O_11_**	**840.4491**	**17.4**	**Ac**	**d-** **Phe**	**d-** **Leu**	**l-** **Phe**	**d** **-** ***a*** **-** **Thr**	**l-** **Val**	**l-** **Ala**	**l-** **Thr**
Y1	C_40_H_56_N_7_O_10_	794.4071	17.7	Ac	d-Phe	d-Leu	l-Phe	d-*a*-Thr	l-Ala	l-Ala	*Z*-Dhb
Z1	C_38_H_60_N_7_O_10_S	806.4104	17.7	Ac	d-Met	d-Leu	l-Phe	d-*a*-Thr	l-Val	l-Ala	*Z*-Dhb
A2	C_42_H_60_N_7_O_11_	838.4339	18.1	Ac	d-Phe	d-Leu	l-Phe	d-*a*-Thr	l-Val	l-Ser	*Z*-Dhb
B2	C_41_H_58_N_7_O_10_	808.4232	18.3	Ac	d-Phe	d-Leu	l-Phe	d-*a*-Thr	l-*h*-Ala	l-Ala	*Z*-Dhb
**A**	**C_42_H_60_N_7_O_10_**	**822.4388**	**18.3**	**Ac**	**d-** **Phe**	**d-** **Leu**	**l-** **Phe**	**d** **-** ***a*** **-** **Thr**	**l-** **Val**	**l-** **Ala**	***Z*** **-** **Dhb**
C2	C_42_H_60_N_7_O_11_	838.4336	18.4	Ac	d-Tyr	d-Leu	l-Phe	d-*a*-Thr	l-Val	l-Ala	*Z*-Dhb
D2	C_43_H_64_N_7_O_11_	854.4650	18.4	Ac	d-Phe	d-Leu	l-Phe	d-*a*-Thr	l-Val	l-Ala	l-Thr- OMe
E2	C_39_H_62_N_7_O_10_	788.4544	18.6	Ac	d-Phe	d-Leu	l-Leu	d-*a*-Thr	l-Val	l-Ala	*Z*-Dhb
F2	C_43_H_61_N_8_O_11_	865.4446	18.7	Ac	d-Phe	d-Leu	l-Kyn	d-*a*-Thr	l-Val	l-Ala	*Z*-Dhb
G2	C_39_H_62_N_7_O_10_	788.4538	18.8	Ac	d-Leu	d-Leu	l-Phe	d-*a*-Thr	l-Val	l-Ala	*Z*-Dhb
H2	C_41_H_58_N_7_O_10_	808.4232	18.8	Ac	d-Phe	d-Leu	l-Phe	d-*a*-Thr	l-Val	l-Gly	*Z*-Dhb
I2	C_43_H_62_N_7_O_11_	852.4490	18.8	Pr	d-Tyr	d-Leu	l-Phe	d-*a*-Thr	l-Val	l-Ala	*Z*-Dhb
J2	C_46_H_64_N_7_O_9_	858.4751	19.4	Ac	d-Phe	d-Leu	l-Phe	d-*a*-Thr	l-Val	l-Ala	Tym
K2	C_43_H_62_N_7_O_10_	836.4543	19.5	Ac	d-Phe	d-Leu	l-Phe	d-*a*-Thr	l-Val	l-Ala	*Z*-Dhb-OMe
**B**	**C_43_H_62_N_7_O_10_**	**836.4544**	**20.0**	**Pr**	**d-** **Phe**	**d-** **Leu**	**l-** **Phe**	**d** **-** ***a*** **-** **Thr**	**l-** **Val**	**l-** **Ala**	***Z*** **-** **Dhb**
L2	C_43_H_62_N_7_O_10_	836.4544	20.0	Pr	d-Phe	d-Leu	l-Phe	d-Ser	l-Leu/ Ile	l-Ala	*Z*-Dhb
M2	C_40_H_58_N_7_O_9_	780.4287	20.4	-	d-Phe	d-Leu	l-Phe	d-*a*-Thr	l-Val	l-Ala	*Z*-Dhb
N2	C_44_H_64_N_7_O_10_	850.4705	20.8	C_4_H_7_O	d-Phe	d-Leu	l-Phe	d-*a*-Thr	l-Val	l-Ala	*Z*-Dhb
O2	C_45_H_66_N_7_O_10_	864.4863	21.6	C_5_H_9_O	d-Phe	d-Leu	l-Phe	d-*a*-Thr	l-Val	l-Ala	*Z*-Dhb
P2	C_45_H_66_N_7_O_10_	864.4863	21.6	C_4_H_7_O	d-Phe	d-Leu	l-Phe	d-*a*-Thr	l-Leu/ Ile	l-Ala	*Z*-Dhb
Q2	C_46_H_64_N_7_O_8_	842.4785	21.7	Ac	d-Phe	d-Leu	l-Phe	d-*a*-Thr	l-Val	l-Ala	Pea

**Table 4:** Cyclic depsihexapeptides from *Bacillus* sp. BCP32. * Nobilamides in bold have been already reported elsewhere. ^a^ The d-*allo*-Thr^4^ side-chain OH group forms an ester bond with the C-terminal residue. ^b^ [M + H + NH_3_]^+^. Abbreviations: Ac, acetyl; Pr, propanoyl; d-*a*-Thr, d-*allo*-threonine; Dhb, 2,3-dehydrobutyrine.

Nobilamide *	[M + H]^+^	*m*/*z*	*R*_t_ (min)	R_1_	aa-1	aa-2	aa-3	aa-4	aa-5	aa-6
R2	C_37_H_54_N_7_O_8_ ^b^	724.4009	17.4	Ac	d-Phe	d-Leu	l-Phe	d-*a*-Thr ^a^	l-Val	l-Gly
S2	C_38_H_53_N_6_O_8_	721.3908	18.2	Ac	d-Phe	d-Leu	l-Phe	d-*a*-Thr ^a^	l-Val	l-Ala
T2	C_39_H_58_N_7_O_8_ ^b^	752.4331	18.5	Pr	d-Phe	d-Leu	l-Phe	d-*a*-Thr ^a^	l-Val	l-Ala
U2	C_39_H_58_N_7_O_8_ ^b^	752.4331	18.5	Ac	d-Phe	d-Leu	l-Phe	d-*a*-Thr ^a^	l-Leu/Ile	l-Ala
**D**	**C_36_H_47_N_6_O_8_**	**691.3438**	**18.9**	**Ac**	**d-** **Phe**	**l-** **Phe**	**d-** ***a*** **-Thr ^a^**	**l-** **Val**	**l-** **Ala**	***Z*** **-Dhb**
V2	C_33_H_49_N_6_O_8_	657.3594	19.3	Ac	d-Leu	l-Phe	d-*a*-Thr ^a^	l-Val	l-Ala	*Z*-Dhb
W2	C_33_H_49_N_6_O_8_	657.3599	19.7	Ac	d-Phe	l-Leu	d-*a*-Thr ^a^	l-Val	l-Ala	*Z*-Dhb
X2	C_37_H_49_N_6_O_8_	705.3600	19.7	Pr	d-Phe	l-Phe	d-*a*-Thr ^a^	l-Val	l-Ala	*Z*-Dhb
Y2	C_37_H_49_N_6_O_8_	705.3597	19.9	Ac	d-Phe	l-Phe	d-*a*-Thr ^a^	l-Leu/Ile	l-Ala	*Z*-Dhb
Z2	C_38_H_51_N_6_O_8_	719.3756	20.8	C_4_H_7_O	d-Phe	l-Phe	d-*a*-Thr ^a^	l-Val	l-Ala	*Z*-Dhb
A3	C_40_H_53_N_6_O_9_	761.3869	23.9	C_6_H_9_O_2_	d-Phe	l-Phe	d-*a*-Thr ^a^	l-Val	l-Ala	*Z*-Dhb

**Table 5:** Linear hexapeptides from *Bacillus* sp. BCP32. Abbreviations: Ac, acetyl; d-*a*Thr, d-*allo*-threonine; Dhb, 2,3-dehydrobutyrine.

Nobilamide	[M + H]^+^	*m*/*z*	*R*_t_ (min)	R_1_	aa-1	aa-2	aa-3	aa-4	aa-5	aa-6
B3	C_36_H_51_N_6_O_10_	727.3658	15.7	Ac	d-Phe	l-Phe	d-*a*Thr	l-Val	l-Ala	l-Thr
C3	C_33_H_51_N_6_O_9_	675.3702	16.8	Ac	d-Leu	l-Phe	d-*a*Thr	l-Val	l-Ala	*Z*-Dhb
D3	C_36_H_49_N_6_O_9_	709.3544	16.9	Ac	d-Phe	l-Phe	d-*a*Thr	l-Val	l-Ala	*Z*-Dhb
E3	C_38_H_53_N_6_O_8_	721.3905	17.7	Ac	d-Phe	d-Leu	l-Phe	Dhb	l-Val	l-Ala

**Table 6:** Cyclic depsioctapeptides from *Bacillus* sp. BCP32. ^a^ The d-*allo*-Thr^4^ side-chain OH group forms an ester bond with the C-terminal residue. Abbreviations: Ac, acetyl; d-*a*Thr, d-*allo*-threonine; Dhb, 2,3-dehydrobutyrine.

Nobilamide	[M + H]^+^	*m*/*z*	*R*_t_ (min)	R_1_	aa-1	aa-2	aa-3	aa-4 ^a^	aa-5	aa-6	aa-7	aa-8
F3	C_46_H_62_N_9_O_11_	916.4557	18.6	Ac	d-Phe	d-Leu	l-Phe	d-*a*Thr	l-Val	l-Ala	*Z*-Dhb	l-dehydro-Asn
G3	C_46_H_65_N_8_O_11_	905.4762	20.8	Ac	d-Phe	d-Leu	l-Phe	d-*a*Thr	l-Val	l-Ala	*Z*-Dhb	l-Thr

**Table 7:** Linear octapeptides from *Bacillus* sp. BCP32. Abbreviations: Nob, nobilamide; Ac, acetyl; d-*a*-Thr, d-*allo*-threonine; Dhb, 2,3-dehydrobutyrine; Pea, phenethylamine; Tym, tyramine; OMe-l-Thr, l-threonine methyl ether.

Nob	[M + H]^+^	*m*/*z*	*R*_t_ (min)	R_1_	aa-1	aa-2	aa-3	aa-4	aa-5	aa-6	aa-7	aa-8
H3	C_48_H_73_N_8_O_12_	953.5333	18.1	Ac	d-Phe	d-Leu	l-Phe	d-*a*-Thr	l-Val	l-Ala	l-Thr	l-Leu/ Ile
I3	C_51_H_71_N_8_O_12_	987.5175	18.3	Ac	d-Phe	d-Leu	l-Phe	d-*a*-Thr	l-Val	l-Ala	l-Thr	l-Phe
J3	C_50_H_71_N_8_O_11_	959.5222	19.0	Ac	d-Phe	d-Leu	l-Phe	d-*a*-Thr	l-Val	l-Ala	l-Thr	Tym
K3	C_45_H_65_N_8_O_11_	893.4757	19.6	Ac	d-Phe	d-Leu	l-Phe	d-*a*-Thr	l-Val	l-Ala	*Z*-Dhb	l-Ala
L3	C_49_H_75_N_8_O_12_	967.5503	21.1	Ac	d-Phe	d-Leu	l-Phe	d-*a*-Thr	l-Val	l-Ala	OMe-l-Thr	l-Leu/ Ile
M3	C_50_H_71_N_8_O_10_	943.5279	22.6	Ac	d-Phe	d-Leu	l-Phe	d-*a*-Thr	l-Val	l-Ala	l-Thr	Pea
N3	C_51_H_73_N_8_O_11_	973.5398	26.4	Ac	d-Phe	d-Leu	l-Phe	d-*a*-Thr	l-Val	l-Ala	OMe-l-Thr	Tym
O3	C_50_H_69_N_8_O_10_	941.5132	28.0	Ac	d-Phe	d-Leu	l-Phe	Dhb	l-Val	l-Ala	l-Thr	Tym

**Table 8:** Cyclic depsinonapeptide from *Bacillus* sp. BCP32. ^a^ The d-*allo*-Thr^6^ side-chain OH group forms an ester bond with the C-terminal residue. Abbreviations: Nob, nobilamide; Ac, acetyl; d-*a*-Thr, d-*allo*-threonine.

Nob	[M + H]^+^	*m*/*z*	*R*_t_ (min)	R_1_	aa-1	aa-2	aa-3	aa-4	aa-5	aa-6 ^a^	aa-7	aa-8	aa-9
P3	C_51_H_76_N_9_O_13_	1022.5545	18.8	Ac	d-Phe	d-Leu	l-Phe	d-*a*-Thr	l-Val	d-*a*-Thr	l-Val	l-Ala	l-Thr

**Table 9:** Antibacterial activity of nobilamides from *Bacillus* sp. BCP32 towards a panel of Gram-positive pathogens. ^a^ Vancomycin, ^b^ erythromycin, and ^c^ ampicillin were used as positive controls. MIC values are expressed in μg/mL.

Strain	Nobilamide T1	Nobilamide I	Tl-119	Nobilamide S1	Positive Control
*S. aureus* 6538p	-	-	0.24	15.6	1 ^a^
*S. aureus* 6538	-	-	0.24	7.8	0.5 ^a^
*S. aureus* methicillin-resistant	-	-	3.9	-	0.5 ^a^
*S. aureus* macrolide-resistant	-	-	1.9	-	0.5 ^a^
*S. aureus* quinolone-resistant	-	-	0.9	-	0.5 ^a^
*S. aureus* vancomycin-resistant	-	-	-	-	1 ^b^
*S. epidermidis* RP206	-	-	7.8	-	0.5 ^a^
*S. xilosus* MB5209	-	-	7.8	31.5	0.5 ^a^
*L. monocytogenes* 677	-	-	0.24	3.9	1 ^c^

## Data Availability

The molecular networks and mass spectrometry data can be publicly accessed at https://gnps2.org/status?task=1b1c1180d47d47debeeea7f688d989fb, accessed on 2 October 2024. The nobilamide gene cluster and the whole genome of *Bacillus* sp. BCP32 were deposited in GenBank under accession numbers PQ787232 and CP176792, respectively.

## Supplementary Materials

The following supporting information can be downloaded at: https://www.mdpi.com/article/10.3390/md23010041/s1, Table S1: Antibacterial activity of SPE fractions from *Bacillus* sp. BCP32 crude extract towards a panel of Gram-positive pathogens; Table S2: Antibacterial activity of surfactins B (mixture of two isomers) and C; Table S3: Binding pocket signatures of adenylation domains from the Nbl synthetase; Table S4: MZmine2 parameters for MS raw data processing; Table S5: Putative genes flanking the *nbl* gene cluster; Figure S1: Surfactant activity evaluation of SPE fractions from *Bacillus* sp. BCP32 by oil spreading test; Figure S2: Ring opening of cyclic nobilamides during mass fragmentation; Figure S3: Surfactant activity evaluation of nobilamides T1, I, TL-119, and S1 and surfactins A, B, and C; Figure S4: LC-HRMS^2^ chromatograms (upper panel) and MS^2^ spectra (lower panel) of surfactin B isomers (a) and surfactin C (b); Figures S5–S83: HRMS^2^ spectra of nobilamides.
