# Supplementary material for: Novel Insights into the Nobilamide Family from a Deep-Sea Bacillus: Chemical Diversity, Biosynthesis and Antimicrobial Activity Towards Multidrug-Resistant Bacteria
Source: Mar Drugs. 2025 Jan 14;23(1):41. doi: 10.3390/md23010041 (PMC11766569; doi:10.3390/md23010041)
Supplement: Supplementary file 1 [file marinedrugs-23-00041-s001.zip › marinedrugs-3414023-supplementary.pdf]

marine drugs

Supplementary information

Novel Insights into the Nobilamide Family from a Deep-Sea *Bacillus*: Chemical Diversity, Biosynthesis and Antimicrobial Activity Towards Multidrug-Resistant Bacteria

Vincenza Casella [1,2,†], Gerardo Della Sala [1,*,†], Silvia Scarpato [1], Carmine Buonocore [1], Costanza Ragozzino [1,2], Pietro Tedesco [1], Daniela Coppola [1], Giovanni Andrea Vitale [1], Donatella de Pascale [1] and Fortunato Palma Esposito [1]

[1] Department of Ecosustainable Marine Biotechnology, Stazione Zoologica Anton Dohrn, Via A.F. Acton, 55, 80133 Naples, Italy; vincenza.casella@szn.it (V.C.); silvia.scarpato@szn.it (S.S.); carmine.buonocore@szn.it (C.B.); costanza.ragozzino@szn.it (C.R.); pietro.tedesco@szn.it (P.T.); daniela.coppola@szn.it (D.C.); giovanniandrea.vitale@unina.it (G.A.V.); donatella.depascale@szn.it (D.d.P.); fortunato.palmaesposito@szn.it (F.P.E.)
[2] Department of Chemical, Biological, Pharmaceutical and Environmental Sciences, University of Messina, Viale F. Stagno d'Alcontres, 31, 98166 Messina, Italy
* Correspondence: gerardo.dellasala@szn.it
† These authors contributed equally to this work.

Table S1. Antibacterial activity of SPE fractions from *Bacillus* sp. BCP32 crude extract towards a panel of Gram-positive pathogens.

Table S2. Antibacterial activity of surfactins B (mixture of two isomers) and C. MIC values are expressed in µg/mL.

Table S3. Binding pocket signatures of adenylation domains from the Nbl synthetase.

Table S4. MZmine2 parameters for MS raw data processing.

Table S5. Putative genes flanking the *nbl* gene cluster.

Figure S1. Surfactant activity evaluation of SPE fractions from *Bacillus* sp. BCP32 by oil spreading test.

Figure S2. Ring opening of cyclic nobilamides during mass fragmentation.

Figure S3. Surfactant activity evaluation of nobilamides T1, I, TL-119, and S1 and surfactins A, B, and C.

Figure S4. LC-HRMS^2 chromatograms (upper panel) and MS^2 spectra (lower panel) of surfactin B isomers **(a)** and surfactin C **(b).**

Figure S5. HRMS^2 spectrum of the $[M+H]^+$ pseudomolecular ion of Nobilamide X.

Figure S6. HRMS^2 spectrum of the $[M+H]^+$ pseudomolecular ion of Nobilamide Y.

Figure S7. HRMS^2 spectrum of the $[M+H]^+$ pseudomolecular ion of Nobilamide Z.

Figure S8. HRMS^2 spectrum of the $[M+H]^+$ pseudomolecular ion of Nobilamide A1.

Figure S9. HRMS^2 spectrum of the $[M+H]^+$ pseudomolecular ion of Nobilamide B1.

Figure S10. HRMS^2 spectrum of the $[M+H]^+$ pseudomolecular ion of Nobilamide I.

Figure S11. HRMS^2 spectrum of the $[M+H]^+$ pseudomolecular ion of Nobilamide C1.

Figure S12. HRMS^2 spectrum of the $[M+H]^+$ pseudomolecular ion of Nobilamide N.

Figure S13. HRMS^2 spectrum of the $[M+H]^+$ pseudomolecular ion of Nobilamide D1.

Figure S14. HRMS^2 spectrum of the $[M+H]^+$ pseudomolecular ion of Nobilamide E1.

Figure S15. HRMS^2 spectrum of the $[M+H]^+$ pseudomolecular ion of Nobilamide J1.

Figure S16. HRMS^2 spectrum of the $[M+H]^+$ pseudomolecular ion of Nobilamide F1.

Figure S17. HRMS^2 spectrum of the $[M+H]^+$ pseudomolecular ion of Nobilamide G1.

Figure S18. HRMS^2 spectrum of the $[M+H]^+$ pseudomolecular ion of Nobilamide L.

Figure S19. HRMS^2 spectrum of the $[M+H]^+$ pseudomolecular ion of Nobilamide H1.

Figure S20. HRMS^2 spectrum of the $[M+H]^+$ pseudomolecular ion of Nobilamide Q.

Figure S21. HRMS^2 spectrum of the $[M+H]^+$ pseudomolecular ion of Nobilamide I1.

Figure S22. HRMS^2 spectrum of the $[M+H]^+$ pseudomolecular ion of Nobilamide O.

Figure S23. HRMS^2 spectrum of the $[M+H]^+$ pseudomolecular ion of Nobilamide K1.

Figure S24. HRMS^2 spectrum of the $[M+H]^+$ pseudomolecular ion of Nobilamide L1.

Figure S25. HRMS^2 spectrum of the $[M+H]^+$ pseudomolecular ion of TL-119 (A-3302-B).

Figure S26. HRMS^2 spectra of the $[M+H]^+$ pseudomolecular ions of Nobilamide M1, Nobilamide N1 and Nobilamide O1.

Figure S27. HRMS^2 spectrum of the $[M+H]^+$ pseudomolecular ion of A-3302-A.

Figure S28. HRMS^2 spectrum of the $[M+H]^+$ pseudomolecular ion of Nobilamide P1.

Figure S29. HRMS^2 spectrum of the $[M+H]^+$ pseudomolecular ion of Nobilamide J.

Figure S30. HRMS^2 spectrum of the $[M+H]^+$ pseudomolecular ion of Nobilamide Q1.

Figure S31. HRMS^2 spectrum of the $[M+H]^+$ pseudomolecular ion of Nobilamide R1.

Figure S32. HRMS^2 spectrum of the $[M+H]^+$ pseudomolecular ion of Nobilamide S1.

Figure S33. HRMS^2 spectrum of the $[M+H]^+$ pseudomolecular ion of Nobilamide K.

Figure S34. HRMS^2 spectrum of the $[M+H]^+$ pseudomolecular ion of Nobilamide T1.

Figure S35. HRMS^2 spectrum of the $[M+H]^+$ pseudomolecular ion of Nobilamide U1.

Figure S36. HRMS^2 spectrum of the $[M+H]^+$ pseudomolecular ion of Nobilamide V1.

Figure S37. HRMS^2 spectrum of the $[M+H]^+$ pseudomolecular ion of Nobilamide W1.

Figure S38. HRMS^2 spectrum of the $[M+H]^+$ pseudomolecular ion of Nobilamide X1.

Figure S39. HRMS^2 spectrum of the $[M+H]^+$ pseudomolecular ion of Nobilamide S.

Figure S40. HRMS^2 spectrum of the $[M+H]^+$ pseudomolecular ion of Nobilamide Y1.

Figure S41. HRMS^2 spectrum of the $[M+H]^+$ pseudomolecular ion of Nobilamide Z1.

Figure S42. HRMS^2 spectrum of the $[M+H]^+$ pseudomolecular ion of Nobilamide A2.

Figure S43. HRMS^2 spectrum of the $[M+H]^+$ pseudomolecular ion of Nobilamide B2.

Figure S44. HRMS^2 spectrum of the $[M+H]^+$ pseudomolecular ion of Nobilamide A.

Figure S45. HRMS^2 spectrum of the $[M+H]^+$ pseudomolecular ion of Nobilamide C2.

Figure S46. HRMS^2 spectrum of the $[M+H]^+$ pseudomolecular ion of Nobilamide D2.

Figure S47. HRMS^2 spectrum of the $[M+H]^+$ pseudomolecular ion of Nobilamide E2.

Figure S48. HRMS^2 spectrum of the $[M+H]^+$ pseudomolecular ion of Nobilamide F2.

Figure S49. HRMS^2 spectrum of the $[M+H]^+$ pseudomolecular ion of Nobilamide G2.

Figure S50. HRMS^2 spectrum of the $[M+H]^+$ pseudomolecular ion of Nobilamide H2.

Figure S51. HRMS^2 spectrum of the $[M+H]^+$ pseudomolecular ion of Nobilamide I2.

Figure S52. HRMS^2 spectrum of the $[M+H]^+$ pseudomolecular ion of Nobilamide J2.

Figure S53. HRMS^2 spectrum of the $[M+H]^+$ pseudomolecular ion of Nobilamide K2.

Figure S54. HRMS^2 spectra of the $[M+H]^+$ pseudomolecular ions of Nobilamide B and Nobilamide L2.

Figure S55. HRMS^2 spectrum of the $[M+H]^+$ pseudomolecular ion of Nobilamide M2.

Figure S56. HRMS^2 spectrum of the $[M+H]^+$ pseudomolecular ion of Nobilamide N2.

Figure S57. HRMS^2 spectrum of the $[M+H]^+$ pseudomolecular ion of Nobilamide O2 and Nobilamide P2.

Figure S58. HRMS^2 spectrum of the $[M+H]^+$ pseudomolecular ion of Nobilamide Q2.

Figure S59. HRMS^2 spectrum of the $[M+NH_4]^+$ pseudomolecular ion of Nobilamide R2.

Figure S60. HRMS^2 spectrum of the $[M+H]^+$ pseudomolecular ion of Nobilamide S2.

Figure S61. HRMS^2 spectra of the $[M+NH_4]^+$ pseudomolecular ions of Nobilamide T2 and Nobilamide U2.
Figure S62. HRMS^2 spectrum of the $[M+H]^+$ pseudomolecular ion of Nobilamide D.
Figure S63. HRMS^2 spectrum of the $[M+H]^+$ pseudomolecular ion of Nobilamide V2.
Figure S64. HRMS^2 spectrum of the $[M+H]^+$ pseudomolecular ion of Nobilamide W2.
Figure S65. HRMS^2 spectrum of the $[M+H]^+$ pseudomolecular ion of Nobilamide X2.
Figure S66. HRMS^2 spectrum of the $[M+H]^+$ pseudomolecular ion of Nobilamide Y2.
Figure S67. HRMS^2 spectrum of the $[M+H]^+$ pseudomolecular ion of Nobilamide Z2.
Figure S68. HRMS^2 spectrum of the $[M+H]^+$ pseudomolecular ion of Nobilamide A3.
Figure S69. HRMS^2 spectrum of the $[M+H]^+$ pseudomolecular ion of Nobilamide B3.
Figure S70. HRMS^2 spectrum of the $[M+H]^+$ pseudomolecular ion of Nobilamide C3.
Figure S71. HRMS^2 spectrum of the $[M+H]^+$ pseudomolecular ion of Nobilamide D3.
Figure S72. HRMS^2 spectrum of the $[M+H]^+$ pseudomolecular ion of Nobilamide E3.
Figure S73. HRMS^2 spectrum of the $[M+H]^+$ pseudomolecular ion of Nobilamide F3.
Figure S74. HRMS^2 spectrum of the $[M+H]^+$ pseudomolecular ion of Nobilamide G3.
Figure S75. HRMS^2 spectrum of the $[M+H]^+$ pseudomolecular ion of Nobilamide H3.
Figure S76. HRMS^2 spectrum of the $[M+H]^+$ pseudomolecular ion of Nobilamide I3.
Figure S77. HRMS^2 spectrum of the $[M+H]^+$ pseudomolecular ion of Nobilamide J3.
Figure S78. HRMS^2 spectrum of the $[M+H]^+$ pseudomolecular ion of Nobilamide K3.
Figure S79. HRMS^2 spectrum of the $[M+H]^+$ pseudomolecular ion of Nobilamide L3.
Figure S80. HRMS^2 spectrum of the $[M+H]^+$ pseudomolecular ion of Nobilamide M3.
Figure S81. HRMS^2 spectrum of the $[M+H]^+$ pseudomolecular ion of Nobilamide N3.
Figure S82. HRMS^2 spectrum of the $[M+H]^+$ pseudomolecular ion of Nobilamide O3.
Figure S83. HRMS^2 spectrum of the $[M+H]^+$ pseudomolecular ion of Nobilamide P3.

Table S1. Antibacterial activity of SPE fractions from *Bacillus* sp. BCP32 crude extract towards a panel of Gram-positive pathogens. [a] Vancomycin, [b] erythromycin, and [c] ampicillin were used as positive controls. MIC values are expressed in μg/mL.

Strain	F-H_2O	F-50%MeOH	F-90%MeOH	F-100%MeOH	F-MeOH+0.1% TFA	Positive control
S. aureus 6538p	-	31.2	0.9	62.5	125	1[a]
S. aureus 6538	-	15.6	0.9	62.5	125	0.5[a]
S. aureus methicillin-resistant	-	-	3,9	-	-	0.5[a]
S. aureus macrolide-resistant	-	125	3.9	62.5	62.5	0.5[a]
S. aureus quinolone-resistant	-	125	3.9	62.5	-	0.5[a]
S. aureus vancomycin-resistant	-	-	-	-	-	1[b]
S. epidermidis RP206	-	62.5	1.9	125	250	0.5[a]
S. xilosus MB5209	-	62.5	1.9	125	250	0.5[a]
L. monocytogenes 677	125	15.6	0.9	31.2	62.5	1[c]

Table S2. Antibacterial activity of surfactins B (mixture of two isomers) and C. Vancomycin was used as positive control. MIC values are expressed in μg/mL.

Strain	surfactin B	surfactin C	vancomycin
S. aureus 6538p	62.5	15.6	1
S. aureus 6538	31.2	15.6	0.5

Table S3. Binding pocket signatures of adenylation domains from the Nbl synthetase.

antiSMASH		Binding pocket signatures (position)[a]									
Prediction		235	236	239	278	299	301	322	330	331	517
**Nbl_A1**	**Phe**	D	A	W	T	I	A	A	I	C	K
**Nbl_A2**	**Leu**	D	A	W	L	I	G	A	I	I	K
**Nbl_A3**	**Phe**	D	A	W	T	I	A	A	I	C	K
**Nbl_A4**	**Thr**	D	F	W	N	I	G	M	V	H	K
**Nbl_A5**	**Val**	D	A	F	W	I	G	G	T	F	K
**Nbl_A6**	**Ala**	D	V	T	N	F	A	I	I	Y	K
**Nbl_A7**	**Thr**	D	F	W	N	I	G	M	V	H	K

[a] as reported by Della Sala et al. [1].

Table S4. MZmine2 parameters for MS raw data processing.

**Feature detection**	
MS level 1	1.0e5
MS level 2	1.0e3
ADAP chromatogram builder	• group intensity threshold: 1.0e5<br>• minimum highest intensity: 1.0e5<br>• *m/z* tolerance: 0.01 *m/z* or 10 ppm
**Chromatogram deconvolution**	
local minimum search	• chromatographic threshold: 10%<br>• search minimum in RT range: 0.50 min<br>• minimum relative height: 30%<br>• minimum absolute height: 1.0e5<br>• minimum ratio of peak top/edge: 1.2<br>• peak duration: 0-10 min<br>• m/z range for MS2 scan pairing: 0.01 Da<br>• RT range for MS2 scan pairing: 0.2 min
**Alignment**	
Join aligner	• *m/z* tolerance: 0.002 *m/z* or 5 ppm<br>• retention time tolerance: 0.2 min
**Identification**	
Adduct search	• retention time tolerance: 0.1 min

([M+Na–H], [M+K–H], [M+Mg–2H], [M+NH_3], [M-Na+NH_4], [M+1, ^{13}C])	• *m/z* tolerance: 0.002 *m/z* or 5 ppm<br>• max relative adduct peak height: 100%

Table S5. Putative genes flanking the *nbl* gene cluster.

protein	amino acids	closest homolog (protein, origin)	identities/ positives (%)	accession #
ORF1	197	DUF881 domain-containing protein [*Bacillus halotolerans*]	100/100	WP_202852504.1
ORF2	121	small basic family protein [*Bacillus*]	100/100	WP_010334164.1
ORF3	440	cell division protein FtsA [*Bacillus*]	100/100	WP_024121323.1
ORF4	382	cell division protein FtsZ [*Bacillus*]	98/98	WP_095843675.1
*nbl*A	7068	non-ribosomal peptide synthase [*Bacillus subtilis*]	98/99	WP_160243239.1
*nbl*B	2116	non-ribosomal peptide synthase [*Bacillus subtilis*]	99/99	WP_240304268.1
ORF7	91	cell division protein FtsZ [*Bacillus spizizenii*]	99/98	MCY8131166.1
ORF8	1434	S8 family peptidase [*Bacillus halotolerans*]	97/99	WP_227599196.1
ORF9	317	Sporulation sigma-E factor processing peptidase (SpoIIGA) [*Bacillus mojavensis*]	99/99	QJC96072.1
ORF10	239	RNA polymerase sporulation sigma factor SigE [*Bacillus*]	100/100	WP_024121327.1
ORF11	260	RNA polymerase sporulation sigma factor SigG [*Bacillus*]	100/100	WP_010334169.1

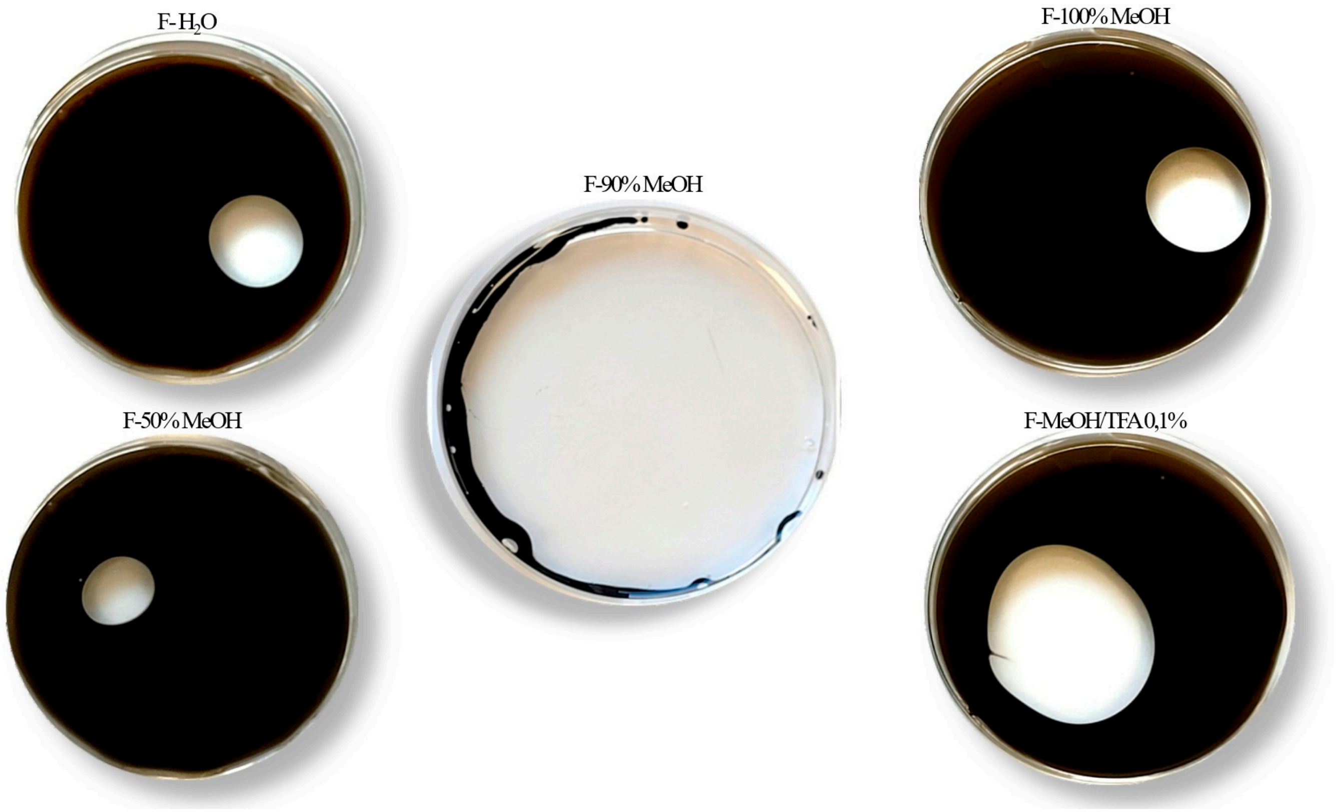

Figure S1. Surfactant activity evaluation of SPE fractions from *Bacillus* sp. BCP32 by oil spreading test. DMSO (4 μL) was used as negative control. The clear zone on the oil surface indicates the presence of biosurfactants. F-90% MeOH showed the best surfactant effect.

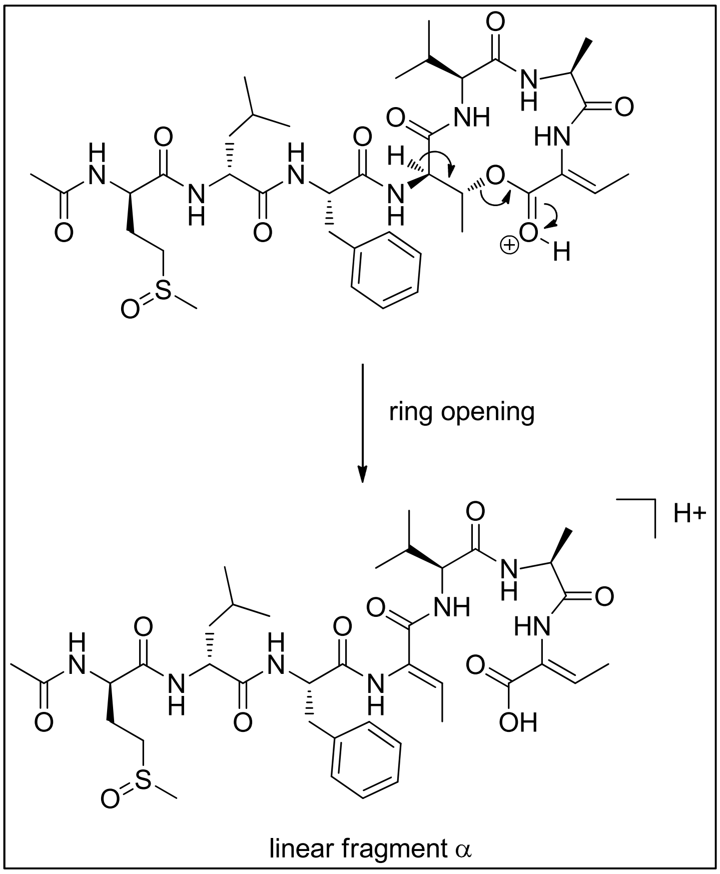

Figure S2. Ring opening of cyclic nobilamides during mass fragmentation. The cleavage of the ester bond generates the linear fragment α, where the Thr residue forming the lactone ring is converted into a dehydrobutyrine residue.

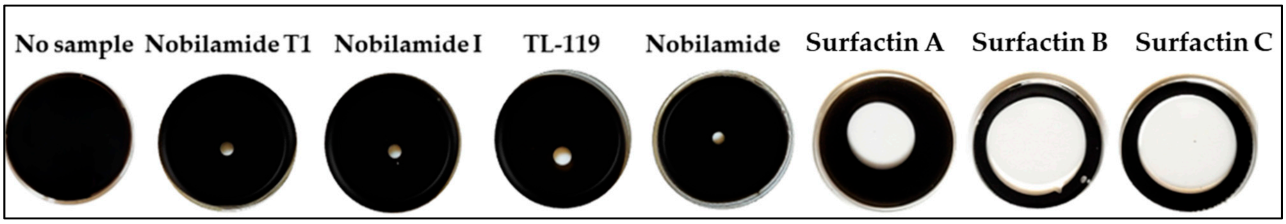

Figure S3. Surfactant activity evaluation of nobilamides T1, I, TL-119, and S1 and surfactins A, B, and C. DMSO (4 μL) was used as negative control. Surfactins displaced oil and formed a clear zone in the oil layer, thus indicating potent surfactant activity.

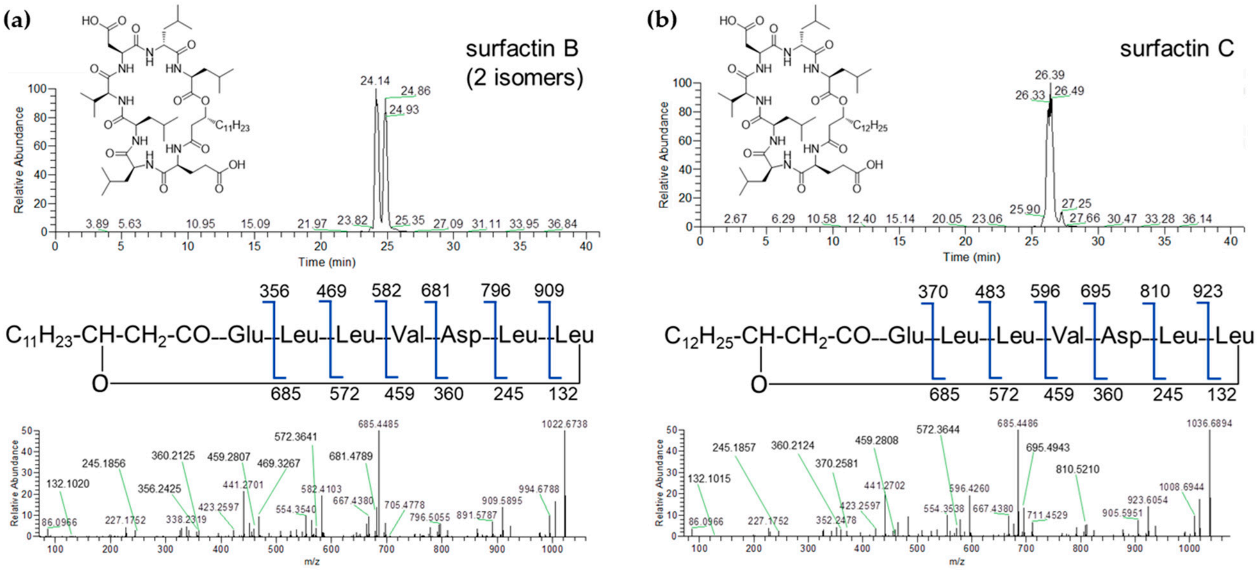

Figure S4. LC-HRMS^2 chromatograms (upper panel) and MS^2 spectra (lower panel) of surfactin B isomers **(a)** and surfactin C **(b).**

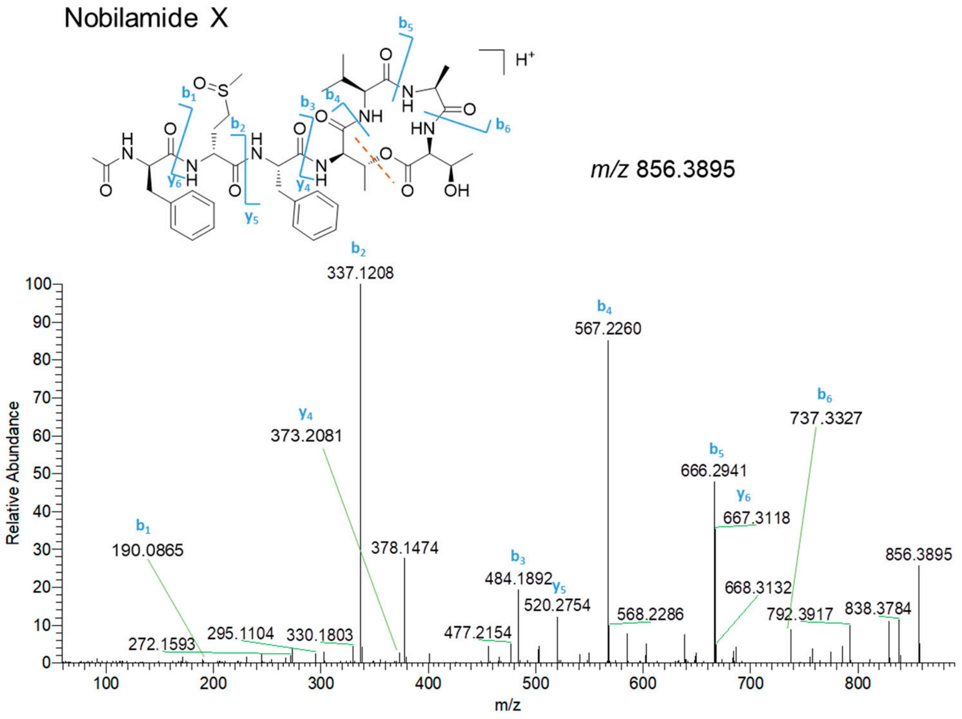

Figure S5. HRMS^2 spectrum of the [M+H]$^+$ pseudomolecular ion of Nobilamide X.

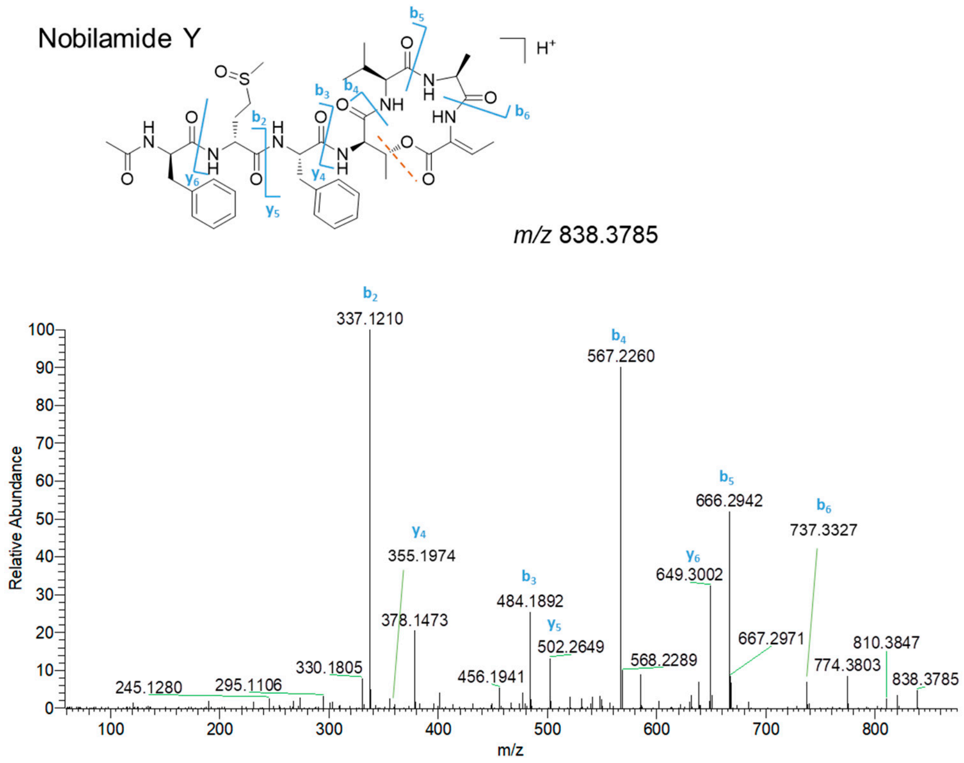

Figure S6. HRMS[2] spectrum of the [M+H]+ pseudomolecular ion of Nobilamide Y.

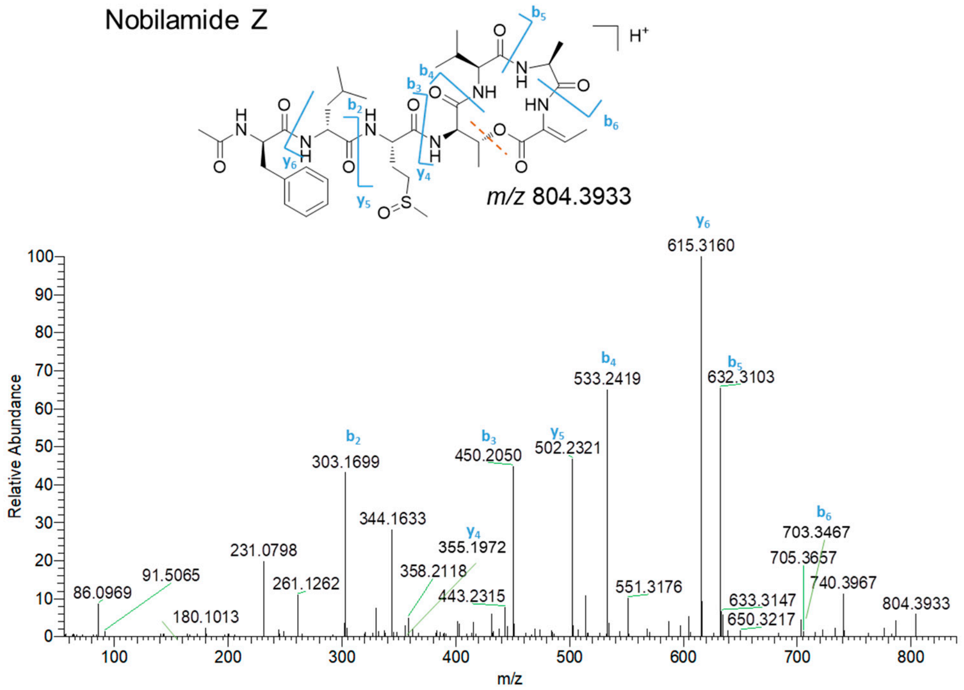

Figure S7. HRMS[2] spectrum of the [M+H]+ pseudomolecular ion of Nobilamide Z.

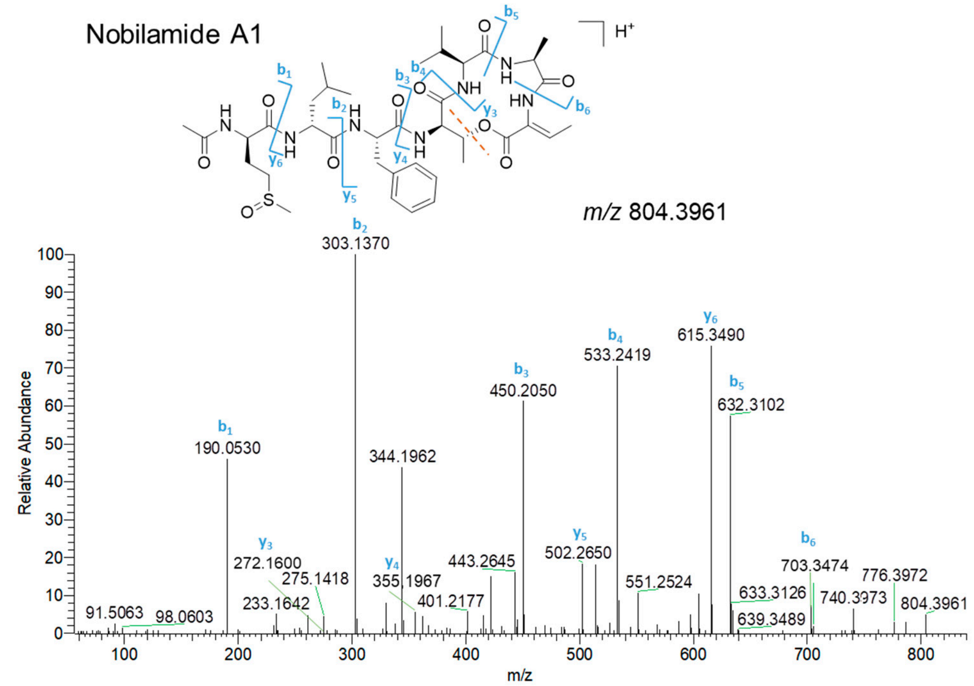

Figure S8. HRMS² spectrum of the [M+H]⁺ pseudomolecular ion of Nobilamide A1.

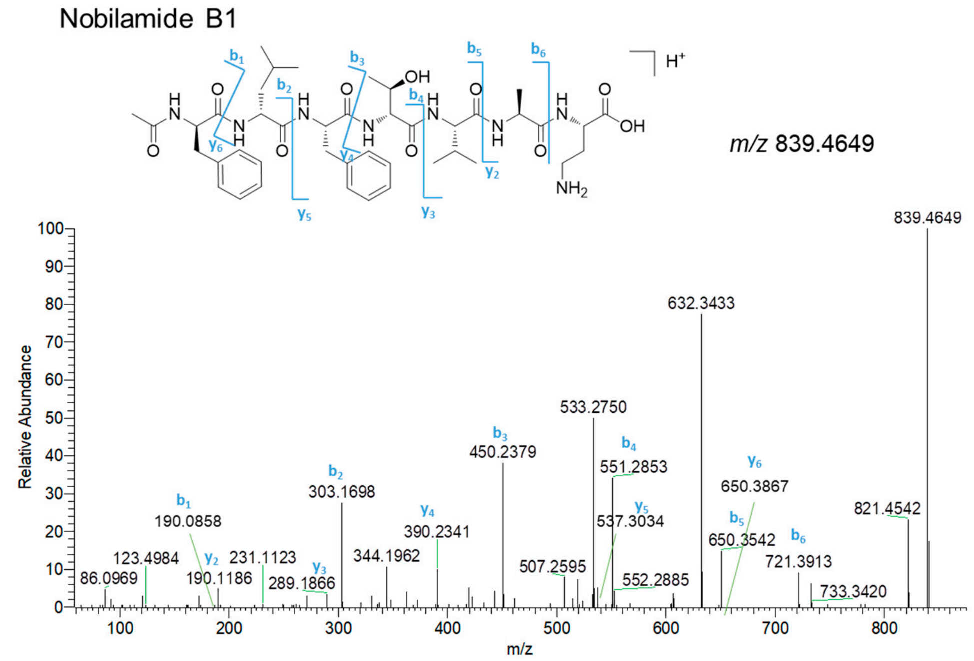

Figure S9. HRMS² spectrum of the [M+H]⁺ pseudomolecular ion of Nobilamide B1.

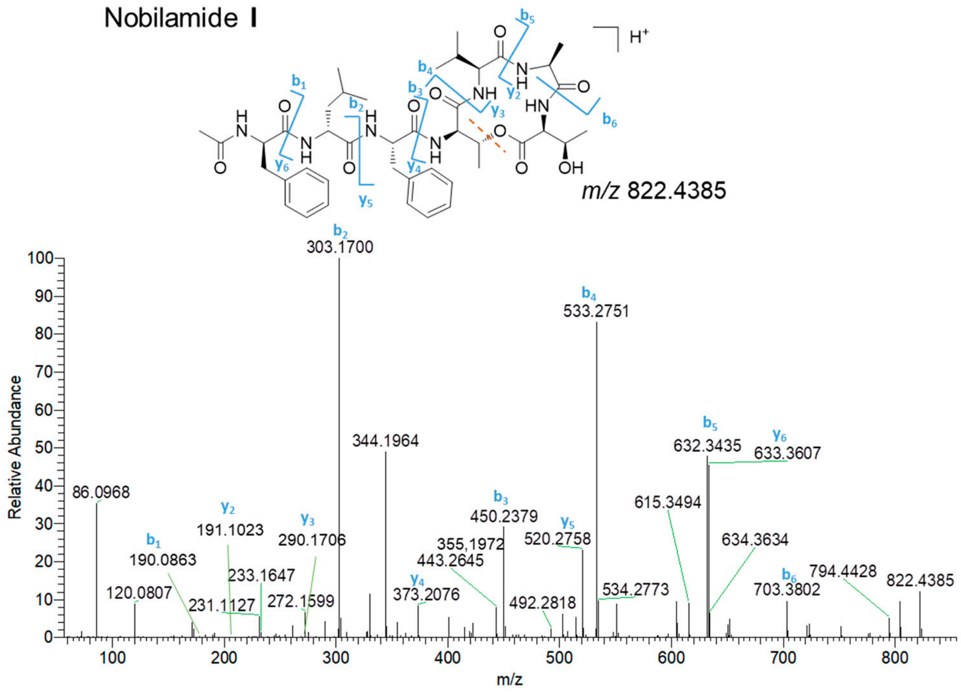

Figure S10. HRMS² spectrum of the [M+H]⁺ pseudomolecular ion of Nobilamide I.

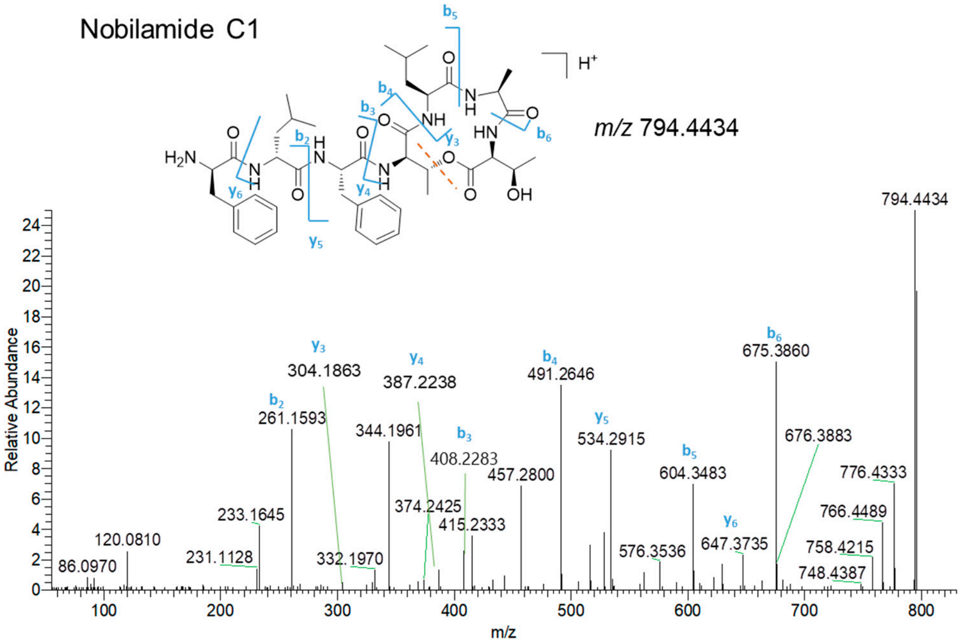

Figure S11. HRMS² spectrum of the [M+H]⁺ pseudomolecular ion of Nobilamide C1.

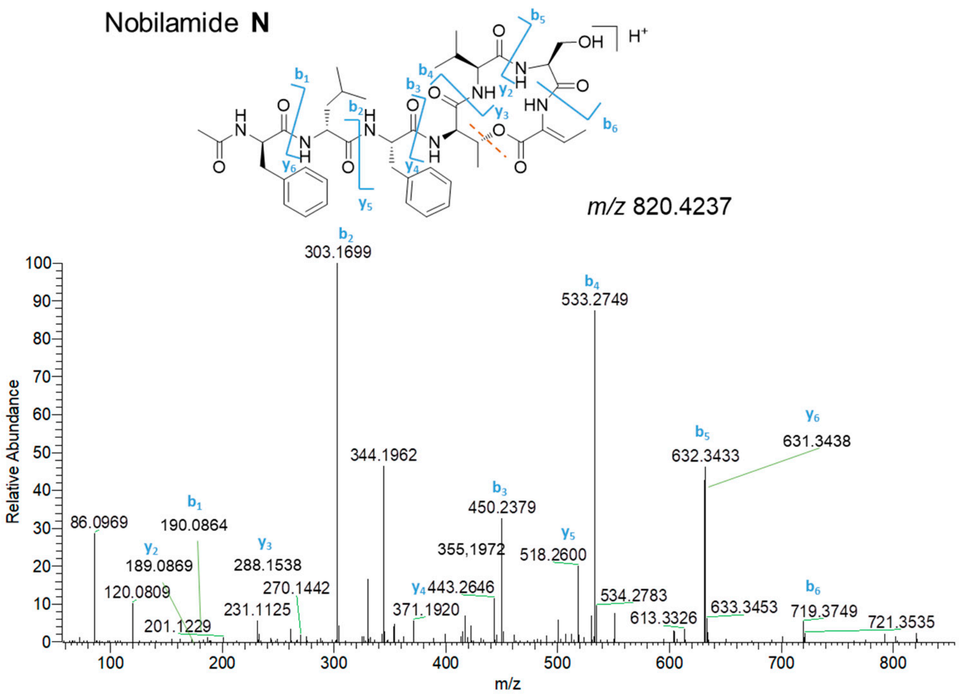

Figure S12. HRMS² spectrum of the [M+H]⁺ pseudomolecular ion of Nobilamide N.

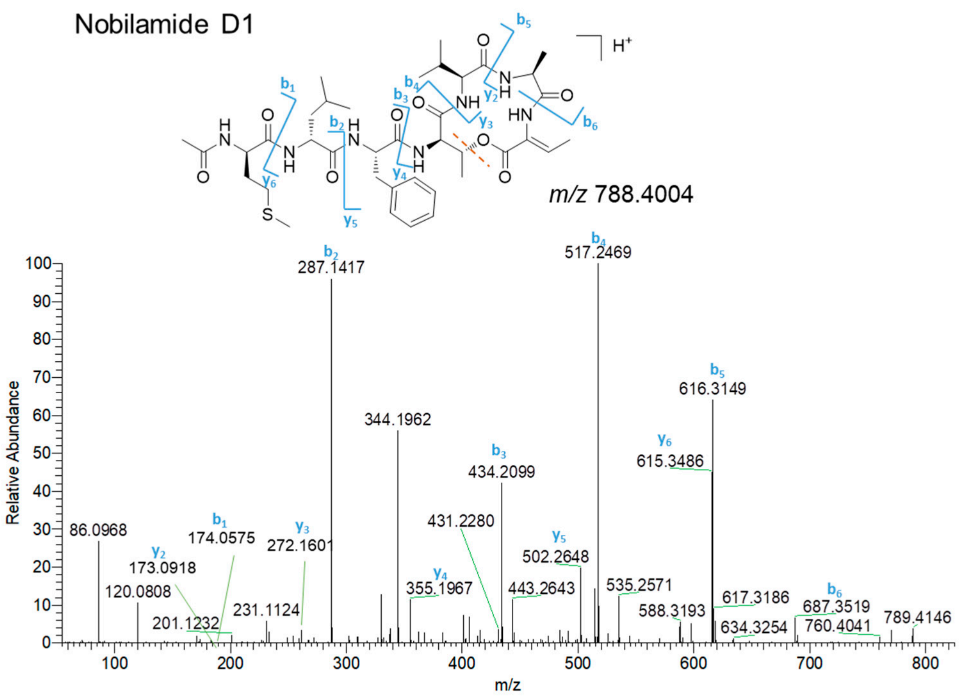

Figure S13. HRMS² spectrum of the [M+H]⁺ pseudomolecular ion of Nobilamide D1.

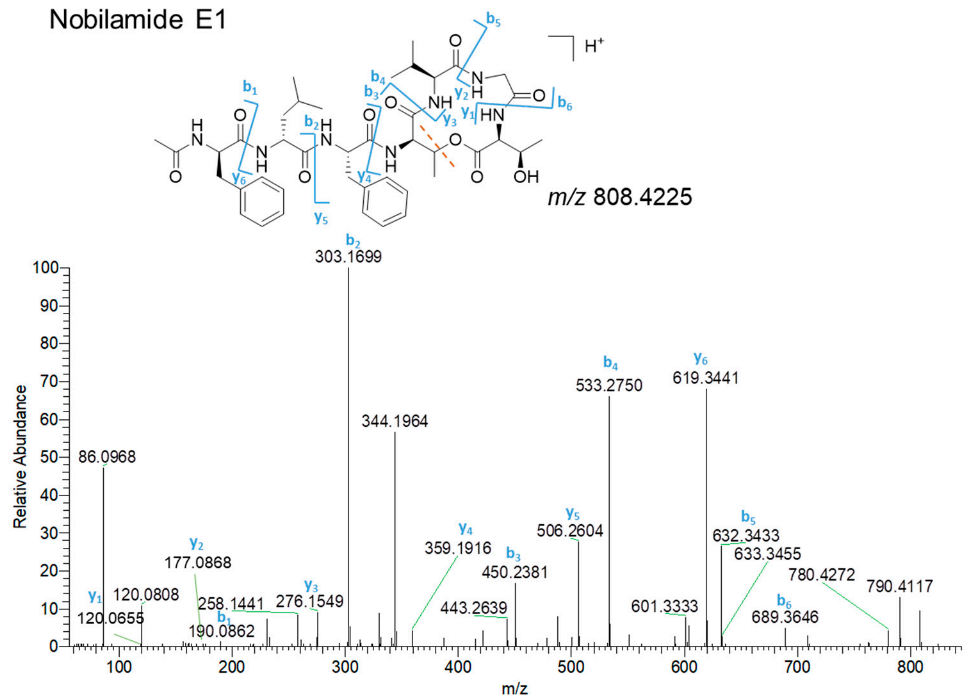

Figure S14. HRMS² spectrum of the [M+H]⁺ pseudomolecular ion of Nobilamide E1.

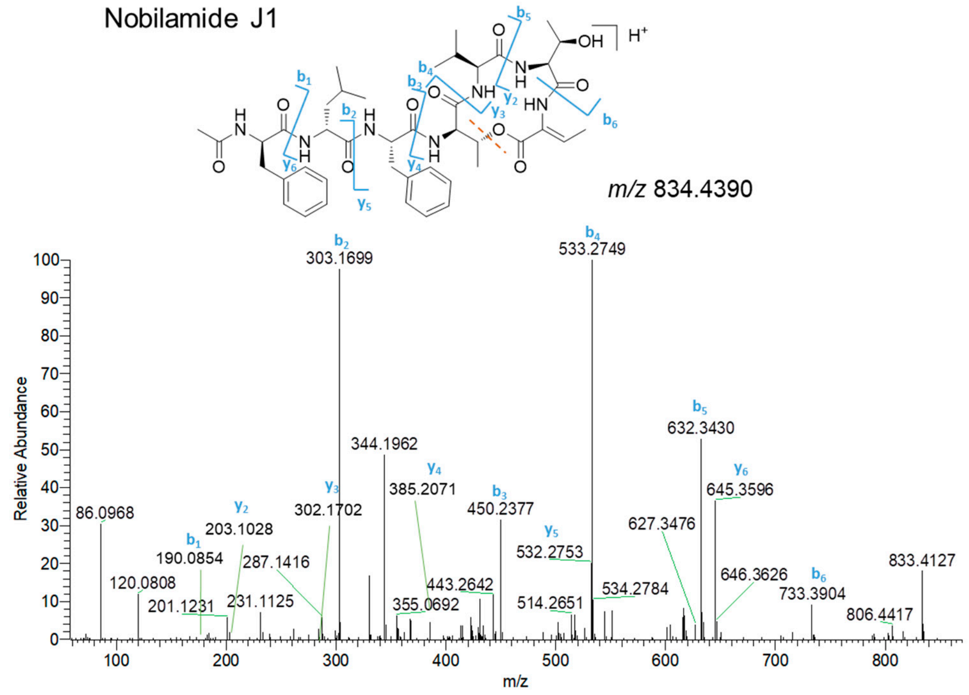

Figure S15. HRMS² spectrum of the [M+H]⁺ pseudomolecular ion of Nobilamide J1.

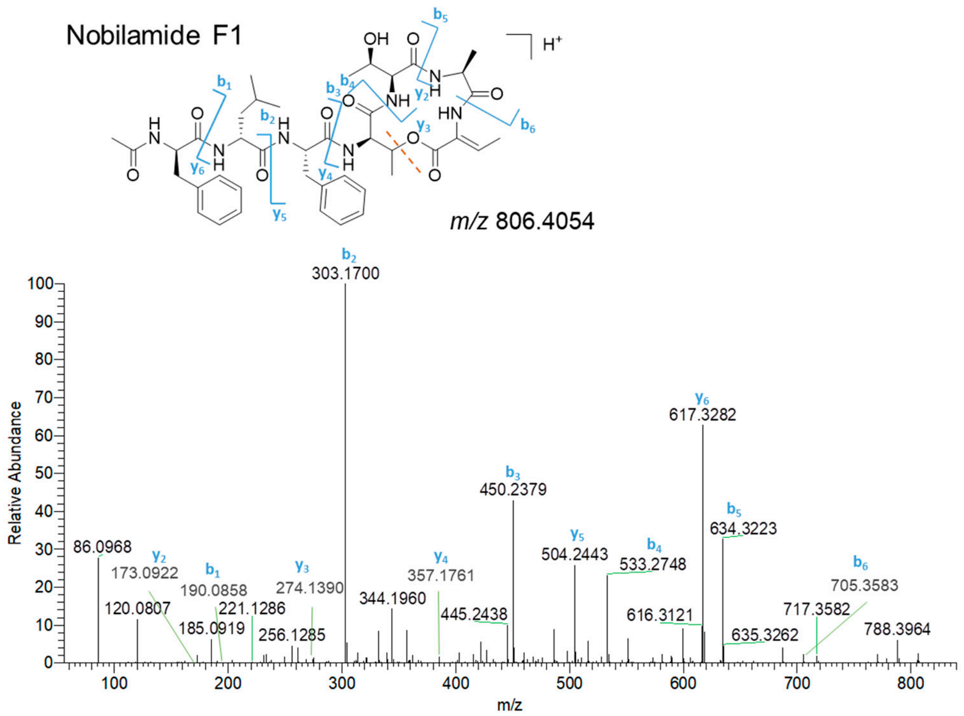

Figure S16. HRMS² spectrum of the [M+H]⁺ pseudomolecular ion of Nobilamide F1.

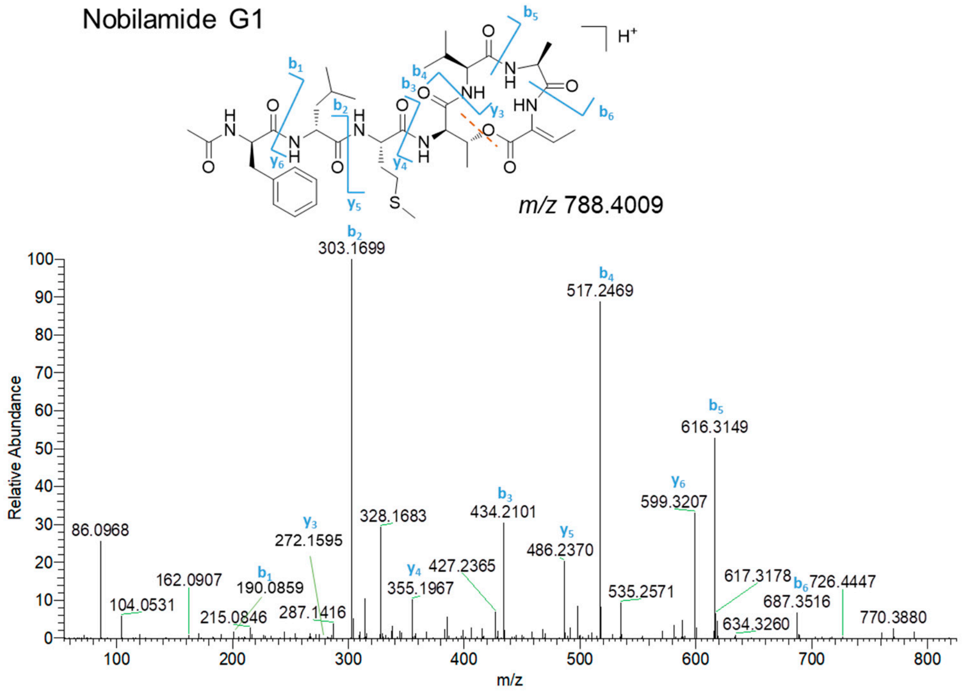

Figure S17. HRMS² spectrum of the [M+H]⁺ pseudomolecular ion of Nobilamide G1.

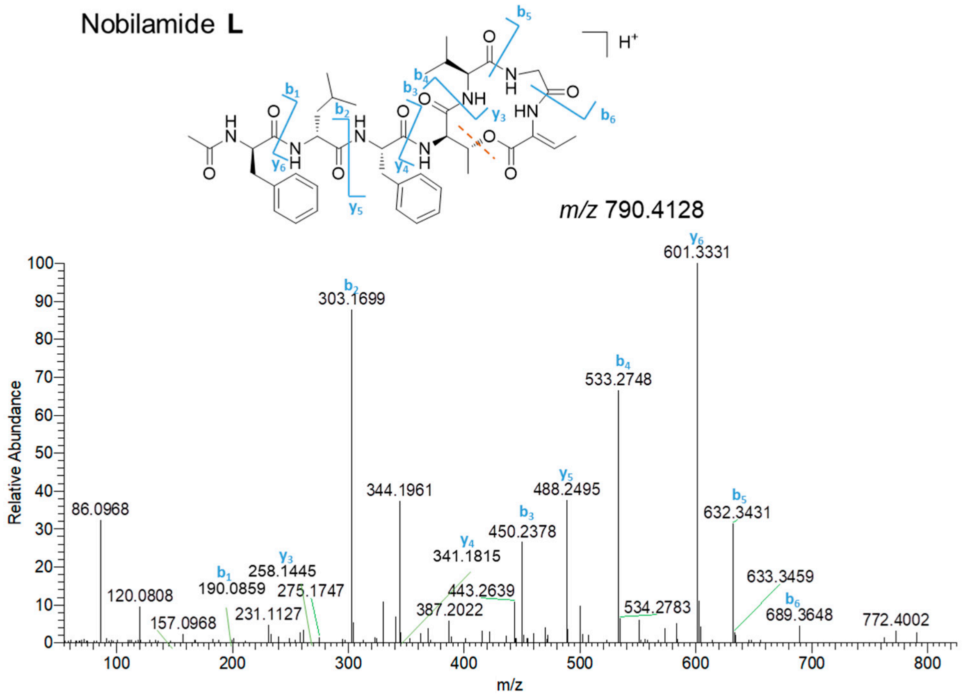

Figure S18. HRMS² spectrum of the [M+H]⁺ pseudomolecular ion of Nobilamide L.

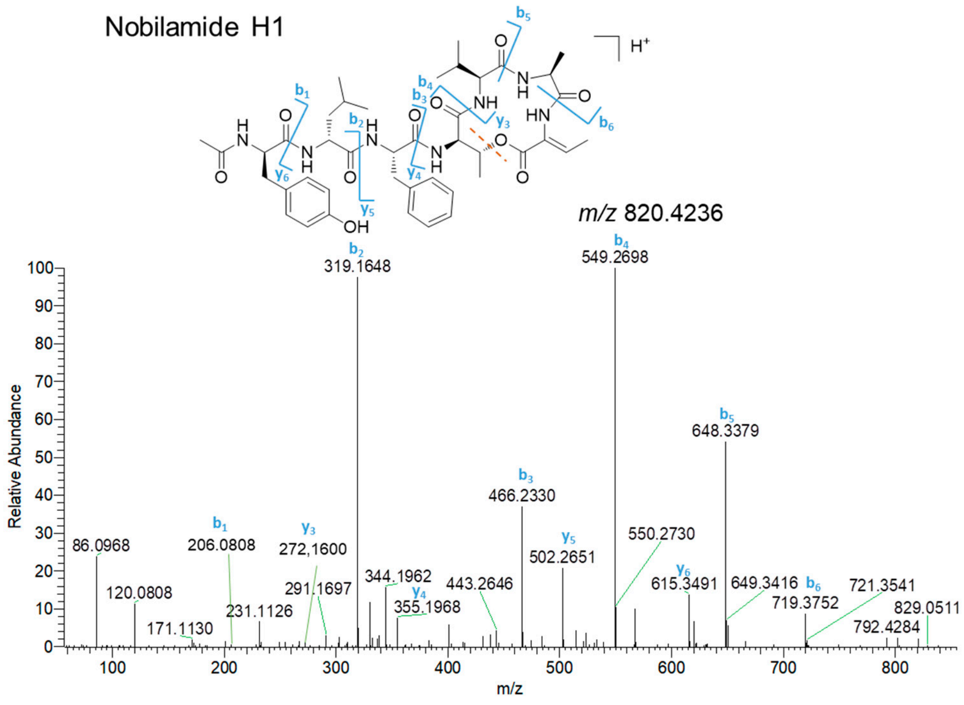

Figure S19. HRMS² spectrum of the [M+H]⁺ pseudomolecular ion of Nobilamide H1.

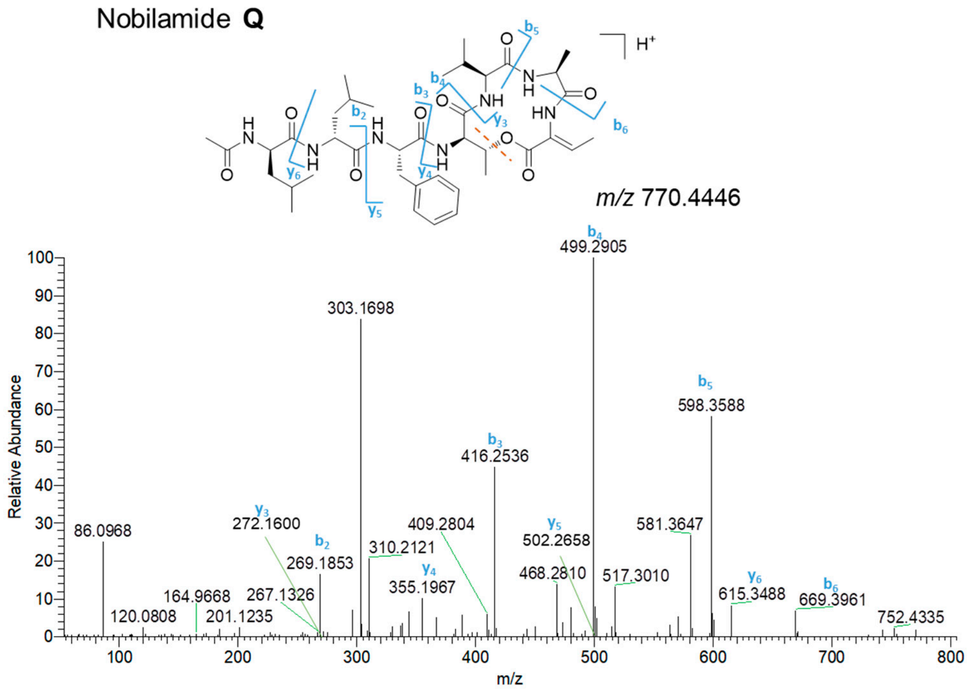

Figure S20. HRMS[2] spectrum of the $[M+H]^+$ pseudomolecular ion of Nobilamide Q.

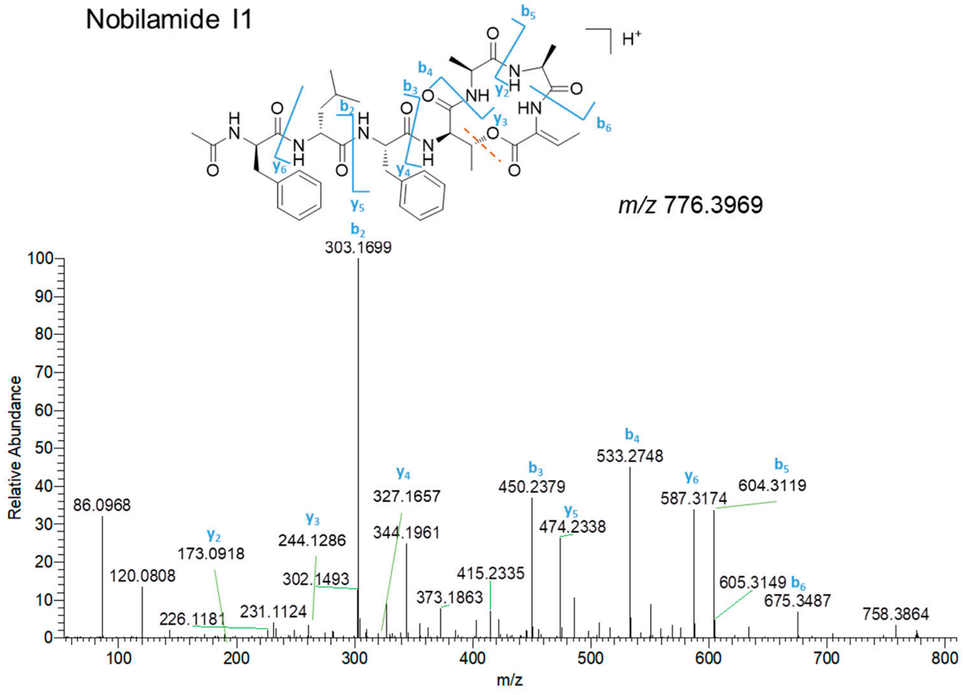

Figure S21. HRMS[2] spectrum of the $[M+H]^+$ pseudomolecular ion of Nobilamide I1.

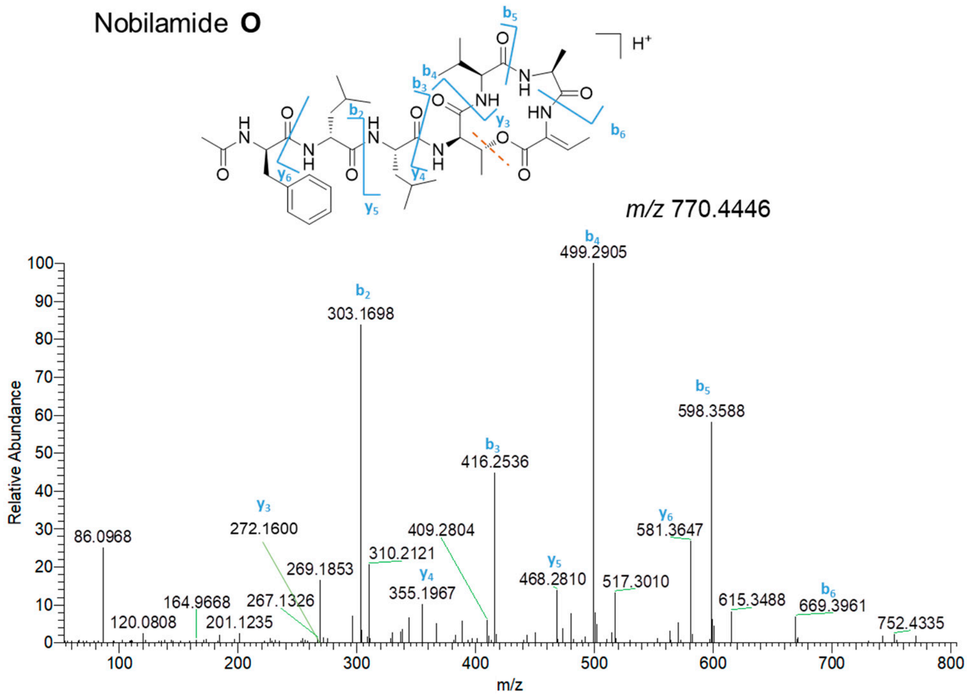

Figure S22. HRMS² spectrum of the [M+H]⁺ pseudomolecular ion of Nobilamide O.

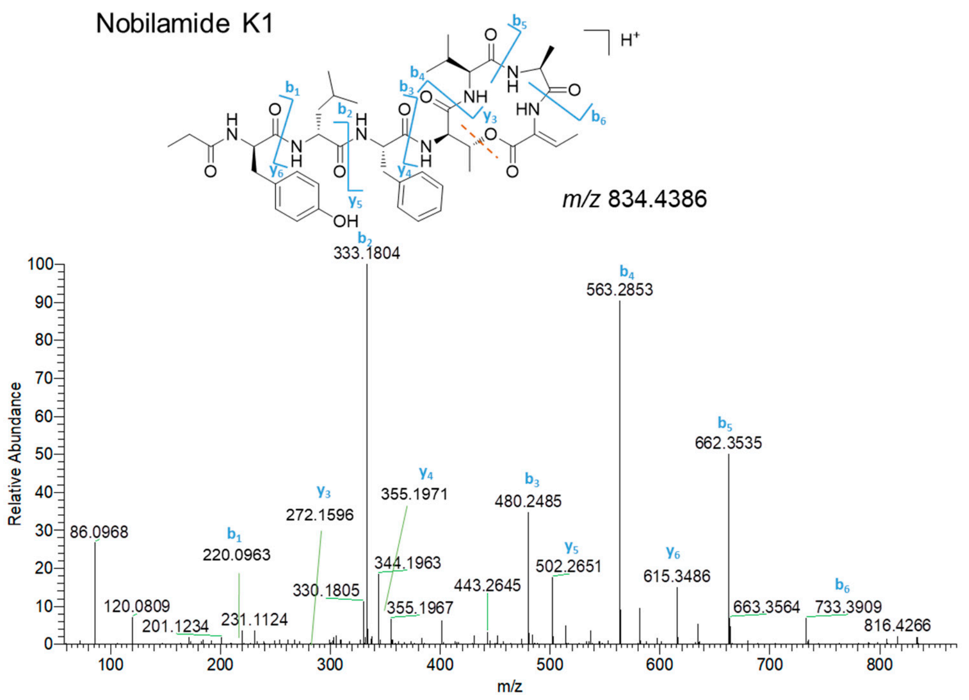

Figure S23. HRMS² spectrum of the [M+H]⁺ pseudomolecular ion of Nobilamide K1.

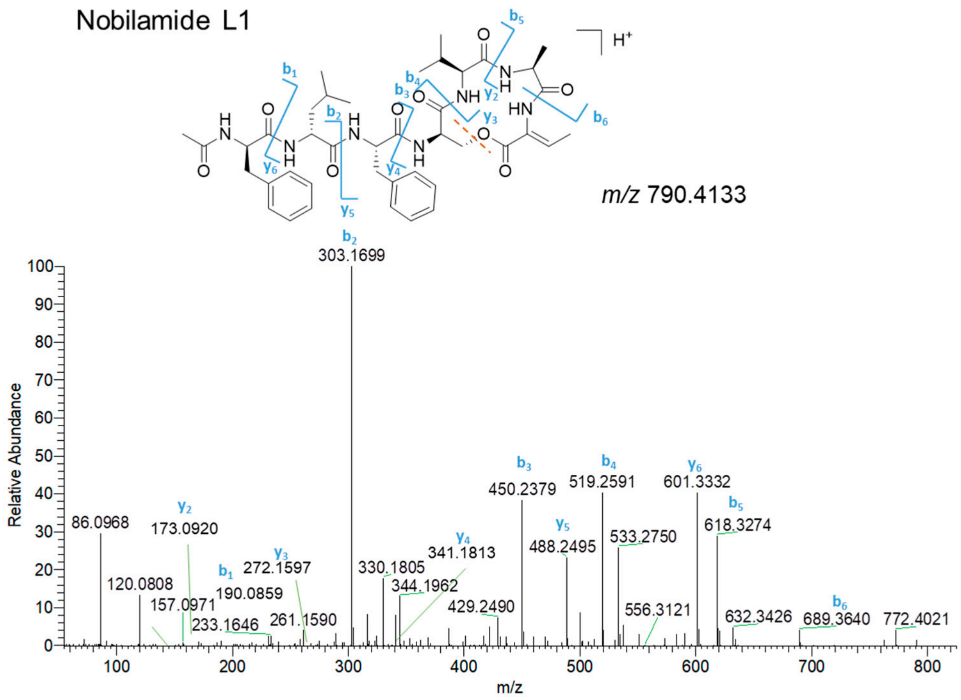

Figure S24. HRMS² spectrum of the [M+H]⁺ pseudomolecular ion of Nobilamide L1.

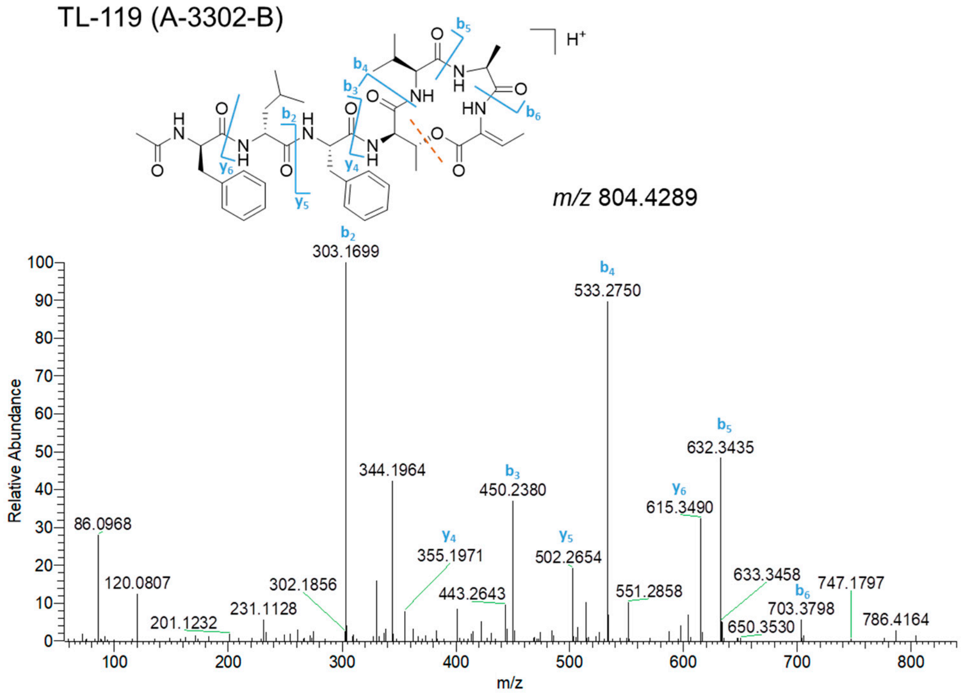

Figure S25. HRMS² spectrum of the [M+H]⁺ pseudomolecular ion of TL-119 (A-3302-B).

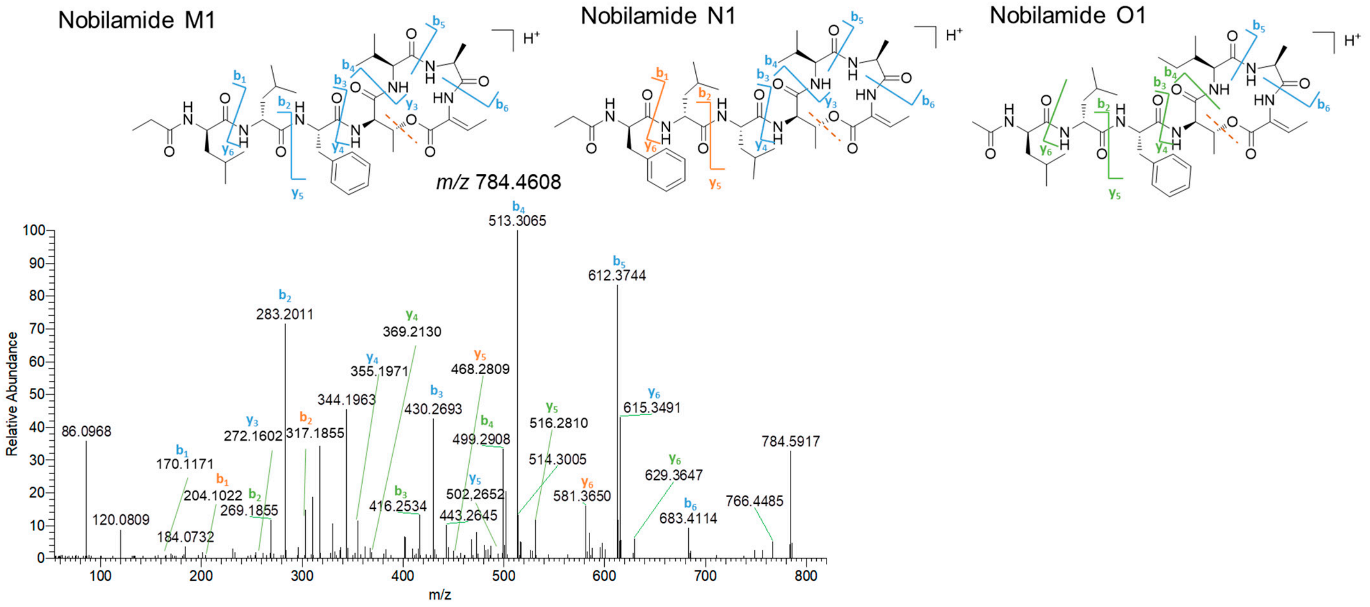

Figure S26. HRMS² spectra of the [M+H]⁺ pseudomolecular ions of Nobilamide M1, Nobilamide N1 and Nobilamide O1.

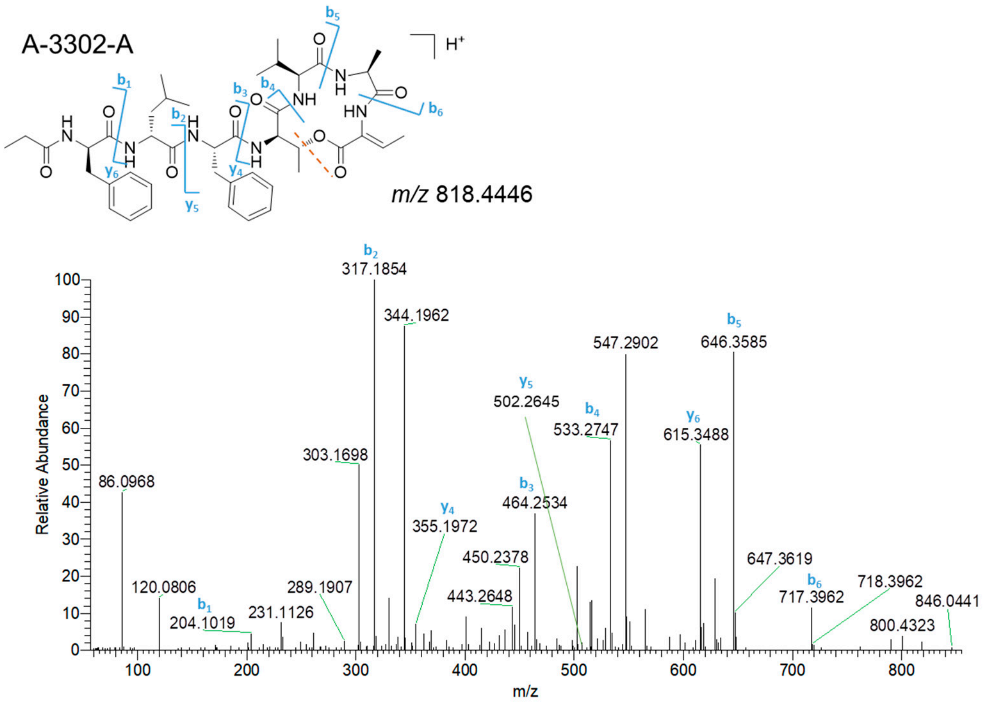

Figure S27. HRMS² spectrum of the [M+H]⁺ pseudomolecular ion of A-3302-A.

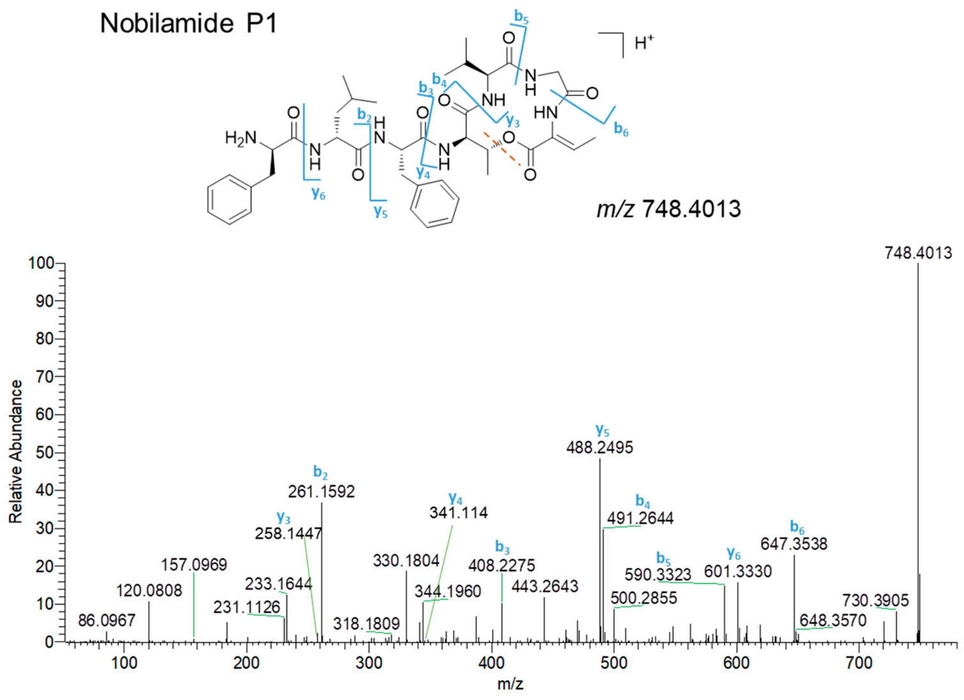

Figure S28. HRMS^2 spectrum of the $[M+H]^+$ pseudomolecular ion of Nobilamide P1.

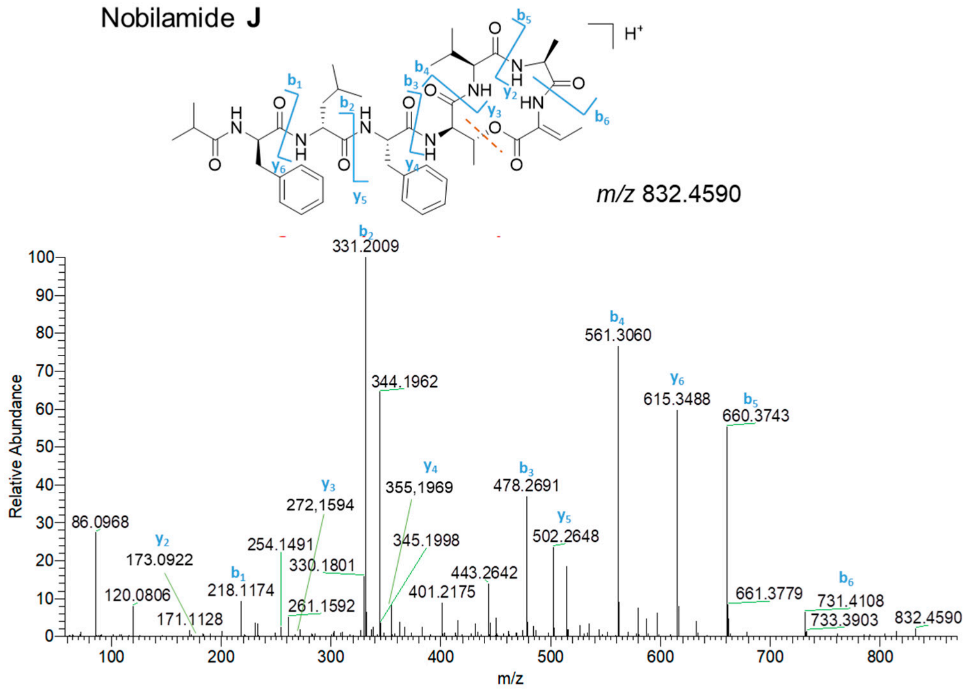

Figure S29. HRMS^2 spectrum of the $[M+H]^+$ pseudomolecular ion of Nobilamide J.

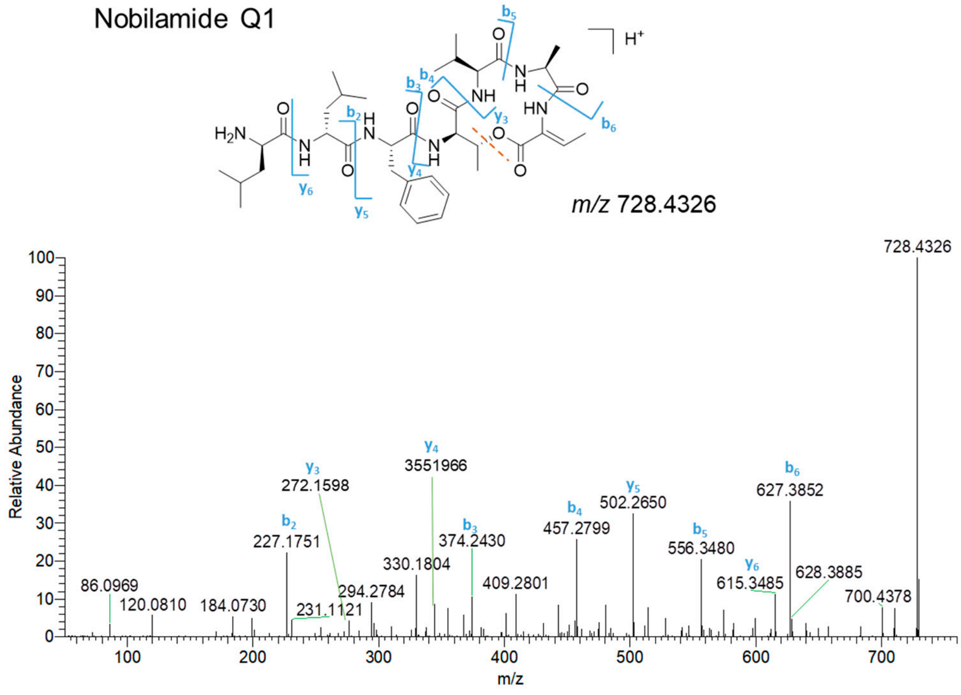

Figure S30. HRMS² spectrum of the $[M+H]^+$ pseudomolecular ion of Nobilamide Q1.

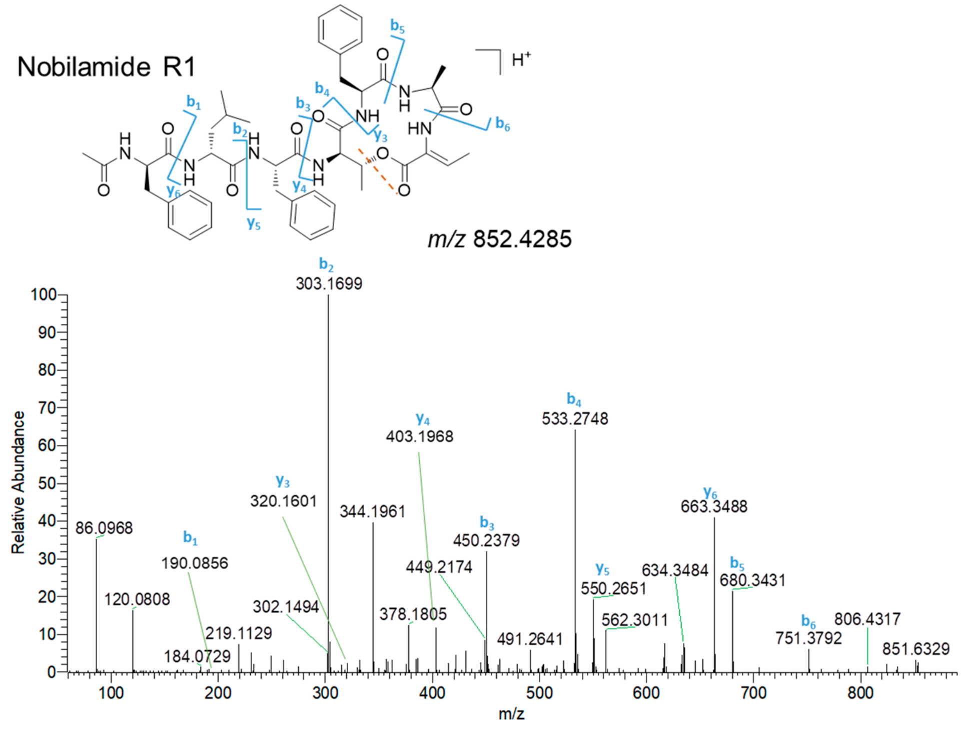

Figure S31. HRMS² spectrum of the $[M+H]^+$ pseudomolecular ion of Nobilamide R1.

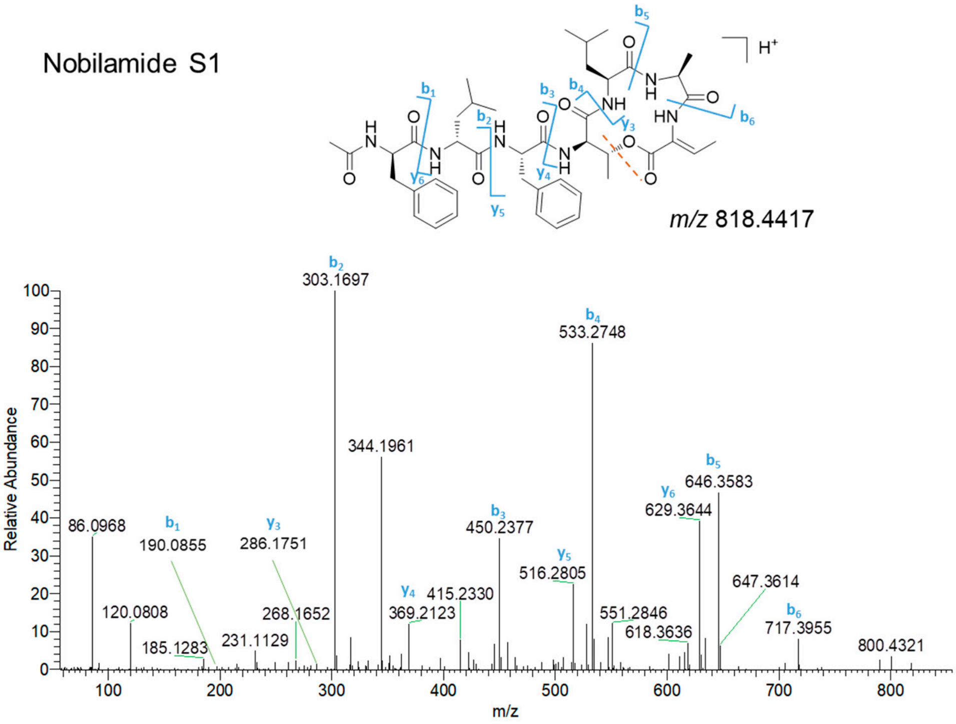

Figure S32. HRMS[2] spectrum of the [M+H][+] pseudomolecular ion of Nobilamide S1.

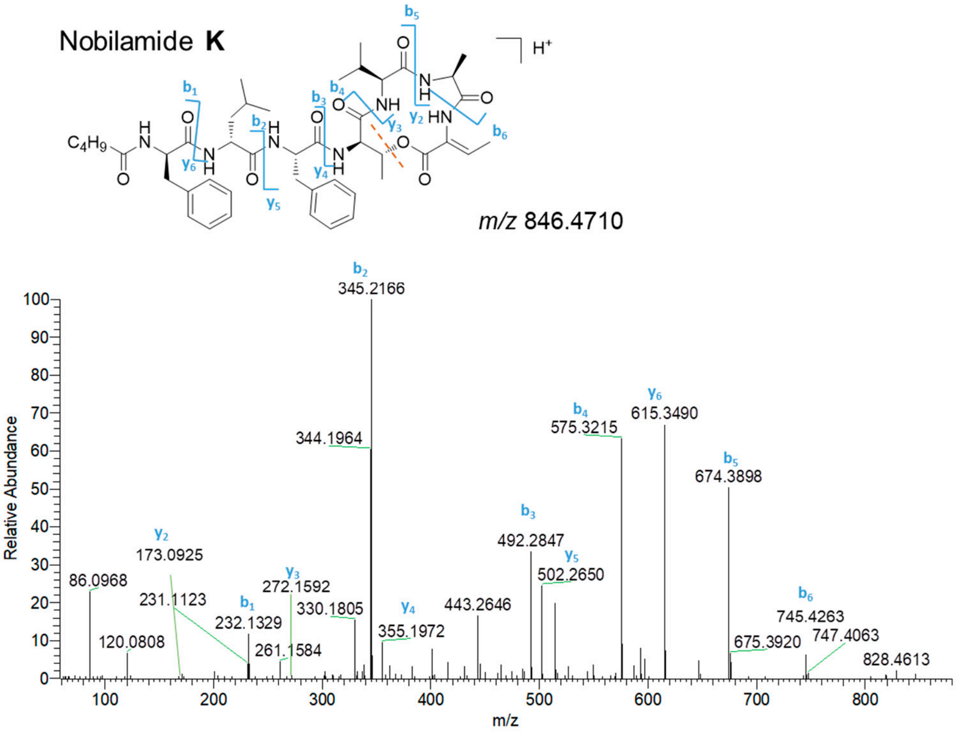

Figure S33. HRMS[2] spectrum of the [M+H][+] pseudomolecular ion of Nobilamide K.

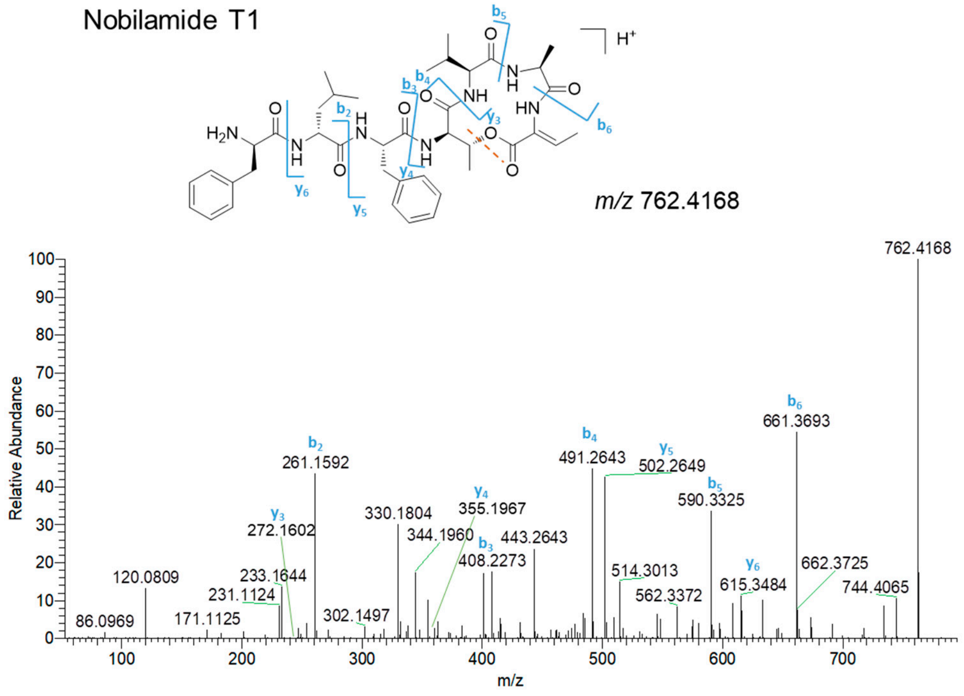

Figure S34. HRMS^2 spectrum of the $[M+H]^+$ pseudomolecular ion of Nobilamide T1.

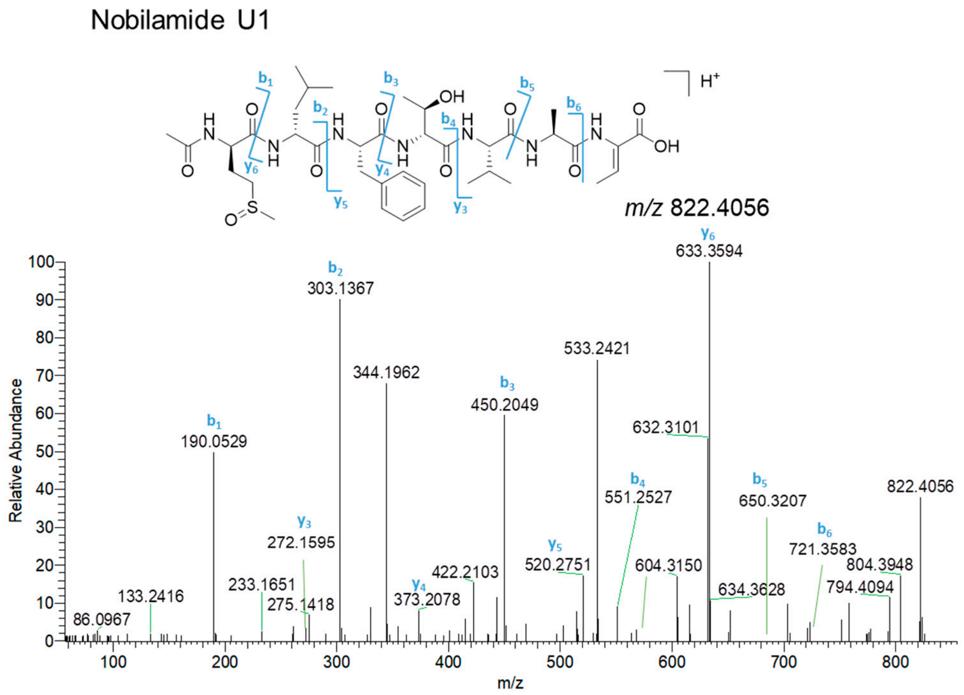

Figure S35. HRMS^2 spectrum of the $[M+H]^+$ pseudomolecular ion of Nobilamide U1.

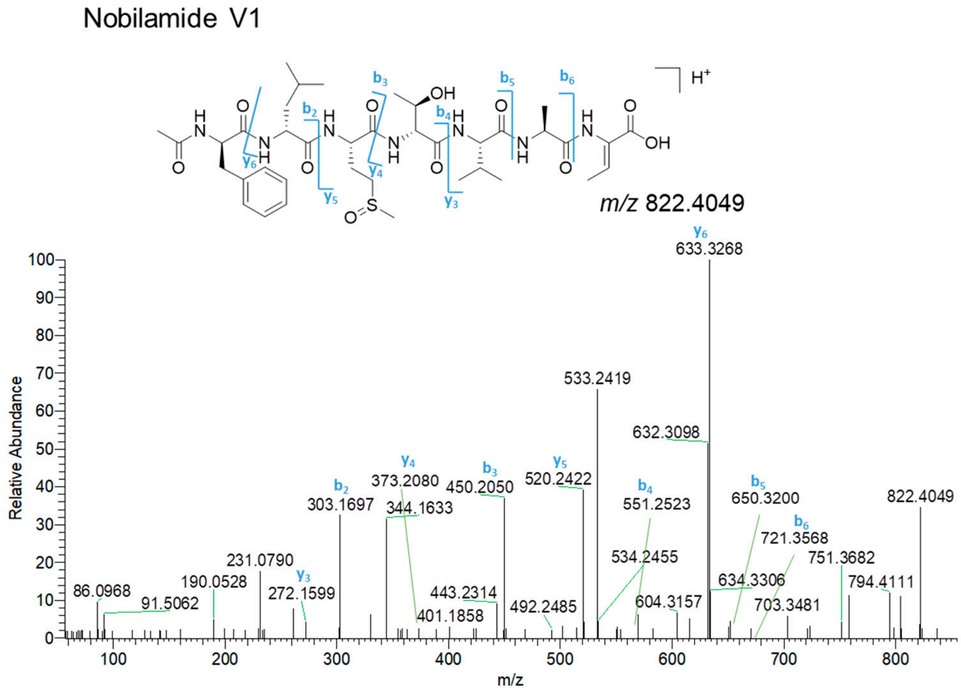

Figure S36. HRMS² spectrum of the [M+H]⁺ pseudomolecular ion of Nobilamide V1.

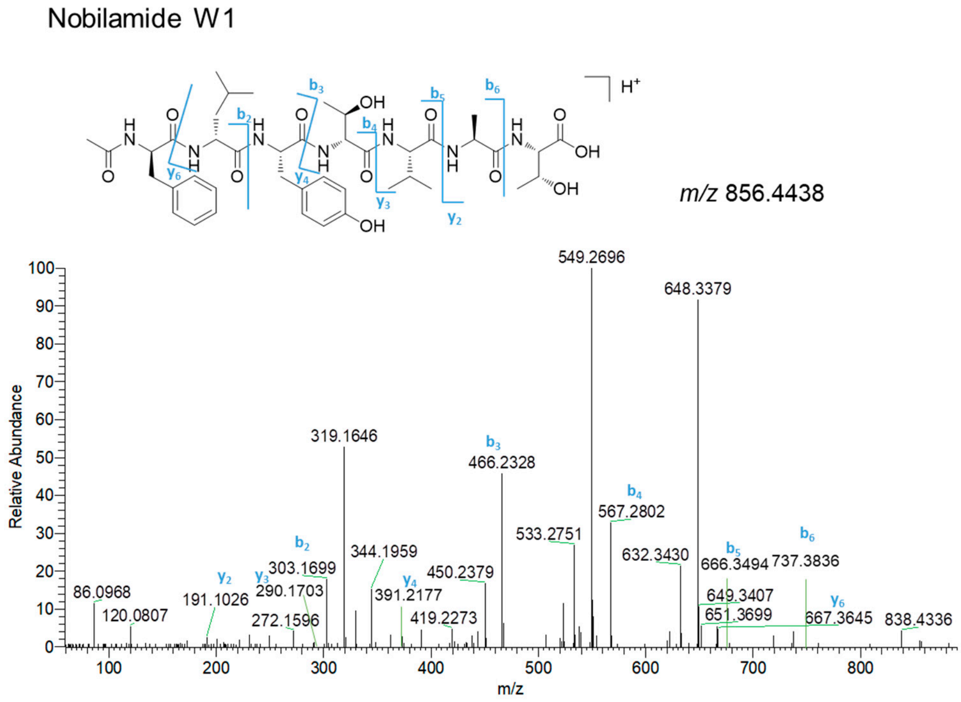

Figure S37. HRMS² spectrum of the [M+H]⁺ pseudomolecular ion of Nobilamide W1.

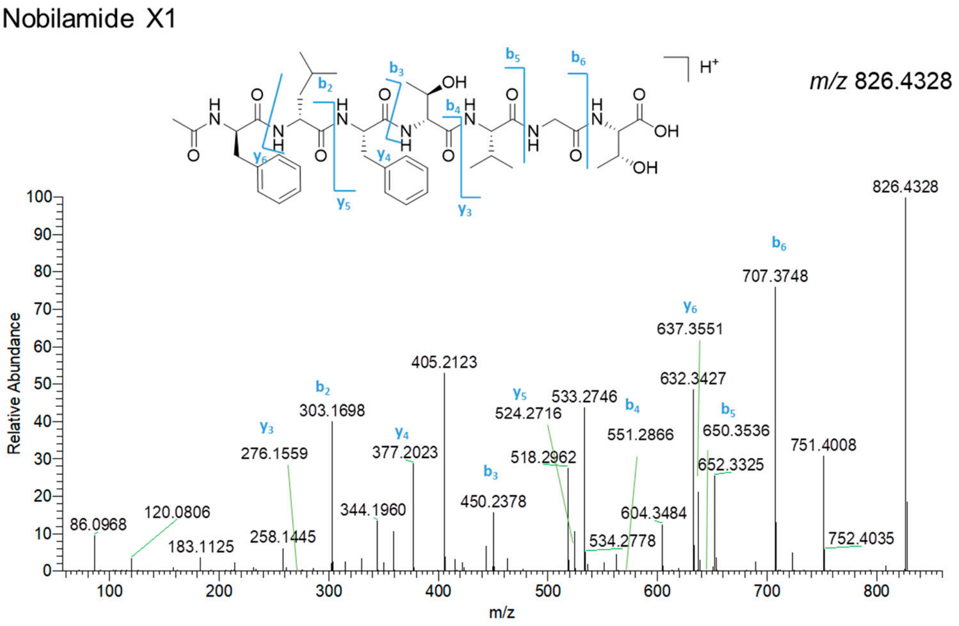

Figure S38. HRMS^2 spectrum of the $[M+H]^+$ pseudomolecular ion of Nobilamide X1.

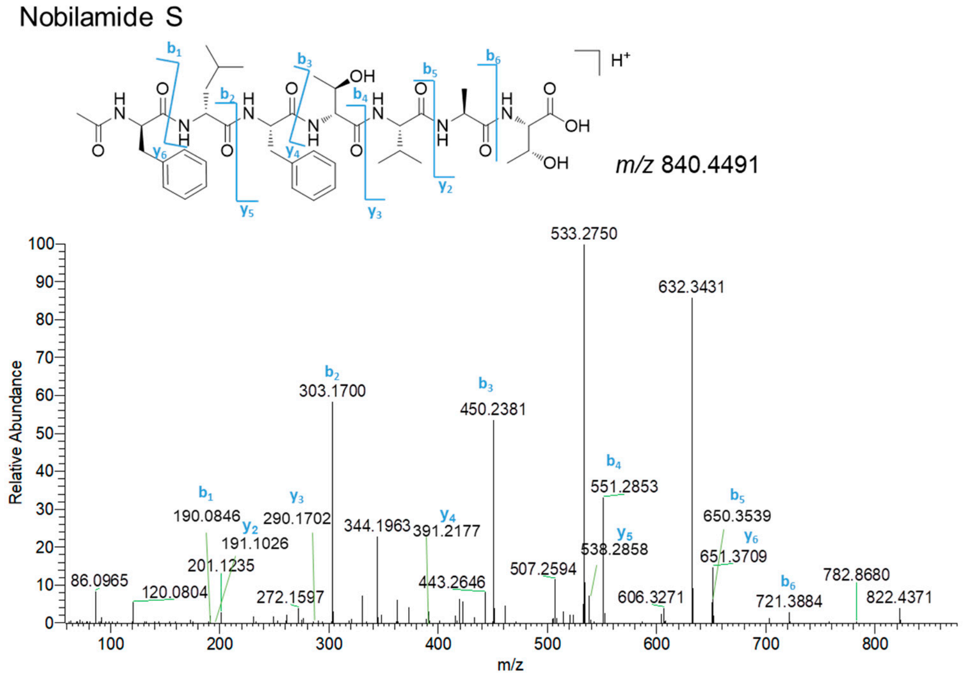

Figure S39. HRMS^2 spectrum of the $[M+H]^+$ pseudomolecular ion of Nobilamide S.

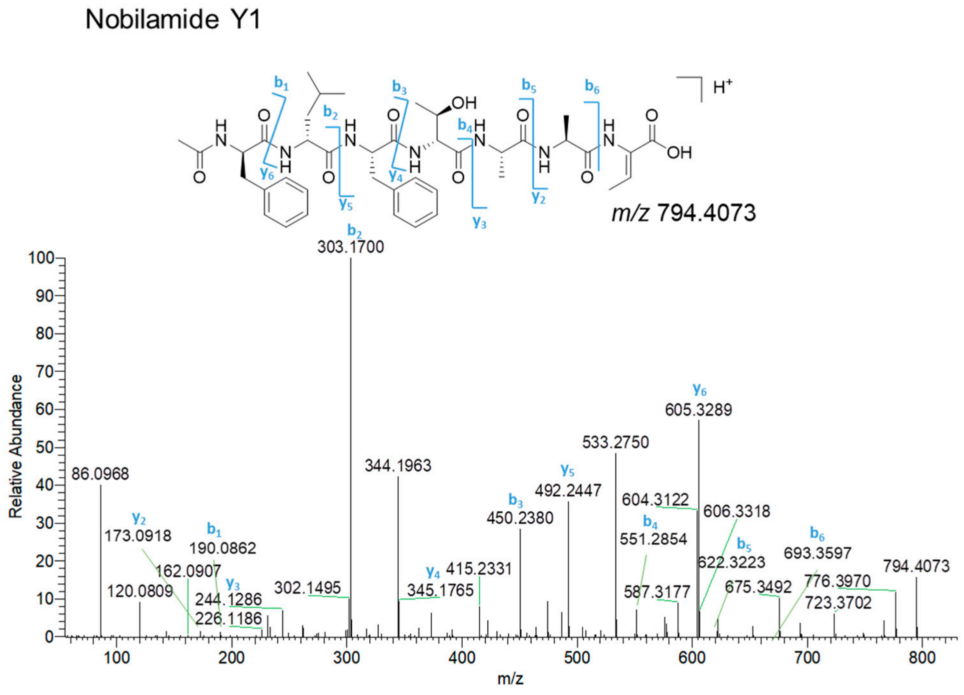

Figure S40. HRMS² spectrum of the $[M+H]^+$ pseudomolecular ion of Nobilamide Y1.

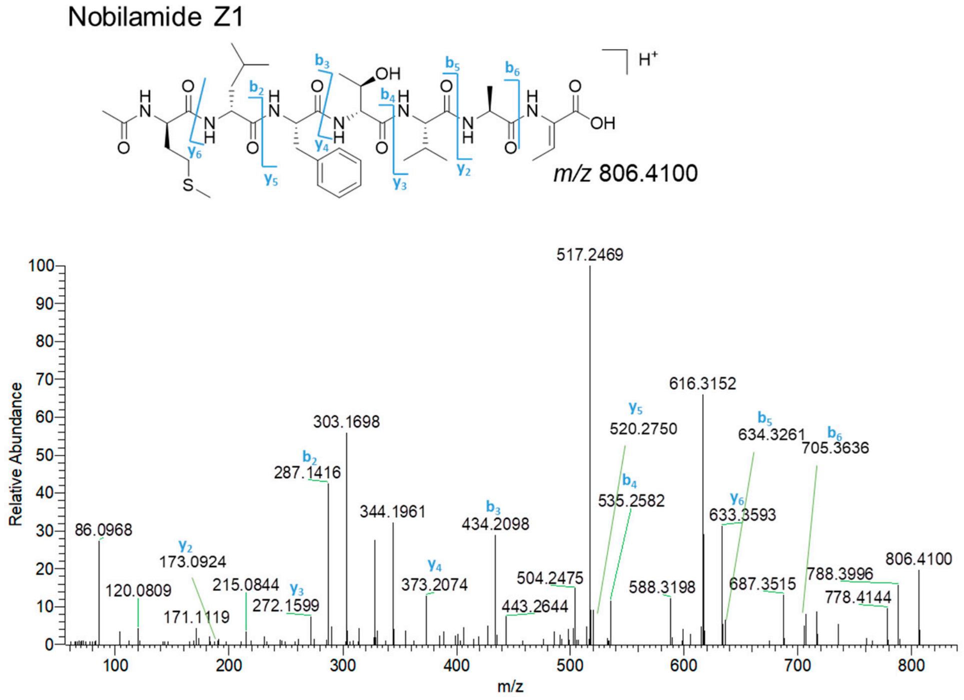

Figure S41. HRMS² spectrum of the $[M+H]^+$ pseudomolecular ion of Nobilamide Z1.

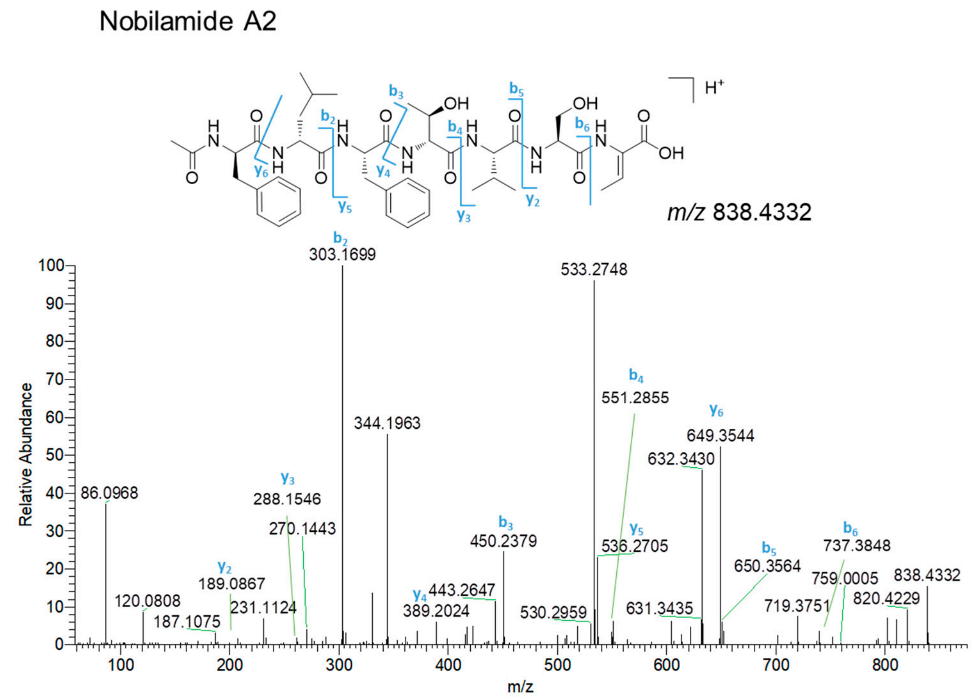

Figure S42. HRMS² spectrum of the [M+H]⁺ pseudomolecular ion of Nobilamide A2.

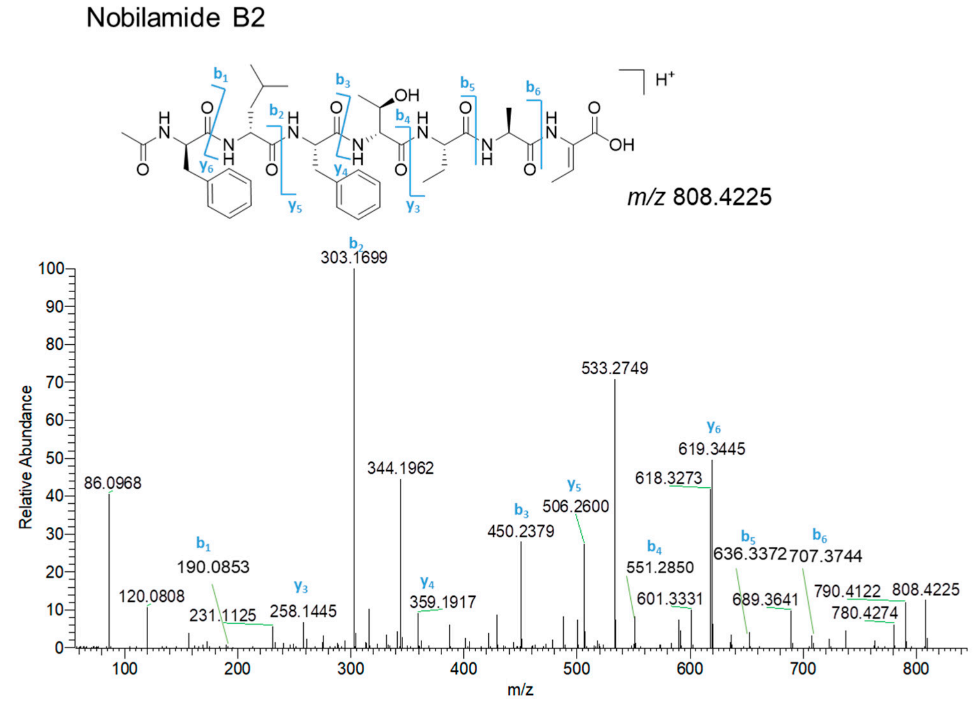

Figure S43. HRMS² spectrum of the [M+H]⁺ pseudomolecular ion of Nobilamide B2.

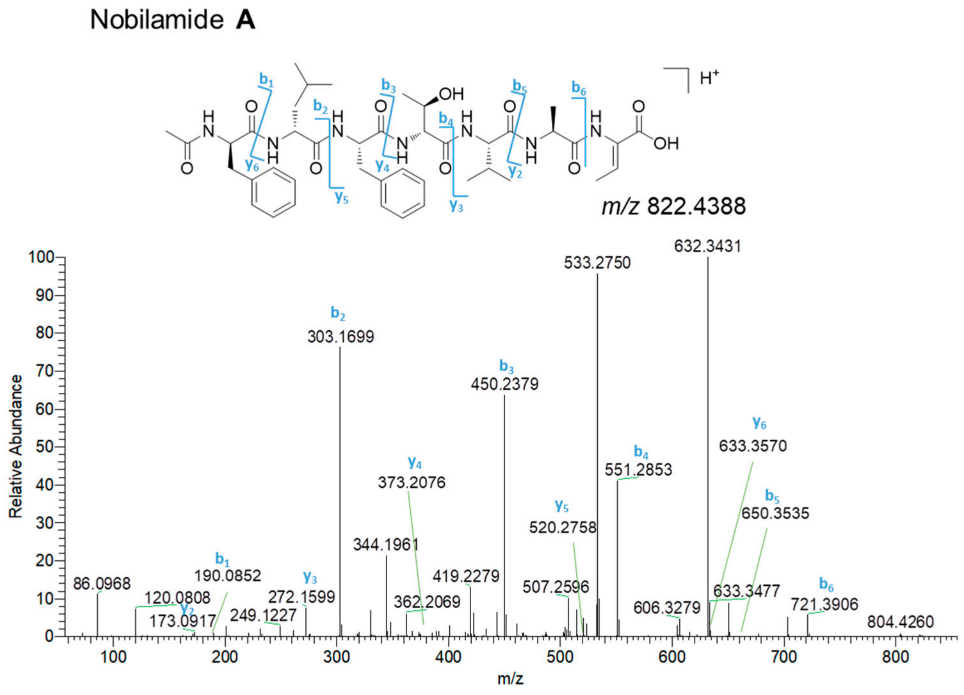

Figure S44. HRMS^2 spectrum of the $[M+H]^+$ pseudomolecular ion of Nobilamide A.

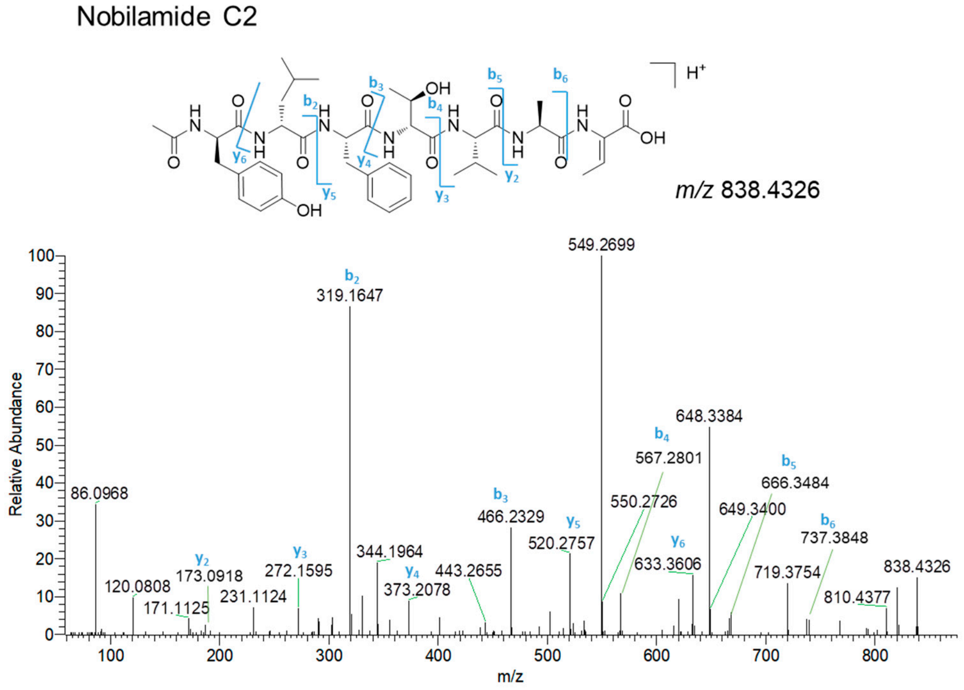

Figure S45. HRMS^2 spectrum of the $[M+H]^+$ pseudomolecular ion of Nobilamide C2.

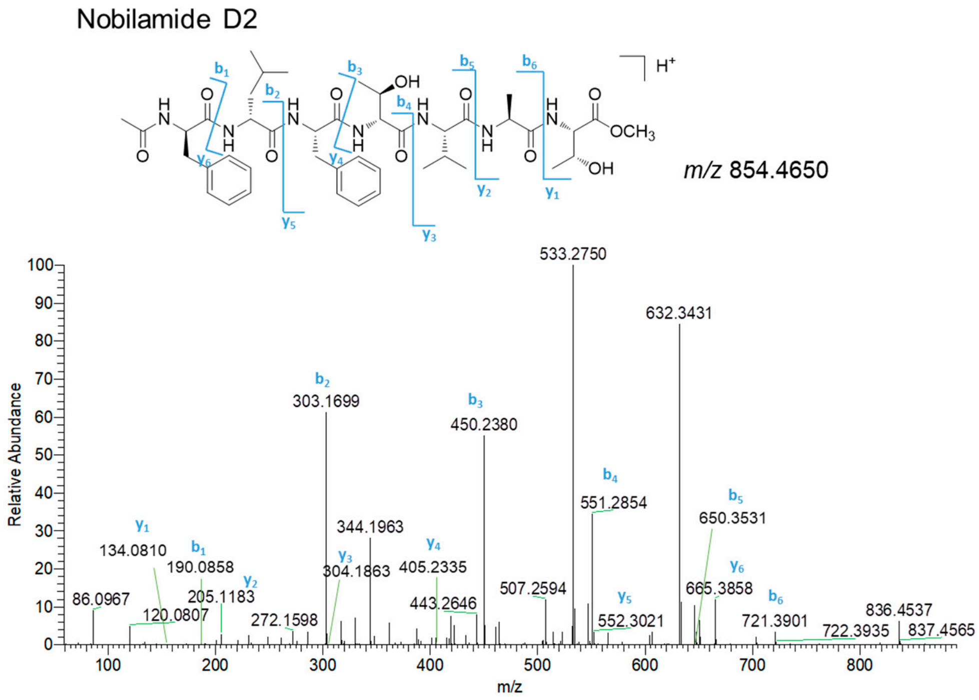

Figure S46. HRMS[2] spectrum of the [M+H][+] pseudomolecular ion of Nobilamide D2.

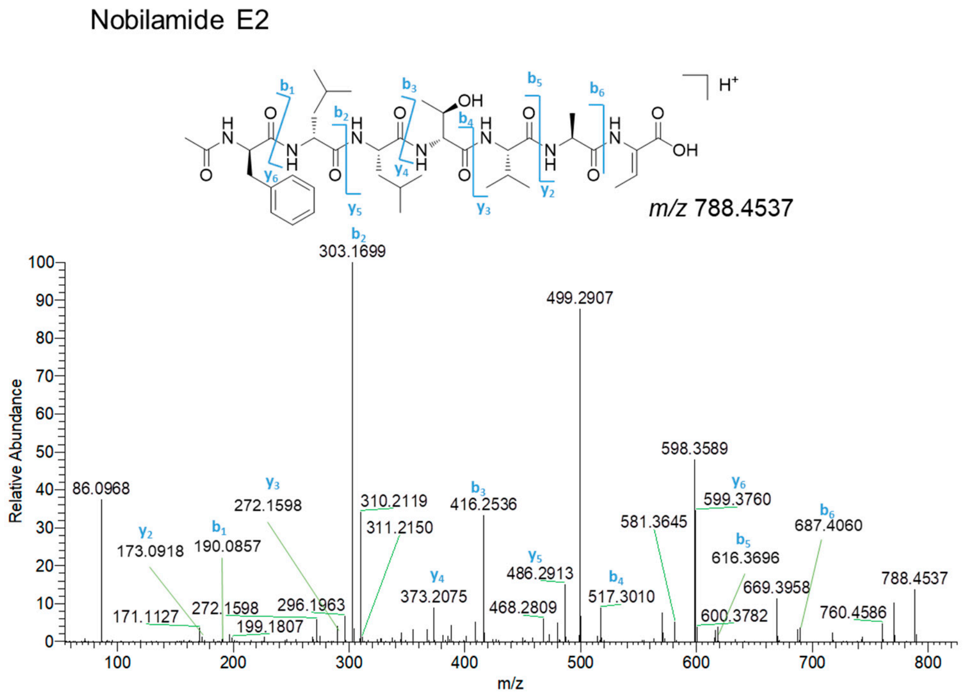

Figure S47. HRMS[2] spectrum of the [M+H][+] pseudomolecular ion of Nobilamide E2.

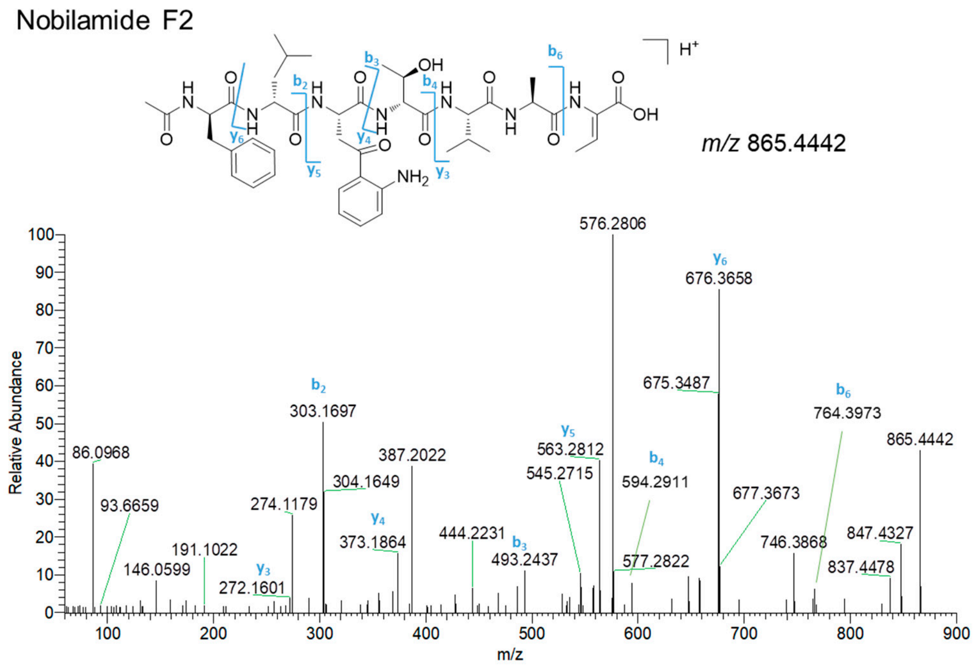

Figure S48. HRMS² spectrum of the [M+H]⁺ pseudomolecular ion of Nobilamide F2.

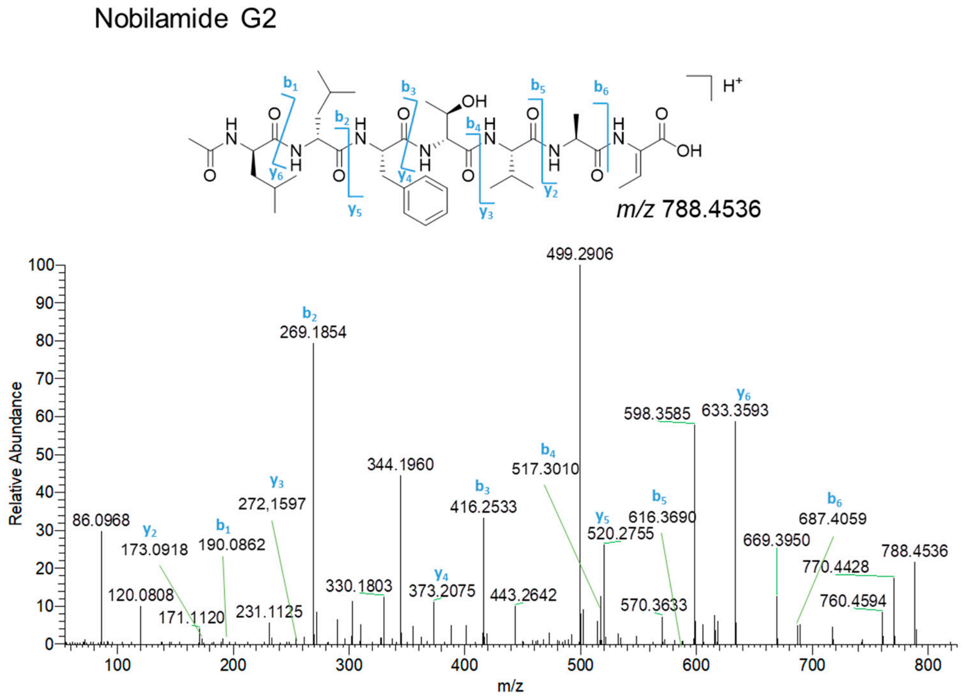

Figure S49. HRMS² spectrum of the [M+H]⁺ pseudomolecular ion of Nobilamide G2.

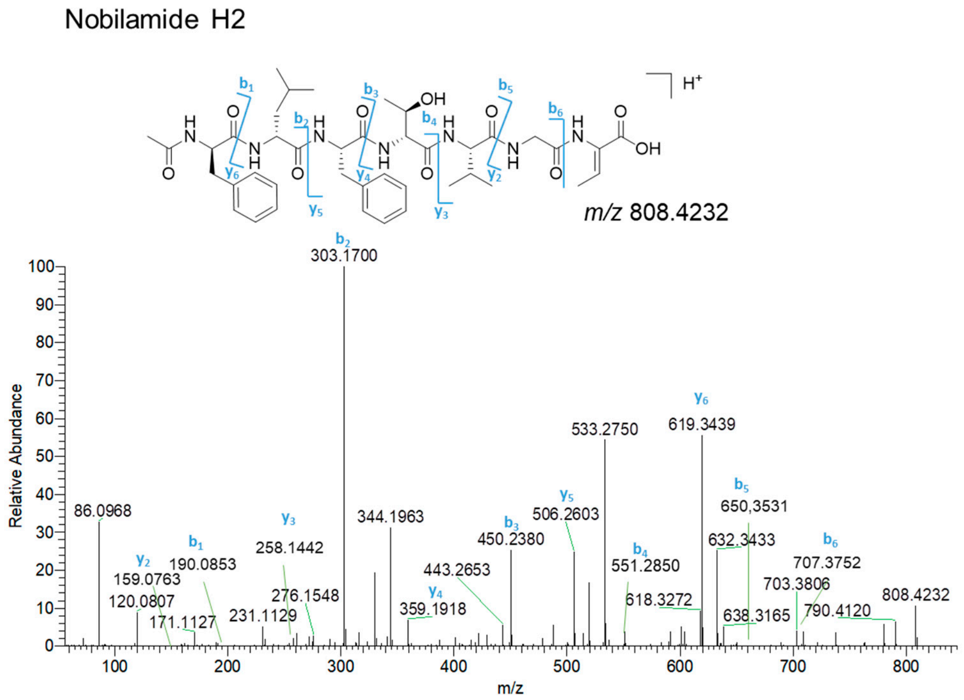

Figure S50. HRMS² spectrum of the [M+H]⁺ pseudomolecular ion of Nobilamide H2.

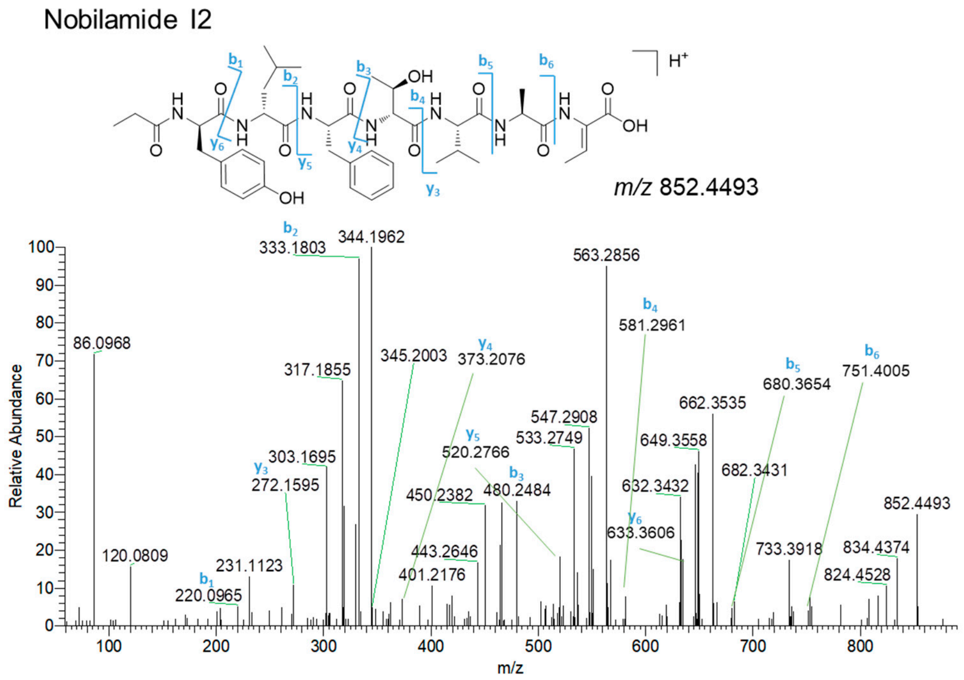

Figure S51. HRMS² spectrum of the [M+H]⁺ pseudomolecular ion of Nobilamide I2.

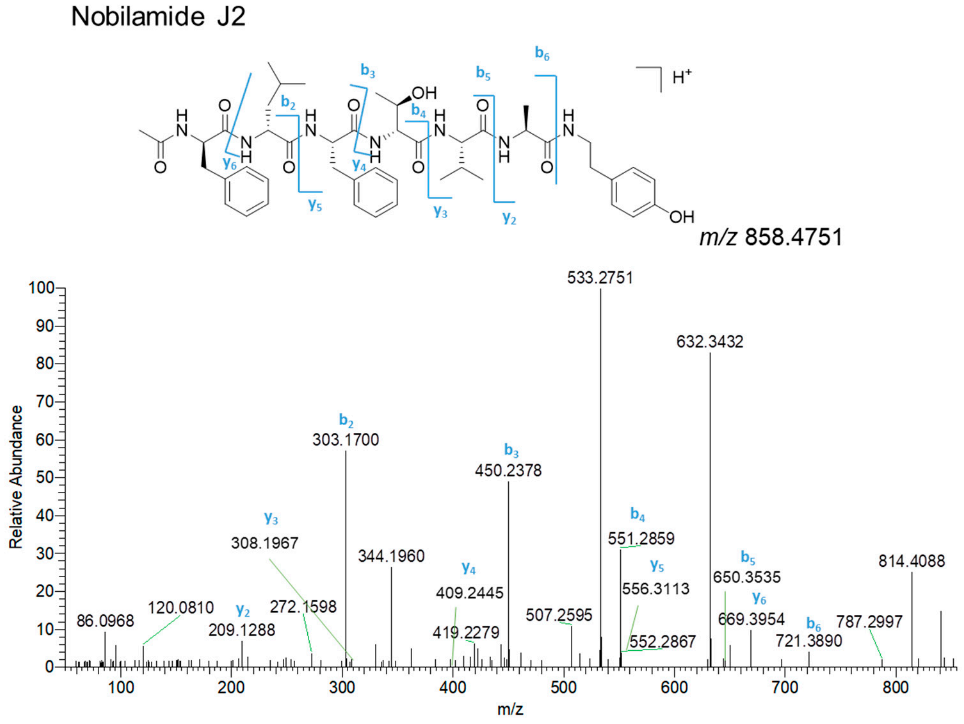

Figure S52. HRMS² spectrum of the [M+H]⁺ pseudomolecular ion of Nobilamide J2.

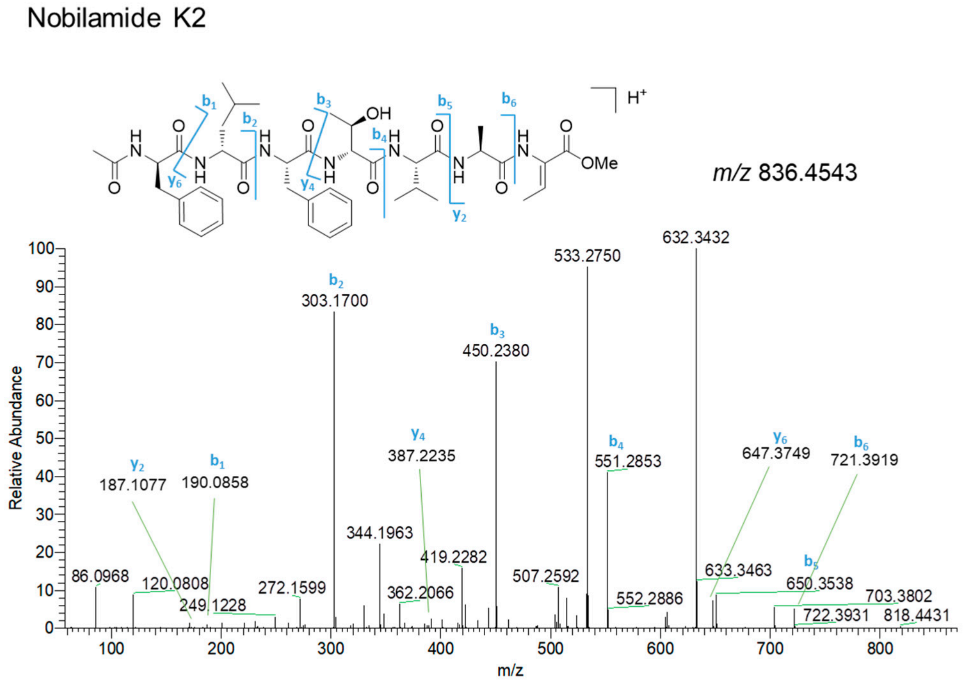

Figure S53. HRMS² spectrum of the [M+H]⁺ pseudomolecular ion of Nobilamide K2.

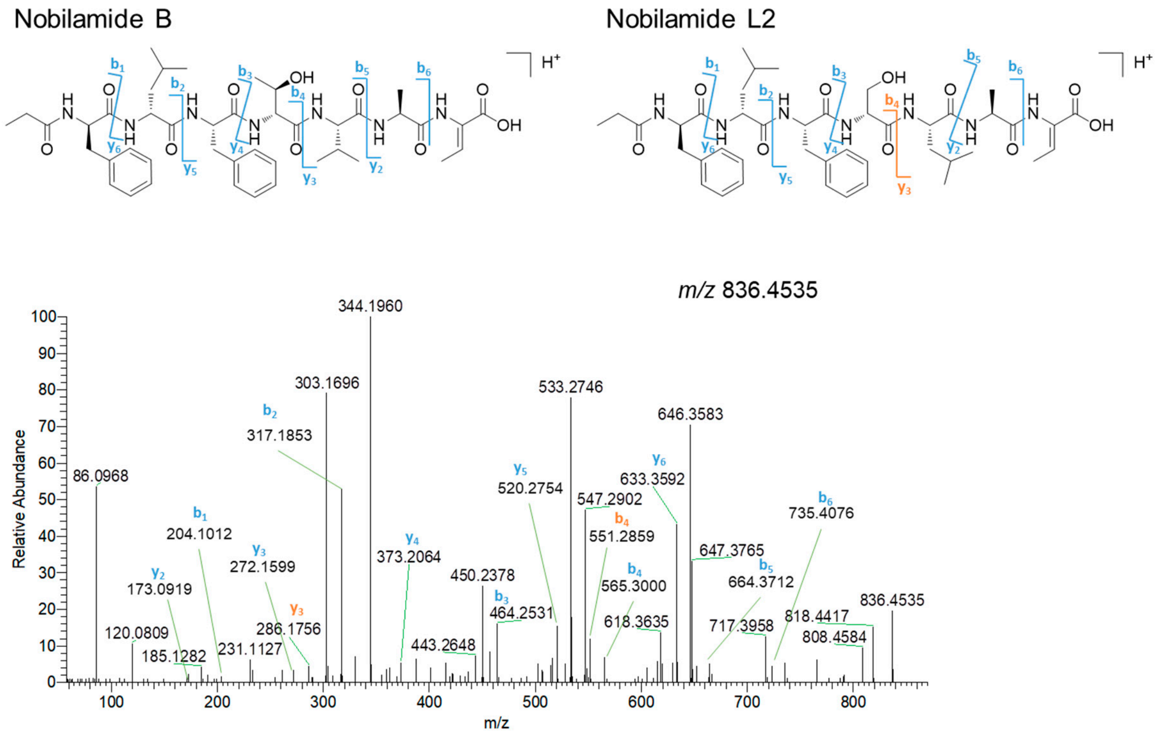

Figure S54. HRMS² spectra of the [M+H]⁺ pseudomolecular ions of Nobilamide B and Nobilamide L2.

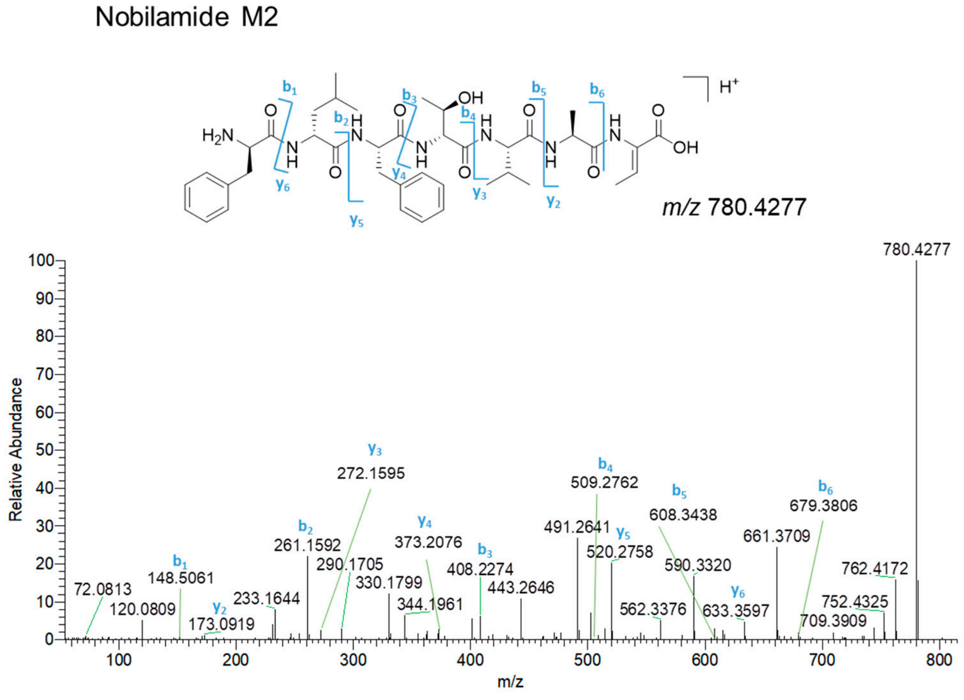

Figure S55. HRMS² spectrum of the [M+H]⁺ pseudomolecular ion of Nobilamide M2.

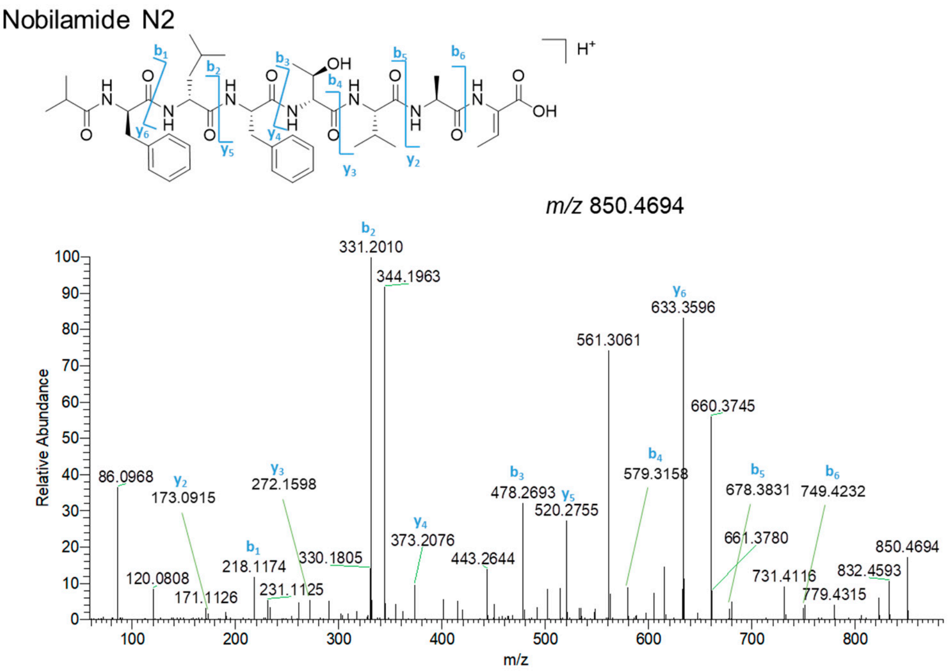

Figure S56. HRMS[2] spectrum of the [M+H]+ pseudomolecular ion of Nobilamide N2.

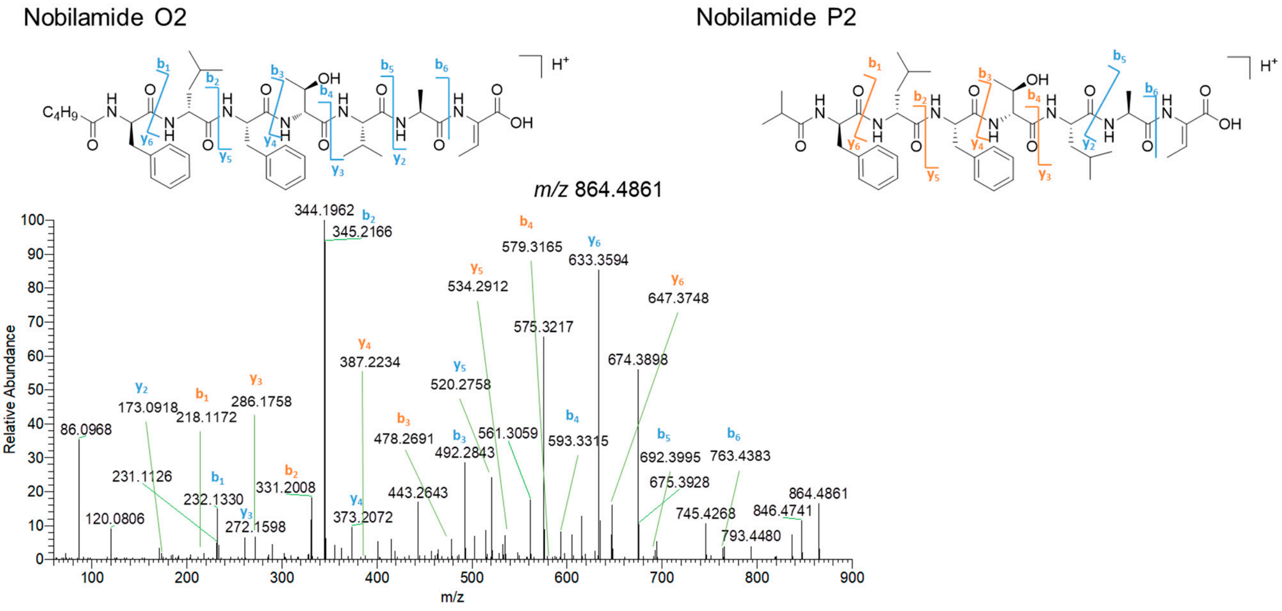

Figure S57. HRMS[2] spectra of the [M+H]+ pseudomolecular ions of Nobilamide O2 and Nobilamide P2.

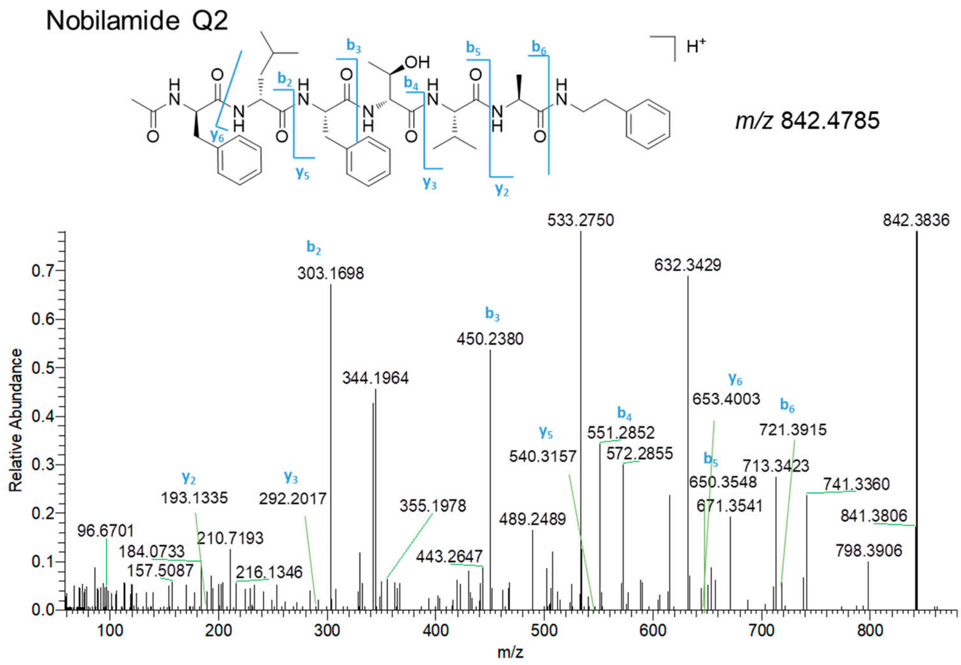

Figure S58. HRMS² spectrum of the [M+H]⁺ pseudomolecular ion of Nobilamide Q2.

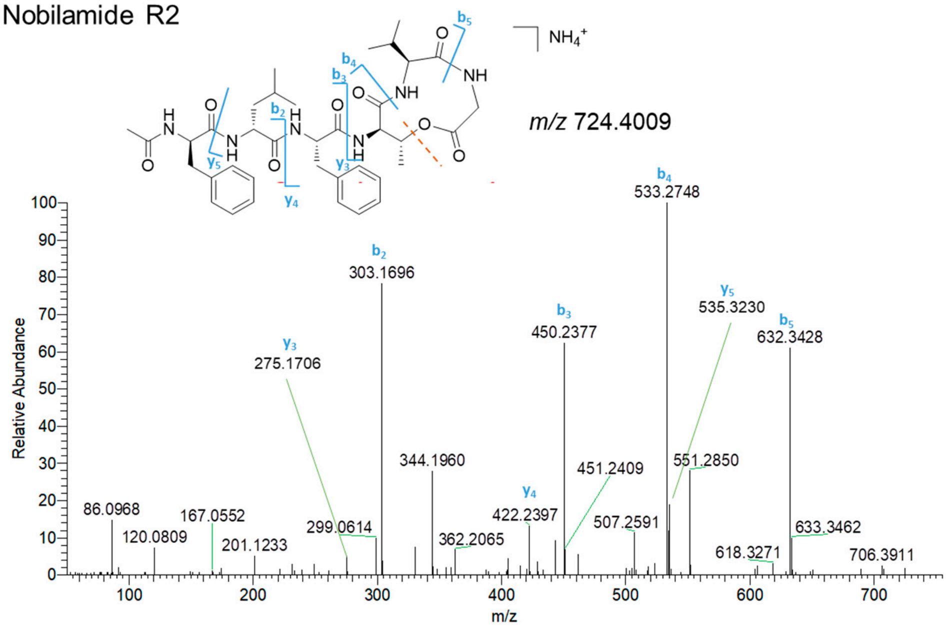

Figure S59. HRMS² spectrum of the [M+NH₄]⁺ pseudomolecular ion of Nobilamide R2.

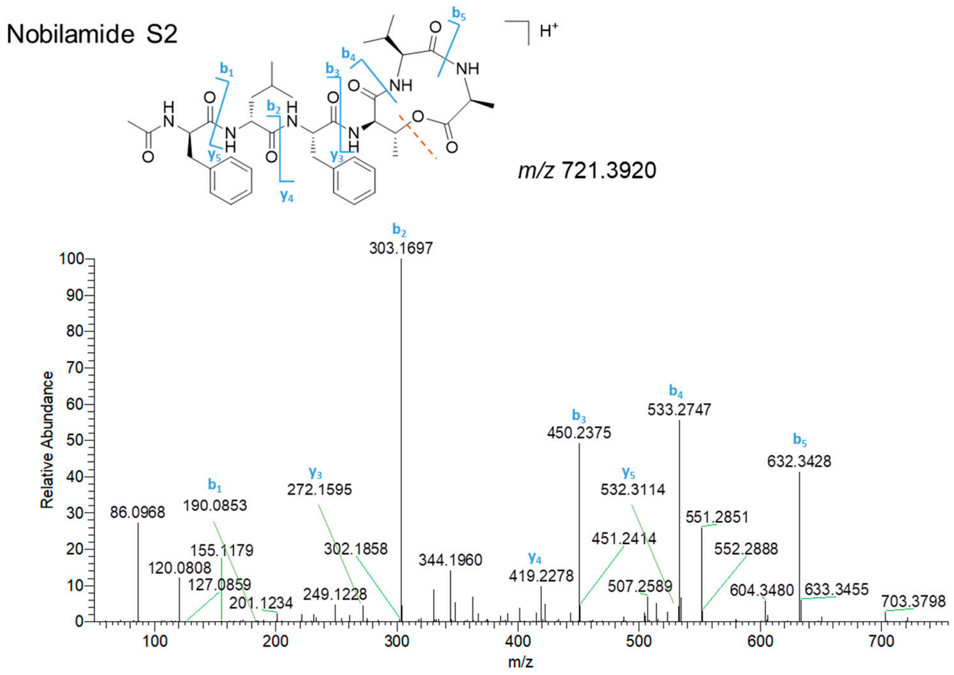

Figure S60. HRMS² spectrum of the [M+H]⁺ pseudomolecular ion of Nobilamide S2.

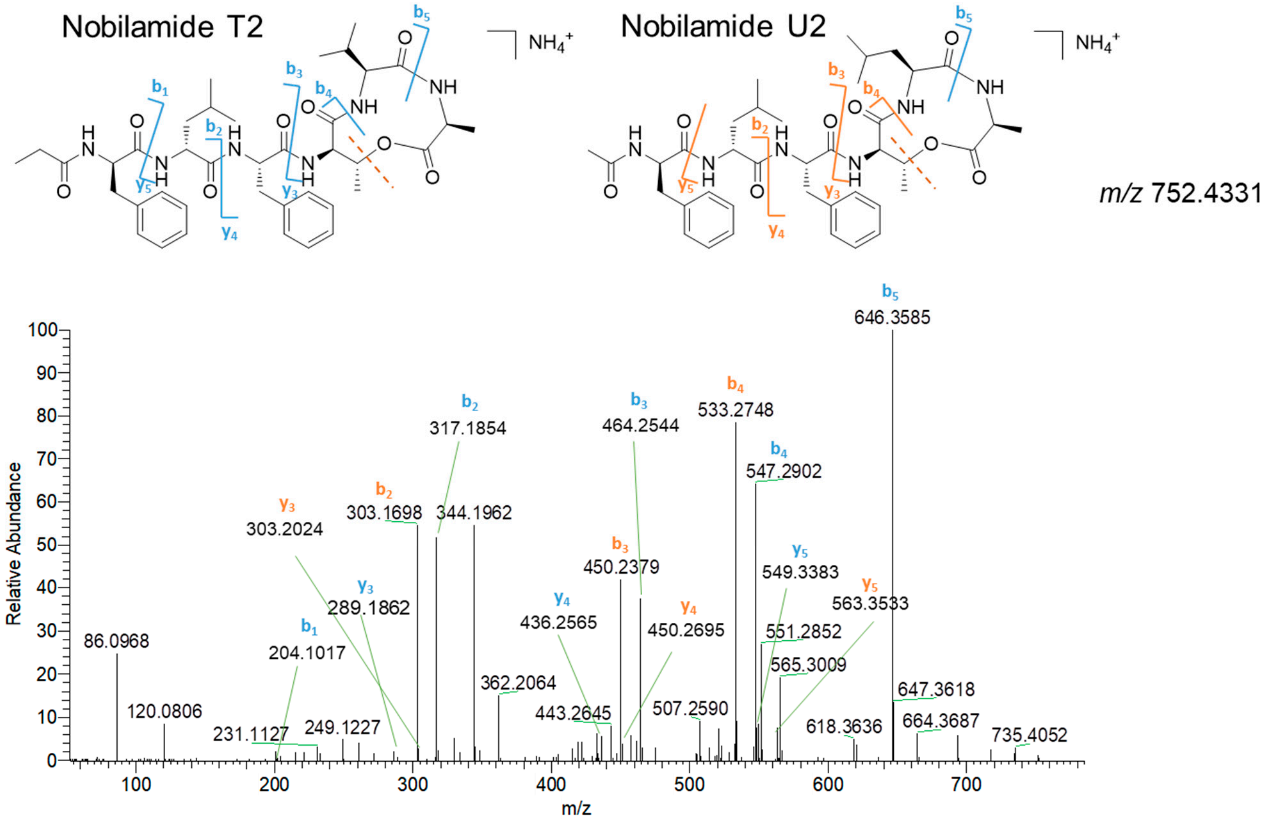

Figure S61. HRMS² spectra of the [M+NH₄]⁺ pseudomolecular ions of Nobilamide T2 and Nobilamide U2.

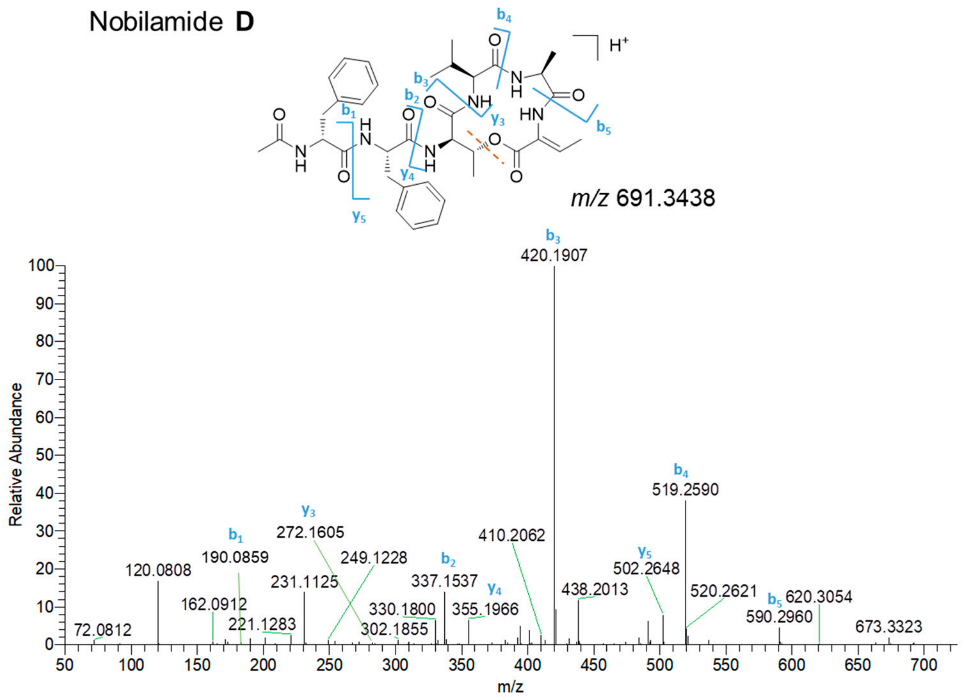

Figure S62. HRMS² spectrum of the $[M+H]^+$ pseudomolecular ion of Nobilamide D.

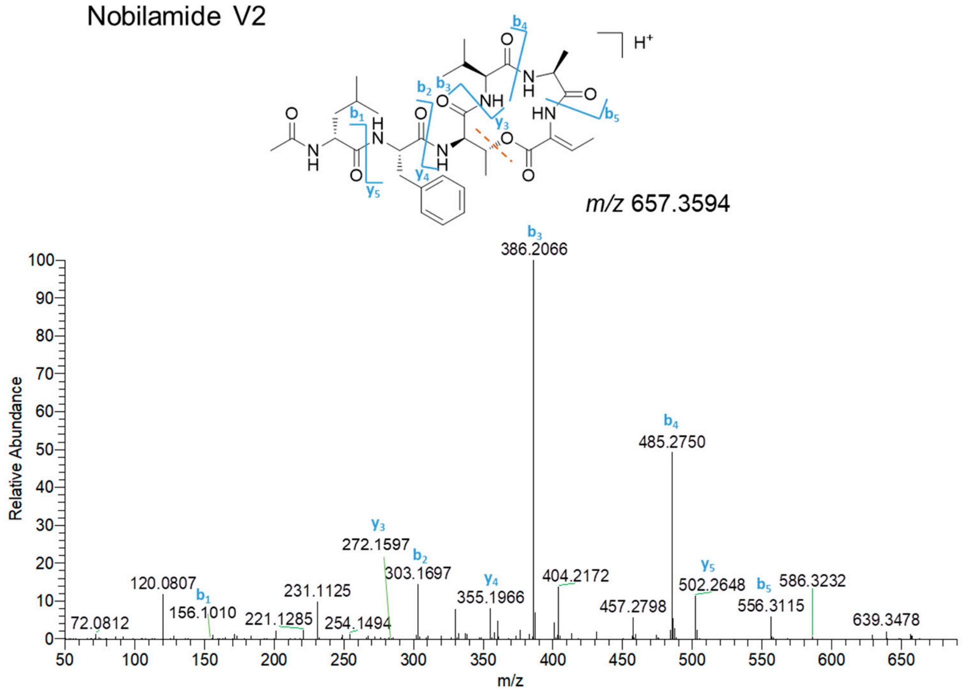

Figure S63. HRMS² spectrum of the $[M+H]^+$ pseudomolecular ion of Nobilamide V2.

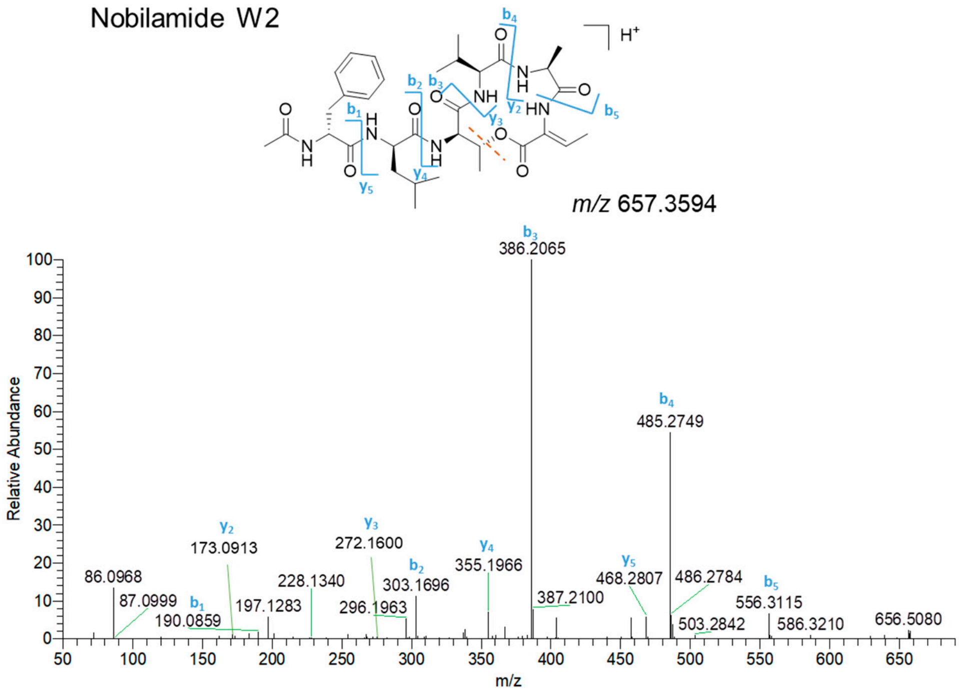

Figure S64. HRMS[2] spectrum of the [M+H]+ pseudomolecular ion of Nobilamide W2.

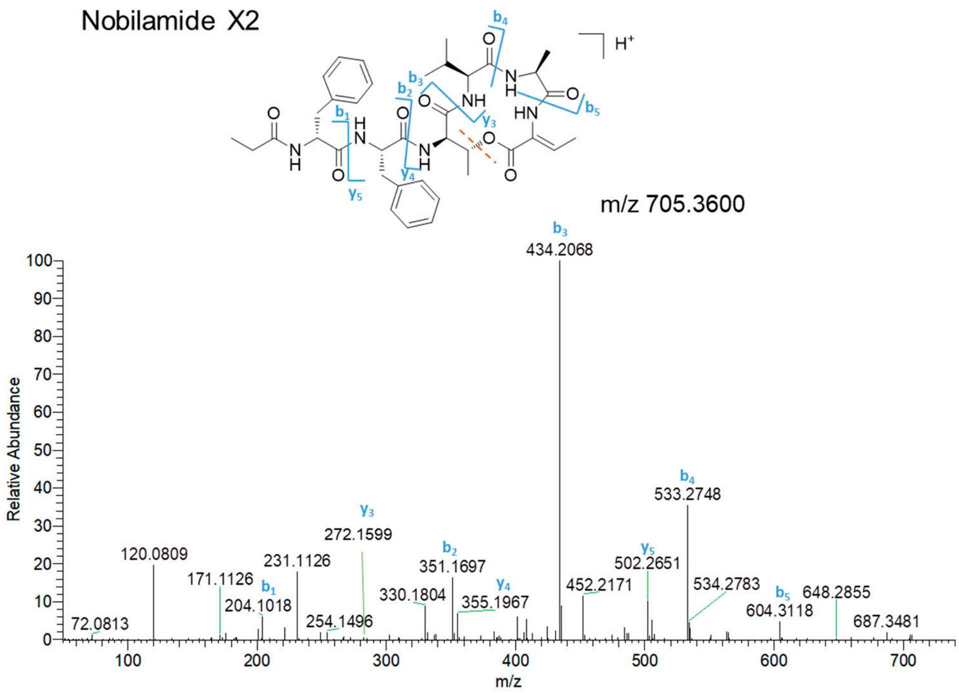

Figure S65. HRMS[2] spectrum of the [M+H]+ pseudomolecular ion of Nobilamide X2.

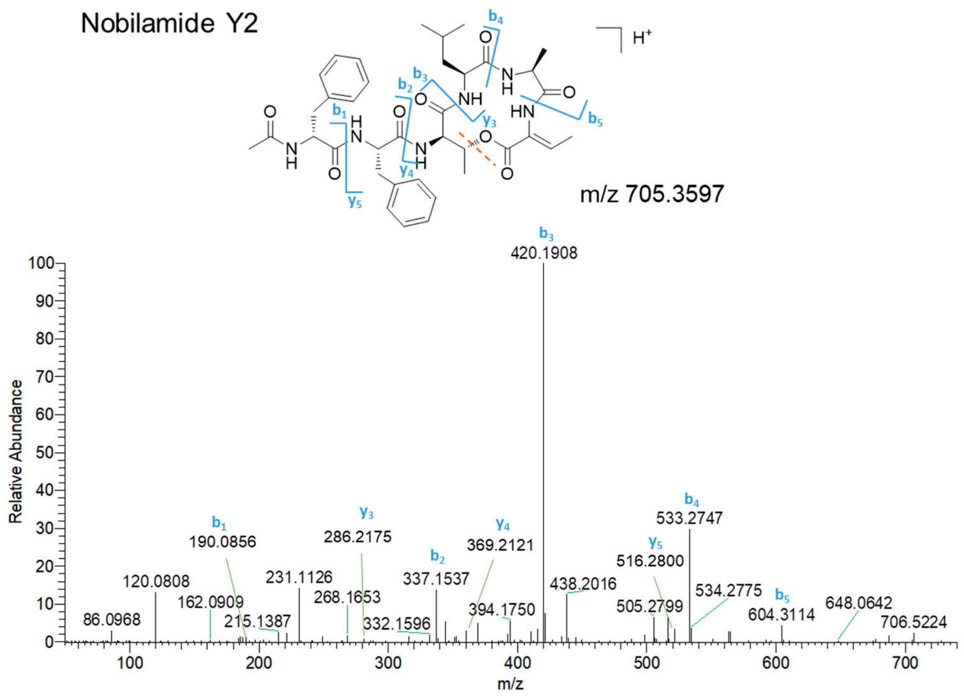

Figure S66. HRMS² spectrum of the [M+H]⁺ pseudomolecular ion of Nobilamide Y2.

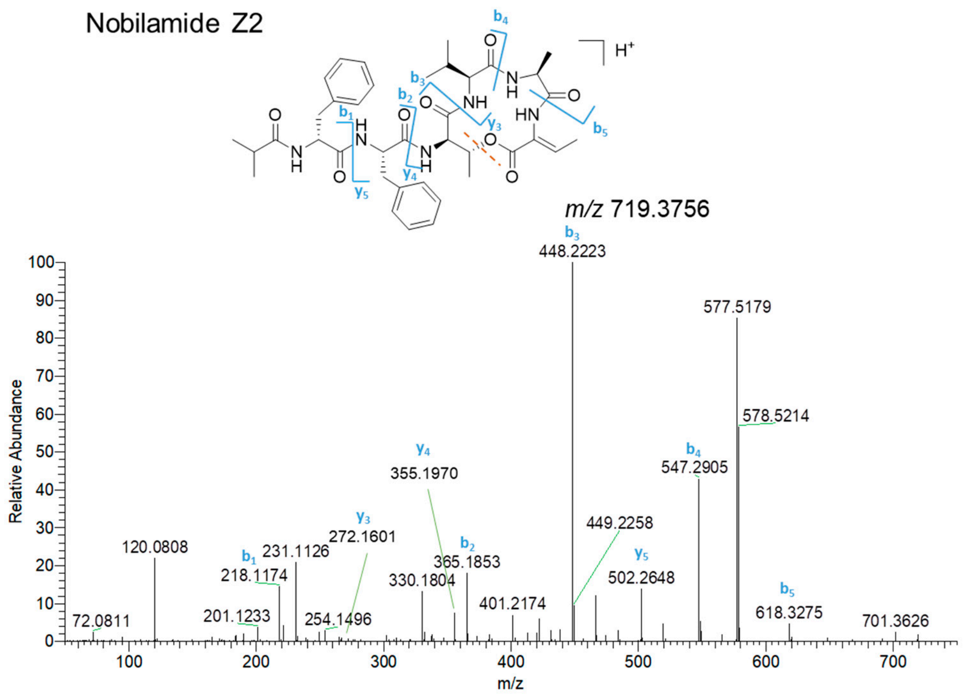

Figure S67. HRMS² spectrum of the [M+H]⁺ pseudomolecular ion of Nobilamide Z2.

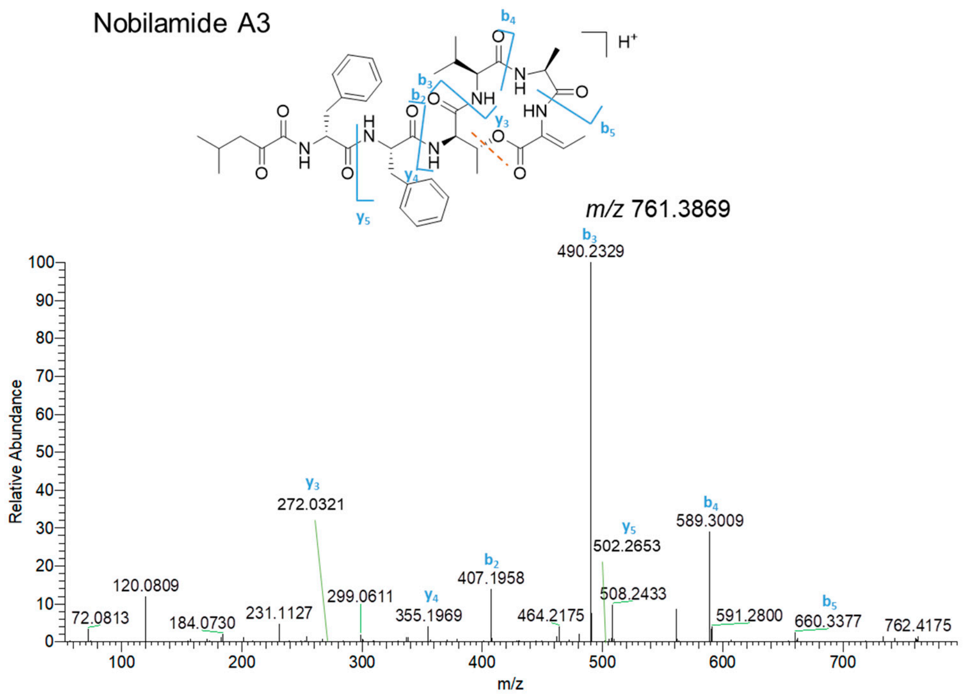

Figure S68. HRMS² spectrum of the [M+H]⁺ pseudomolecular ion of Nobilamide A3.

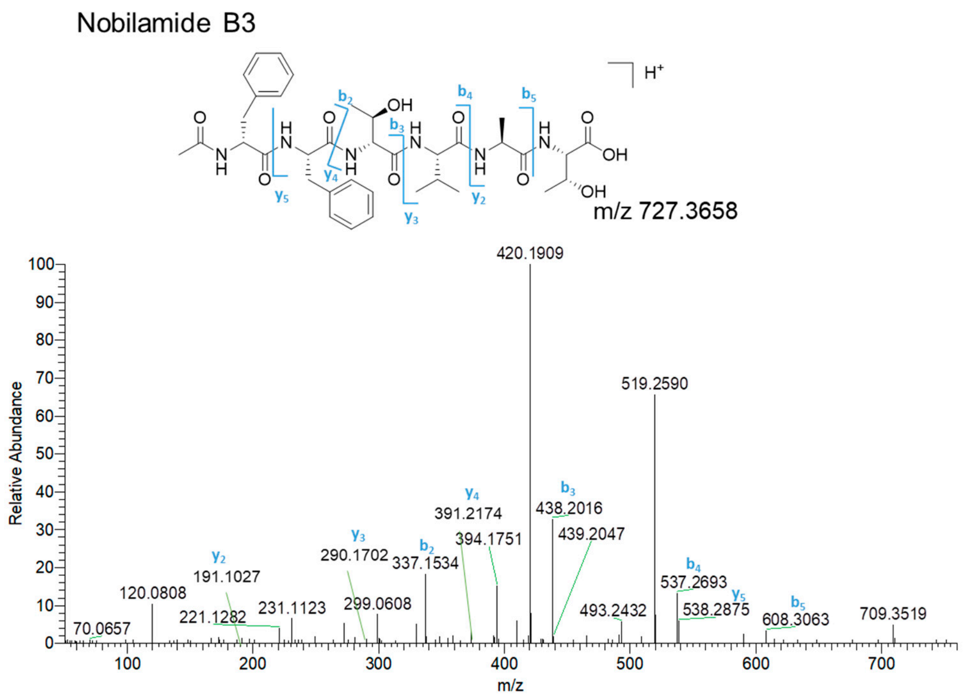

Figure S69. HRMS² spectrum of the [M+H]⁺ pseudomolecular ion of Nobilamide B3.

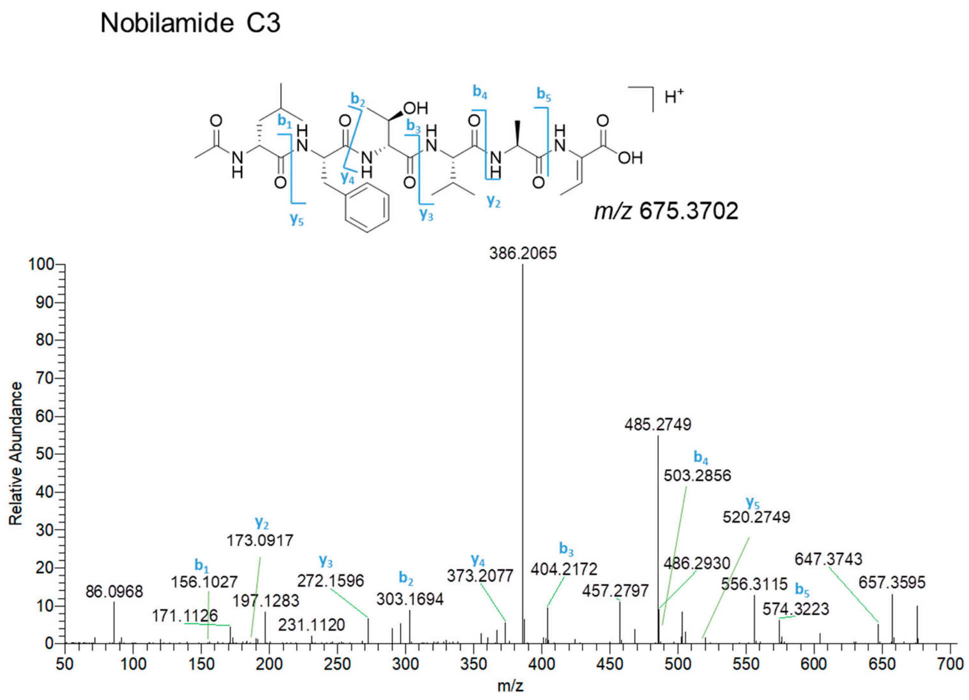

Figure S70. HRMS² spectrum of the [M+H]⁺ pseudomolecular ion of Nobilamide C3.

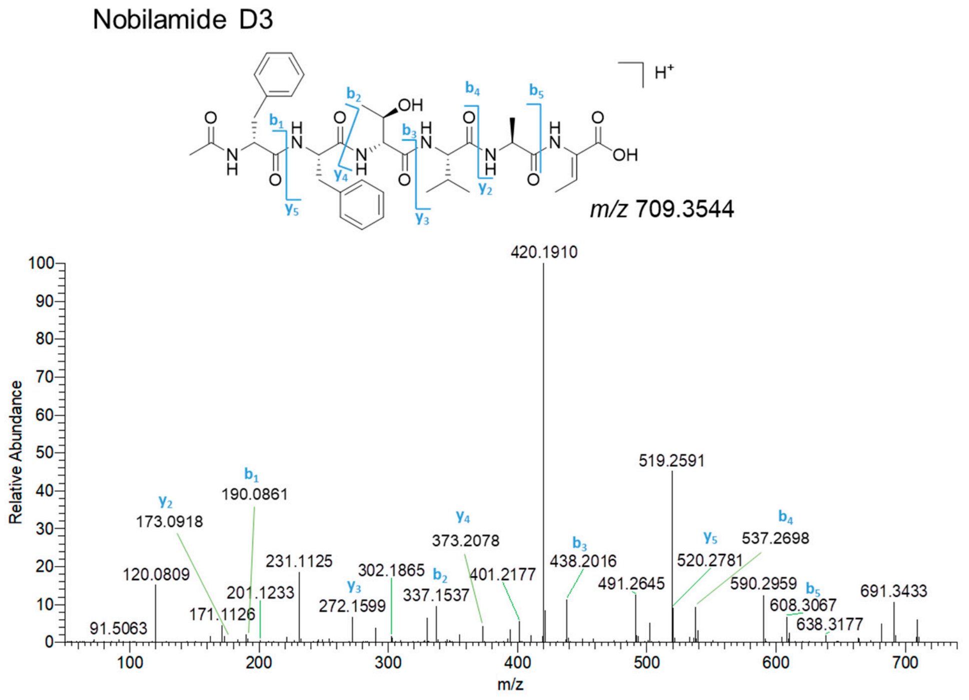

Figure S71. HRMS² spectrum of the [M+H]⁺ pseudomolecular ion of Nobilamide D3.

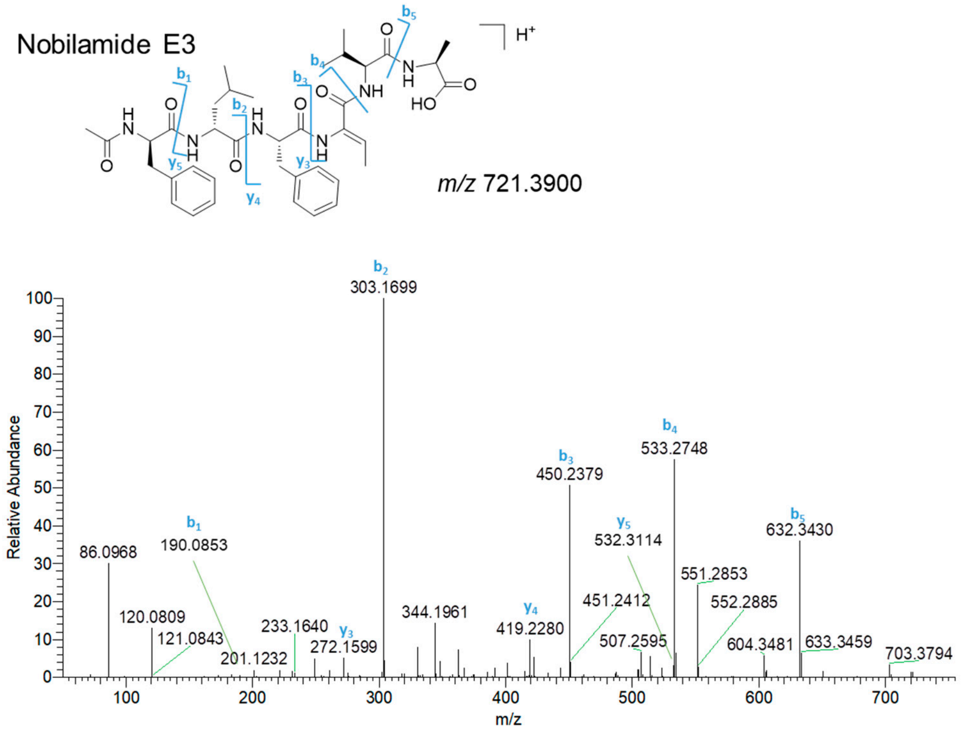

Figure S72. HRMS² spectrum of the [M+H]⁺ pseudomolecular ion of Nobilamide E3.

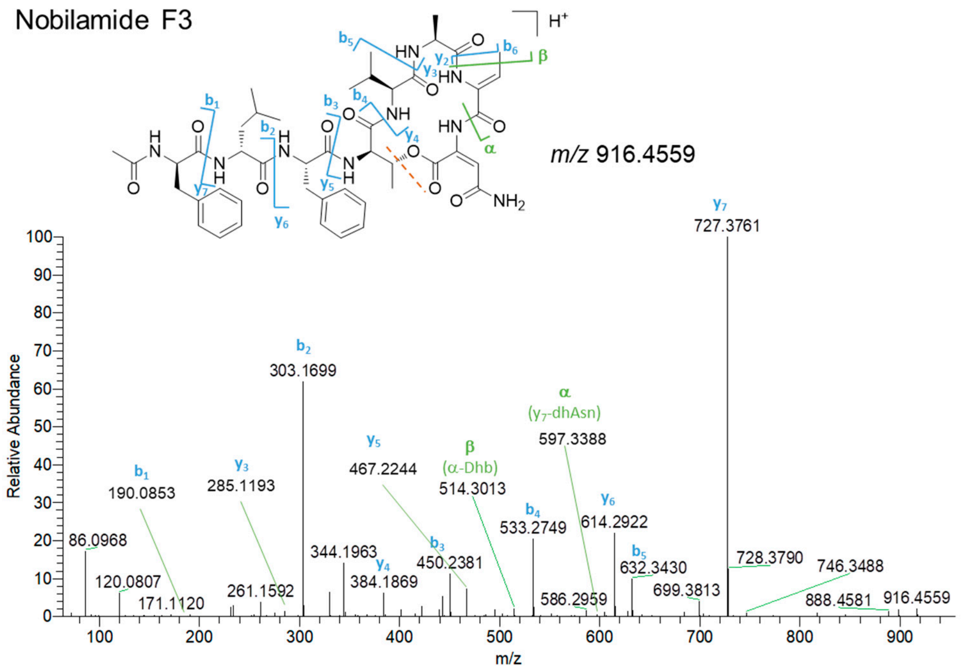

Figure S73. HRMS² spectrum of the [M+H]⁺ pseudomolecular ion of Nobilamide F3. Abbreviation: Dhb, 2,3-dehydrobutyrine; dhAsn, dehydroasparagine.

.

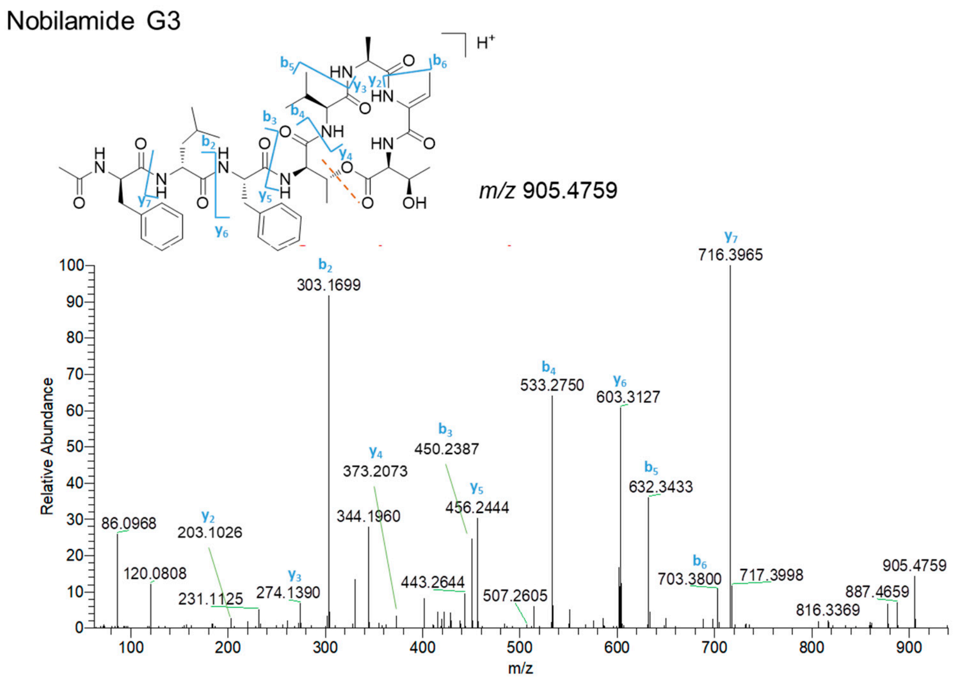

Figure S74. HRMS^2 spectrum of the $[M+H]^+$ pseudomolecular ion of Nobilamide G3.

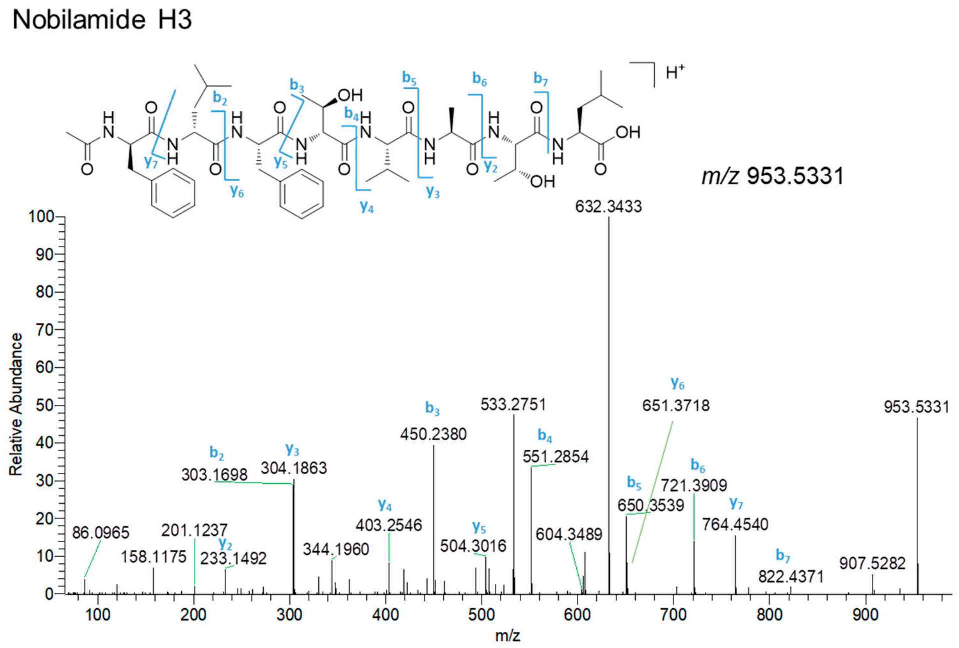

Figure S75. HRMS^2 spectrum of the $[M+H]^+$ pseudomolecular ion of Nobilamide H3.

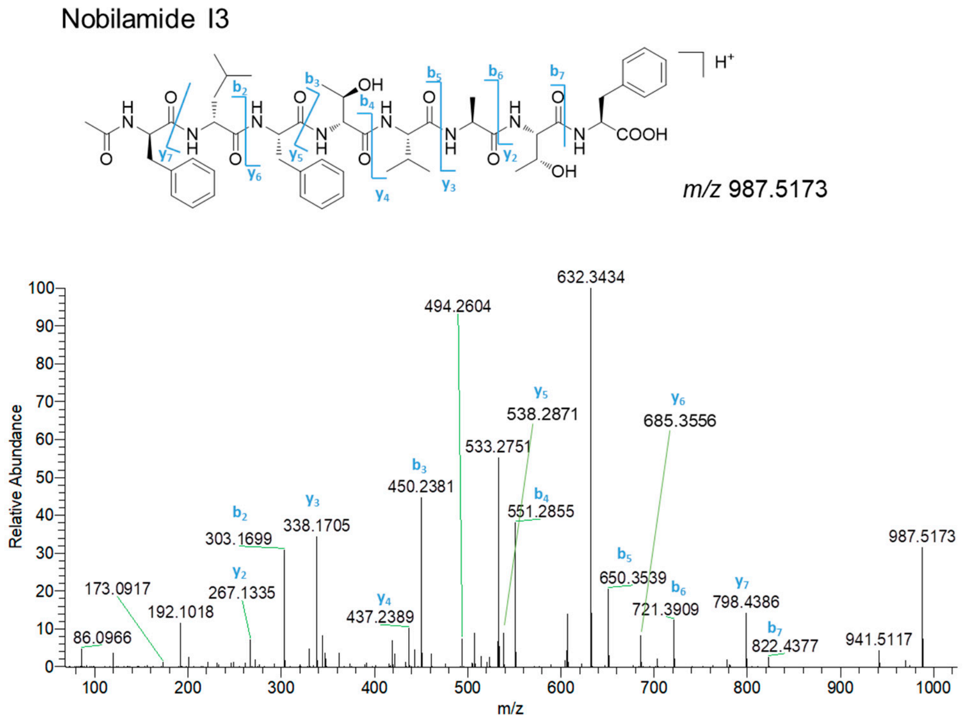

Figure S76. HRMS² spectrum of the [M+H]⁺ pseudomolecular ion of Nobilamide I3.

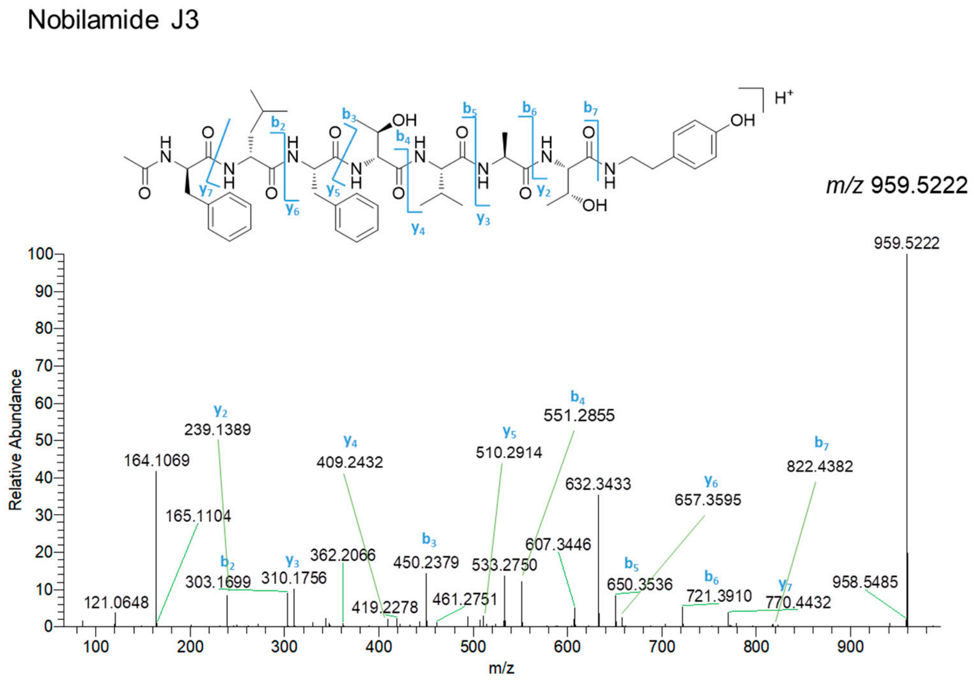

Figure S77. HRMS² spectrum of the [M+H]⁺ pseudomolecular ion of Nobilamide J3.

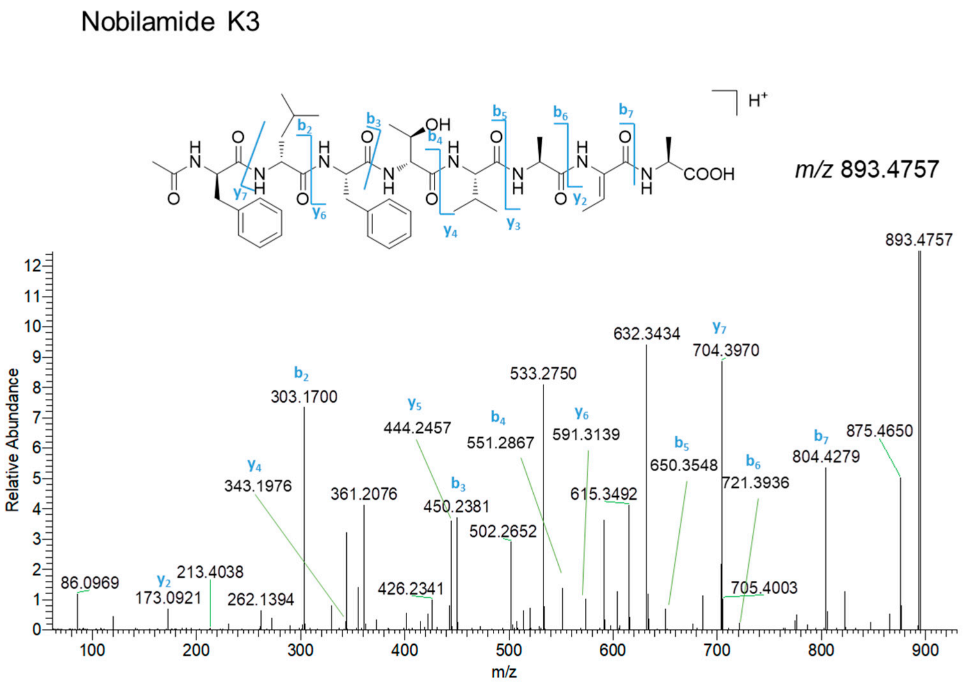

Figure S78. HRMS[2] spectrum of the [M+H]+ pseudomolecular ion of Nobilamide K3.

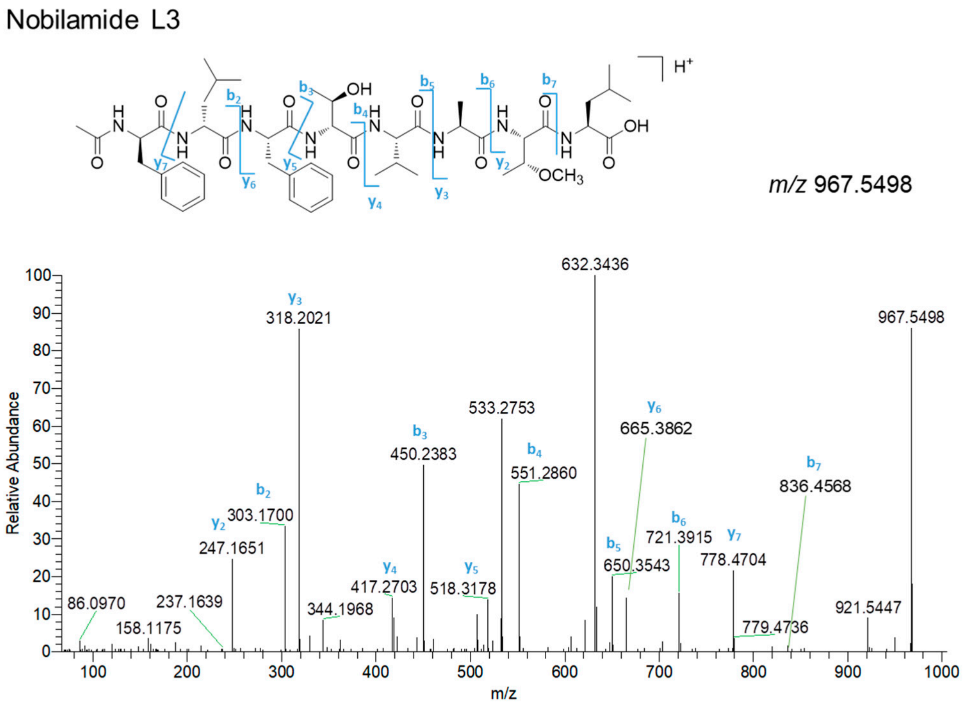

Figure S79. HRMS[2] spectrum of the [M+H]+ pseudomolecular ion of Nobilamide L3.

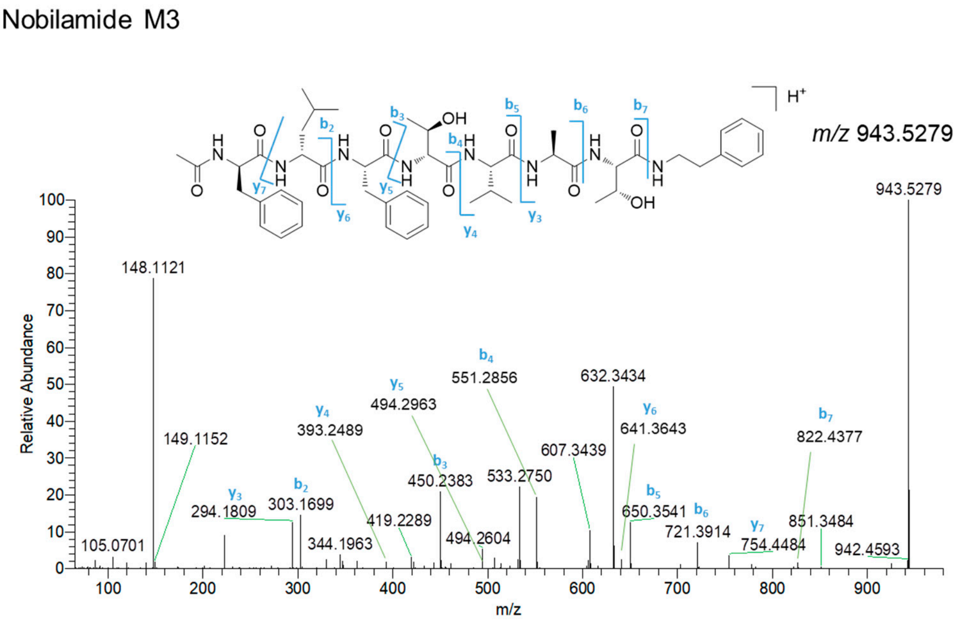

Figure S80. HRMS[2] spectrum of the [M+H]+ pseudomolecular ion of Nobilamide M3.

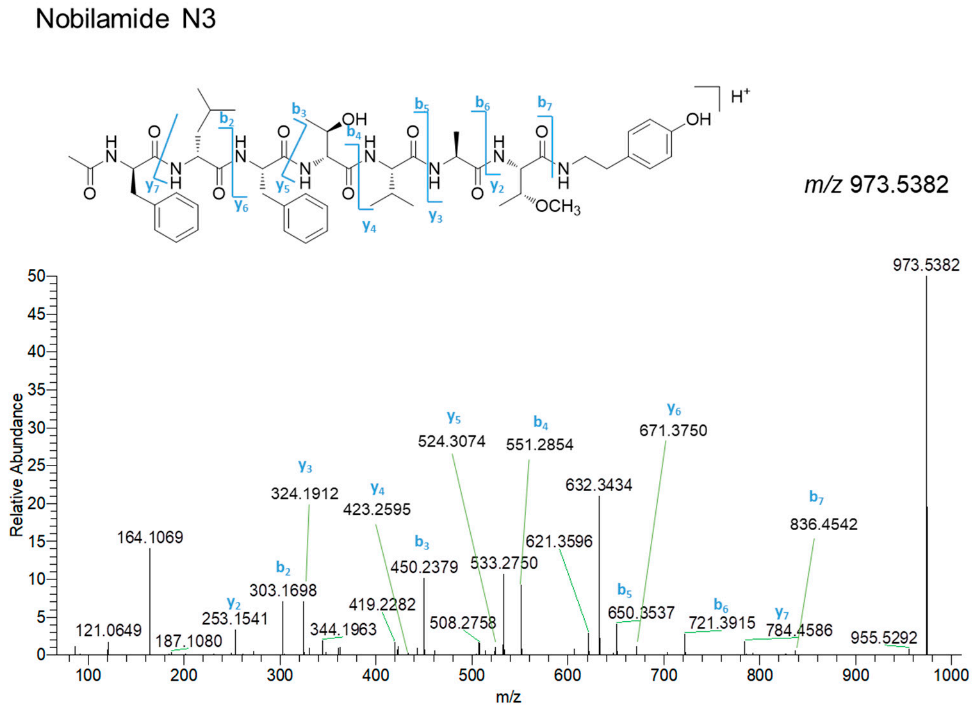

Figure S81. HRMS[2] spectrum of the [M+H]+ pseudomolecular ion of Nobilamide N3.

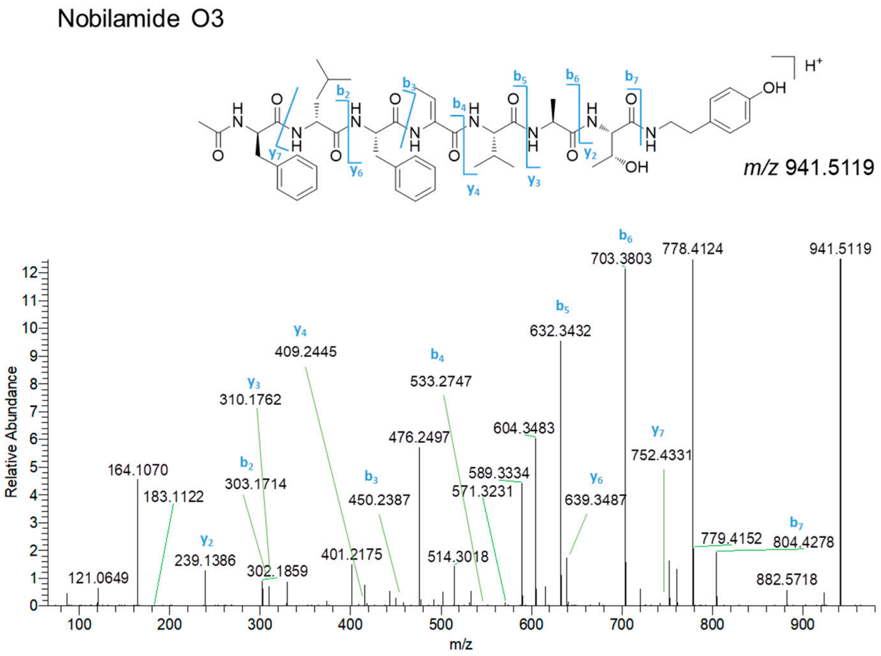

Figure S82. HRMS² spectrum of the $[M+H]^+$ pseudomolecular ion of Nobilamide O3.

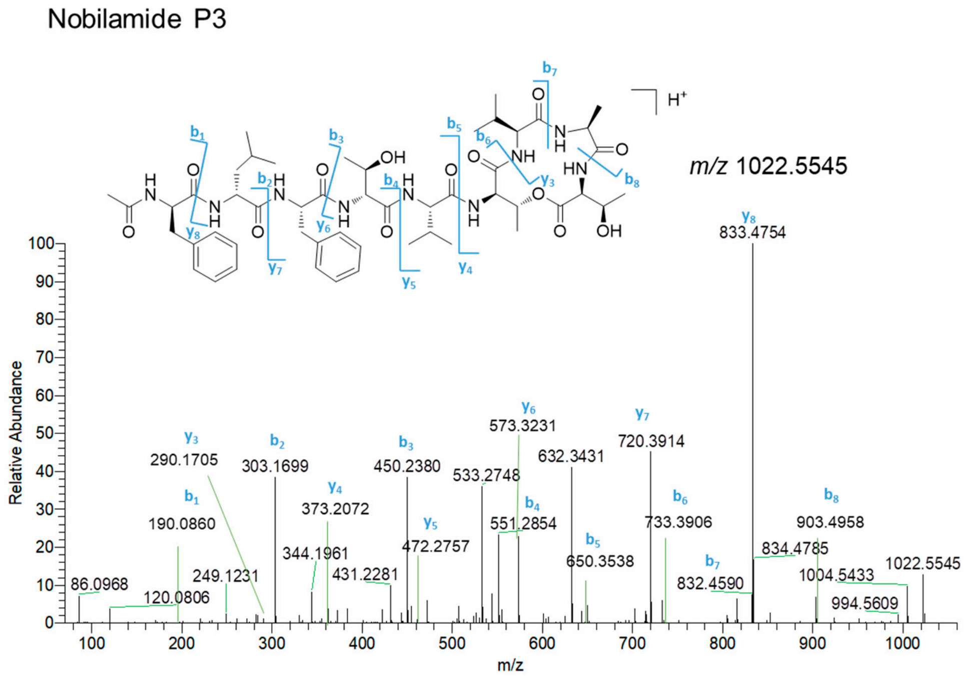

Figure S83. HRMS² spectrum of the $[M+H]^+$ pseudomolecular ion of Nobilamide P3.

Reference

1. Della Sala, G.; Mangoni, A.; Costantino, V.; Teta, R. Identification of the Biosynthetic Gene Cluster of Thermoactinoamides and Discovery of New Congeners by Integrated Genome Mining and MS-Based Molecular Networking. *Front. Chem.* **2020**, *8*, doi:10.3389/fchem.2020.00397.
